# Complexity and Resource Bound Analysis of Imperative Programs Using Difference Constraints

**DOI:** 10.1007/s10817-016-9402-4

**Published:** 2017-01-11

**Authors:** Moritz Sinn, Florian Zuleger, Helmut Veith

**Affiliations:** Institut für Informationssysteme, TU Wien, Favoritenstr. 9-11, Wien, Austria

**Keywords:** Bound analysis, Complexity analysis, Amortized analysis, Difference constraints, Static analysis, Resource bound analysis, Automatic complexity analysis, Cost analysis

## Abstract

Difference constraints have been used for termination analysis in the literature, where they denote relational inequalities of the form $x' \le y + c$, and describe that the value of *x* in the current state is at most the value of *y* in the previous state plus some constant $c \in \mathbb {Z}$. We believe that difference constraints are also a good choice for complexity and resource bound analysis because the complexity of imperative programs typically arises from counter increments and resets, which can be modeled naturally by difference constraints. In this article we propose a bound analysis based on difference constraints. We make the following contributions: (1) our analysis handles bound analysis problems of high practical relevance which current approaches cannot handle: we extend the range of bound analysis to a class of challenging but natural loop iteration patterns which typically appear in parsing and string-matching routines. (2) We advocate the idea of using bound analysis to infer invariants: our soundness proven algorithm obtains invariants through bound analysis, the inferred invariants are in turn used for obtaining bounds. Our bound analysis therefore does not rely on external techniques for invariant generation. (3) We demonstrate that difference constraints are a suitable abstract program model for automatic complexity and resource bound analysis: we provide efficient abstraction techniques for obtaining difference constraint programs from imperative code. (4) We report on a thorough experimental comparison of state-of-the-art bound analysis tools: we set up a tool comparison on (a) a large benchmark of real-world C code, (b) a benchmark built of examples taken from the bound analysis literature and (c) a benchmark of challenging iteration patterns which we found in real source code. (5) Our analysis is more scalable than existing approaches: we discuss how we achieve scalability.

**Electronic supplementary material:**

The online version of this article (doi:10.1007/s10817-016-9402-4) contains supplementary material, which is available to authorized users.

## Introduction

Automated program analysis for inferring program complexity and resource bounds is a very active area of research. Amongst others, approaches have been developed for analyzing functional programs [16], C# [15], C [2, 7, 29, 35], Java [1] and Integer Transition Systems [6, 10]. Below we sketch applications in the areas of verification and program understanding. For additional motivation we refer the reader to the cited papers.

*Verification* In many applications such as *embedded systems* there is a hard constraint on the availability of resources such as CPU time, memory, bandwidth, etc. It is an important part of functional correctness that programs stay within their given resource limits. As a concrete example we mention that considerable effort has been invested to analyze the* worst case execution time (WCET)* of hard real-time systems [33]. Another application domain is *security*, where the goal is to derive a bound on how much *secret information* is *leaked* in order to decide whether this leakage is acceptable [31].

*Static Profiling and Program Understanding* Standard profilers report numbers such as how often certain program locations are visited and how much time is spent inside certain functions; however, no information is provided how these numbers are related to the program input. Recently, new profiling approaches have been proposed that apply curve fitting techniques for deriving a cost function, which relates size measures on the program input to the measured program performance [9, 34]. We believe that automated complexity and resource bound analysis lends itself naturally as *static profiling technique*, because it provides the user with a symbolic expression that relates the program performance to the program input. In the same way, complexity and resource bound analysis can be used to explore unfamiliar code or to annotate library functions by their performance characteristics; we note that a substantial number of performance bugs can be attributed to a “wrong understanding of API performance features” [22].

As a final remark we discuss the relationship to *termination analysis*, which has been intensively studied in the last decade in the computer-aided verification community: complexity and resource bound analysis can be understood as a quantitative variant of termination analysis, where not only a qualitative “yes” answer is provided, but also a symbolic upper bound on the run-time of the program.

*Difference constraints* ($DCs$) have been introduced by Ben-Amram for termination analysis in [4], where they denote relational inequalities of the form $x' \le y + c$, and describe that the value of *x* in the current state is at most the value of *y* in the previous state plus some constant $c \in \mathbb {Z}$. We call a program whose transitions are given by a set of difference constraints a *difference constraint program* ($DCP$).

We advocate the use of $DCs$ for program complexity and resource bound analysis. Our key insight is that $DCs$ provide a natural abstraction of the standard manipulations of counters in imperative programs: counter *increments* and *decrements*, i.e., $x := x + c$ resp. *resets*, i.e., $x := y$, can be modeled by the $DCs x' \le x + c$ resp. $x' \le y$ (see Sect. 6 on program abstraction). The approach we discuss in this article exploits the expressive strength of $DCs$ and distinguishes between counter resets and counter increments in the reasoning. In contrast, previous approaches [1, 2, 6, 10, 15, 29, 35] to bound analysis are not able to track increments *and* resets on the same level of precision and therefore often fail to infer tight bounds for a class of nested loop constructs which we identified during our experiments on real-world code (demonstrated by our experimental evaluation in Sect. 8.3). In this article we make the following contributions:

1. Our analysis handles bound analysis problems of high practical relevance which current approaches cannot handle: we extend the range of bound analysis to a class of challenging but natural loop iteration patterns which typically appear in parsing and string-matching routines as we discuss in Sect. 2. At the same time our analysis is general and can handle most of the bound analysis problems which are discussed in the literature. Both claims are supported by our experiments.
2. We advocate the idea of using bound analysis to infer invariants: we state a clear and concise formulation of invariant analysis by bound analysis on base of our abstract program model: our soundness proven algorithm (Sect. 3) obtains invariants through bound analysis, the inferred invariants are in turn used for obtaining bounds. Our bound analysis therefore does not rely on external techniques for invariant generation.
3. We demonstrate that difference constraints are a suitable abstract program model for automatic complexity and resource bound analysis: we develop appropriate techniques for abstracting imperative programs to $DCPs$ in Sect. 6.
4. We report on a thorough experimental comparison of state-of-the-art bound analysis tools (Sect. 8): we set up a tool comparison on (a) a large benchmark of real-world C-code (Sect. 8.1), (b) a benchmark built of examples taken from the bound analysis literature (Sect. 8.2) and (c) a benchmark of challenging iteration patterns which we found in real source code (Sect. 8.3).
5. We have designed our analysis with the goal of scalability: our experiments demonstrate that our implementation outperforms the state-of-the-art with respect to scalability. We give a detailed discussion on how we achieve scalability in Sect. 10.

This article is an extension of the conference version presented at FMCAD 2015 [27]. Besides making the material more accessible through additional explanations and discussions, it adds the following contributions: (1) a discussion on the instrumentation of our analysis for *resource bound analysis* (Sect. 2.2). (2) A more detailed discussion and presentation of our *context-sensitive* bound algorithm (Sect. 3.3). (3) A more detailed discussion on how we determine local bounds and an extension to sets of local bounds (Sect. 4). (4) A complete example (Sect. 7). (5) A discussion on the relation to amortized complexity analysis (Sect. 9). (6) Additional experimental results (Sects. 8.2 and 8.3). (7) In Electronic Supplementary Material we state the soundness proofs omitted in the conference version.

## Motivation and Related Work

**Fig. 1:**
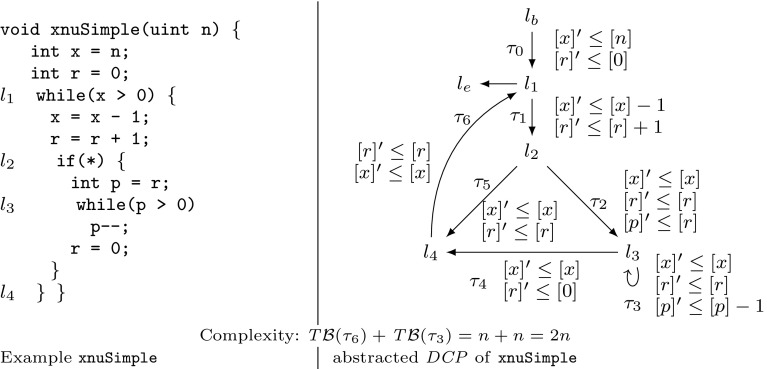
Running Example xnuSimple, the symbol *asterisk* denotes non-determinism (arising from conditions not modeled in the analysis)

Example xnuSimple stated in Fig. 1 is representative for a class of loops that we found in parsing and string matching routines during our experiments. In these loops the inner loop iterates over disjoint partitions of an array or string, where the partition sizes are determined by the program logic of the outer loop. For an illustration of this iteration scheme see Example xnu in Fig. 9 (Sect. 7), which contains a snippet of the source code after which we have modeled Example xnuSimple. Example xnuSimple has the linear *complexity* 2*n* (we define complexity here as the total number of loop iterations, for alternative definitions see the discussion in Sect. 2.2), because the inner loop as well as the outer loop can be iterated at most *n* times (as argued in the next paragraph). In the following, we give an overview how our approach infers the linear complexity for Example xnuSimple:

1. *Program Abstraction* We abstract the program to a $DCP$ over $\mathbb {N}$ as shown in Fig. 1. The abstract variable $[x]$ represents the program expression $\max (x,0)$. We discuss our algorithm for abstracting imperative programs to $DCP$s based on symbolic execution in Sect. 6.
2. *Finding Local Bounds* We identify $[p]$ as a variable that limits the number of executions of transition $\tau _3$: we have that $[p]$ decreases on each execution of $\tau _3$ ($[p]$ takes values over $\mathbb {N}$). We call $[p]$ a *local bound* for $\tau _3$. Accordingly we identify $[x]$ as a *local bound* for the transitions $\tau _1,\tau _{2},\tau _{4},\tau _{5},\tau _{6}$.
3. *Bound Analysis* Our algorithm (stated in Sect. 3) computes *transition bounds*, i.e., (symbolic) upper bounds on the number of times program transitions can be executed, and *variable bounds*, i.e., (symbolic) upper bounds on variable values. For both types of bounds, the main idea of our algorithm is to reason *how much* and *how often* the value of the local bound resp. the variable value may increase during program run. Our algorithm is based on a mutual recursion between variable bound analysis (“how much”, function $V\mathcal {B} (\mathtt {v})$) and transition bound analysis (“how often”, function $T\mathcal {B} (\tau )$). Next, we give an intuition how our algorithm computes transition bounds: for $\tau \in \{\tau _1,\tau _{2},\tau _{4},\tau _5,\tau _6\}$ our algorithm computes $T\mathcal {B} (\tau ) = [n] = n$ (note that $[n] = n$ because *n* has type *unsigned*) because the local bound $[x]$ is initially set to $[n]$ and never increased or reset. Our algorithm computes $T\mathcal {B} (\tau _3)$ ($\tau _3$ corresponds to the loop at $l_3$) as follows: $\tau _3$ has local bound $[p]$; $[p]$ is reset to $[r]$ on $\tau _{2}$; our algorithm detects that before each execution of $\tau _{2}$, $[r]$ is reset to $[0]$ on either $\tau _0$ or $\tau _4$, which we call the *context* under which $\tau _{2}$ is executed; our algorithm establishes that between being reset and flowing into $[p]$ the value of $[r]$ can be incremented up to $T\mathcal {B} (\tau _1)$ times by 1; our algorithm obtains $T\mathcal {B} (\tau _1) = n$ by a recursive call; finally, our algorithm calculates $T\mathcal {B} (\tau _3) = [0] + T\mathcal {B} (\tau _1) \times 1 = 0 + n \times 1 = n$. We give an example for the mutual recursion between $T\mathcal {B}$ and $V\mathcal {B}$ in Sect. 2.1.

### Invariants and Bound Analysis

**Fig. 2:**
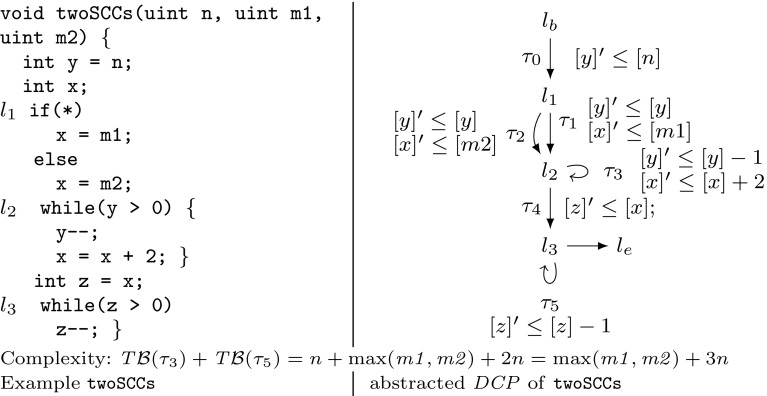
Running Example twoSCCs

We motivate the need for invariants in bound analysis and sketch how our algorithm infers invariants by bound analysis. Consider Example twoSCCs in Fig. 2. It is easy to infer *x* as a bound for the possible number of iterations of the loop at $l_3$. However, in order to obtain a bound in the *function parameters* the difficulty lies in finding an invariant of form $x \le \mathtt {expr}(n,{m_1},{m_2})$, where $\mathtt {expr}(n,{m_1},{m_2})$ denotes an expression over the function parameters $n,{m_1},{m_2}$. We show how our algorithm obtains the invariant $x \le \max (m_1,m_2) + 2n$ by means of bound analysis:

Our algorithm computes a transition bound for the loop at $l_3$ (with the single transition $\tau _5$) by $T\mathcal {B} (\tau _5) = T\mathcal {B} (\tau _4) \times V\mathcal {B} ([x]) = 1 \times V\mathcal {B} ([x]) = V\mathcal {B} ([x]) = T\mathcal {B} (\tau _3) \times 2 + \max ([m_1],[m_2]) = ( T\mathcal {B} (\tau _0) \times [n]) \times 2 + \max ([m_1],[m_2]) = (1 \times [n]) \times 2 + \max ([m_1],[m_2]) = 2n + \max (m_1,m_2)$ (note that $[n]=n$, $[m_1]=m_1$ and $[m_2] = m_2$ because $n, m_1, m_2$ have type *unsigned*). We point out the mutual recursion between $T\mathcal {B}$ and $V\mathcal {B}$: $T\mathcal {B} (\tau _5)$ has called $V\mathcal {B} (x)$, which in turn called $T\mathcal {B} (\tau _3)$. We highlight that the variable bound $V\mathcal {B} (x)$ (corresponding to the invariant $x \le \max (m_1,m_2) + 2n$) has been established during the computation of $T\mathcal {B} (\tau _5)$.

We call the kind of invariants that our algorithm infers *upper bound invariants* (Definition 6). We compare our reasoning to classical invariant analysis in Sect. 2.3.

### Resource Bound Analysis

We shortly discuss how *resource bound analysis* can be naturally formulated within our framework. We introduce a fresh variable *c* and add the initialization $c = 0$ to the beginning of the program under scrutiny. We add an increment/decrement $c = c + k$ at program locations where a resource of cost *k* is consumed (*k* is positive) or freed (*k* is negative). Resource bound analysis is then equivalent to computing an upper bound on the value of the variable *c*. We can run our algorithm $V\mathcal {B} (c)$ to compute a symbolic upper bound for *c*.

In the same way we can encode related bound analysis problems: *reachability bounds* [15] (visits to a single location), visits to multiple transitions, loop bounds or complexity analysis. For each of these bound analysis problems one can add a counter increment at the program locations of interest.

We illustrate the suggested encoding on the problem of computing loop bounds: for a given loop we add increments of the counter variable *c* to every back edge of the loop. Calling $V\mathcal {B} (c)$ then returns the sum of the transition bounds of all back edges of the loop. This example also illustrates how transition bounds are used for computing variable bounds in our approach.

### Related Work

*Termination* In [4] it is shown that termination of $DCPs$ is undecidable in general but decidable for the natural syntactic subclass of *fan-in free* $DCPs$ (see Definition 12), which is the class of $DCPs$ we use in this article. It is an open question for future work whether there is a complete algorithm for bound analysis of fan-in free $DCPs$.

*Bound Analysis* In [35] a bound analysis based on so-called *size-change constraints* $x' \lhd y$ is proposed, where $\lhd \in \{<,\le \}$. Size-change constraints form a strict syntactic subclass of $DCs$. However, termination is decidable even for size-change programs that are not fan-in free and a complete algorithm for deciding the complexity of size-change programs has been developed [8]. For reasoning about *inner loops* [35] computes *disjunctive loop* summaries while such summaries are not computed by the approach discussed in this work.

In [29] a bound analysis based on constraints of the form $x' \le x + c$ is proposed, where *c* is either an integer or a symbolic constant. Because the constraints in [29, 35] cannot model both increments *and* resets, the resulting bound analyses cannot infer the linear complexity of Example xnuSimple and need to rely on external techniques for invariant analysis.

The COSTA project (e.g. [1]) obtains recurrence relations from so-called *cost equations* using *invariant analysis* based on the *polyhedra abstract domain* and approaches from the literature for synthesizing *linear ranking functions*. Closed-form solutions for the obtained recurrence relations are inferred by means of *computer algebra*.

The technique discussed in [10] is based on the COSTA approach and formulated in terms of *cost equations*. Further, paper [10] is inspired by the counter instrumentation-based approach [14] and applies the techniques [3, 25] for inferring *linear ranking functions*. The technique of [10] achieves a high precision of the inferred bounds by means of control-flow refinement (see also Ref. [11]).

The technique discussed in [2] over-approximates the *reachable states* by *abstract interpretation* based on the *polyhedra abstract domain*. This information is used for generating a *linear constraint problem* from which a *multi-dimensional linear ranking function* is obtained. A bound on the number of values which can be taken by the ranking function is then obtained from the previously computed approximation of the reachable states. Importantly, the number of dimensions of the ranking function determines the degree of the bound polynomial. The approach of [2] therefore aims at inferring a ranking function with a *minimal* number of dimensions and thus depends on a *minimal* solution to the linear constraint problem which is obtained by *linear optimization* (Technique [2] instruments the LP-solver with an *objective function*).

The technique discussed in [6] applies approaches from the literature for *synthesizing ranking functions* thereby inferring bounds on the number of times the execution of isolated program parts can be repeated. These bounds, called *time bounds*, are then used to compute bounds on the absolute value of variables, so-called *variable size bounds*. Additional information is inferred through *abstract interpretation* based on the *octagon abstract domain*. An overall complexity bound is deduced by alternating between time bound and variable size bound analysis. In each alternation bounds for larger program parts are obtained based on the previously computed information.

In Sect. 8 we compare our implementation against the techniques [2, 6, 7, 10, 29].

*Amortized Complexity Analysis* We note that inferring the linear complexity 2*n* for Example xnuSimple, even though the inner loop can already be iterated *n* times *within* one iteration of the outer loop, is an instance of *amortized complexity analysis* [32]: the cost of executing the inner loop, *averaged* over all *n* iterations of the outer loop is 1. Most previous approaches [1, 6, 10, 15, 29, 35] can establish only a *quadratic* bound for Example xnuSimple. A typical reasoning which fails to establish the *linear complexity* of Example xnuSimple is as follows: (1) the outer loop can be iterated at most *n* times, (2) the inner loop can be iterated at most *n* times *within* one iteration of the outer loop (because the inner loop has a local loop bound *p* and $p \le n$ is an invariant), (3) the loop bound $n^2$ is obtained from (1) and (2) by multiplication.

The recent paper [7] discusses an interesting alternative for amortized complexity analysis of imperative programs: a system of linear inequalities is derived using Hoare-style proof-rules. Solutions to the system represent valid *linear* resource bounds. Since bound analysis typically does not aim at *some* bound but tries to infer a *tight* bound, Ref. [7] uses *linear optimization* (an LP-solver instrumented by an *objective function*) in order to obtain a *minimum solution* to the problem. Interestingly, Ref. [7] is able to compute the linear bound for $l_3$ of Example xnuSimple but fails to deduce the bound for the original source code (discussed in Sect. 7). Moreover, Ref. [7] is restricted to linear bounds, while our approach derives bounds which are polynomial (see, e.g., the results in Table 12) and which contain the maximum operator (e.g., Example twoSCCs). We compare our implementation to the implementation of Ref. [7] in Sect. 8.

*Invariants and Bound Analysis* The powerful idea of expressing locally computed bounds in terms of the function parameters by alternating between bound analysis and variable upper bound analysis has previously been applied in [6, 12, 28]. Since Refs. [12, 28] do not give a general algorithm but deal with specific cases we focus our discussion on [6] and highlight some important differences. The technique discussed in [6] computes upper bound invariants only for the *absolute* values of variables; for many cases, this does not allow to distinguish between variable increments and decrements: consider the program foo(int x, int y) {while(y> 0) {x $\mathtt{--}$ ; y $\mathtt{--}$ ;} while(x> 0) x $\mathtt{--}$ ;}. The algorithm described in [6] infers the bound $|x| + |y|$ for the second loop, whereas our analysis infers the bound $\max (x, 0)$. The approach of [6] depends on *global invariant analysis*. E.g., given a decrement $x := x - 1$, the technique of [6] needs to check whether $x \ge 0$ holds. If $x \ge 0$ cannot be ensured, the *decrement* can actually *increment* the *absolute* value of *x*, and will thus be interpreted as $|x| = |x| + 1$. This can either lead to gross over-approximations or failure of bound computation if the increment of |*x*| cannot be bounded. Since our approach does not track the *absolute* value but the value, it is not concerned with this problem. The technique discussed in [6] does not support *amortized analysis*: e.g., The technique [6] fails to compute the *linear bounds* for Example xnuSimple (Fig. 1), Example xnu (Fig. 9) and other examples we discuss in this article (see also the results in Sect. 8.3). On the other hand, Ref. [6] can infer bounds for functions with multiple recursive calls which is not supported by the analysis we present in this article.

*Comparison to Invariant Analysis* We contrast our previously discussed approach for computing a bound for the loop at $l_3$ of Example xnuSimple with classical invariant analysis: assume that we have added a counter *c* which counts the number of inner loop iterations (i.e., *c* is initialized to 0 and incremented in the inner loop). For inferring $c \le n$ through invariant analysis the invariant $c+x+r\le n$ is needed for the outer loop, and the invariant $c+x+p\le n$ for the inner loop. Both relate 3 variables and cannot be expressed as (parametrized) octagons (e.g., [26]). Further, the expressions $c+x+r$ and $c+x+p$ do not appear in the program, which is challenging for template based approaches to invariant analysis.

We now contrast our variable bound analysis (function $V\mathcal {B}$) with classical invariant analysis: reconsider Example twoSCCs in Fig. 2. We have discussed how our algorithm obtains the invariant $x \le \max (m_1,m_2) + 2n$ by means of bound analysis in the course of computing a bound for the loop at $l_3$. Note, that the invariant $x \le \max (m_1,m_2) + 2n$ cannot be computed by standard abstract domains such as *octagon* or *polyhedra*: these domains are *convex* and cannot express non-convex relations such as *maximum*. The most precise approximation of *x* in the polyhedra domain is $x \le m_1 + m_2 + 2n$. Unfortunately, it is well-known that the polyhedra abstract domain does not scale to larger programs and needs to rely on heuristics for termination. Standard *abstract domains* such as *octagon* or *polyhedra* propagate information *forward* until a fixed point is reached, *greedily* computing all possible invariants expressible in the abstract domain at every location of the program. In contrast, our method $V\mathcal {B} (x)$ infers the invariant $x \le \max (m1,m2) + 2n$ by *modular reasoning*: *local information* about the program (i.e., local bounds and increments/resets of variables) is combined to a *global* program property. Moreover, our variable and transition bound analysis is *demand-driven*: our algorithm performs only those recursive calls that are indeed needed to derive the desired bound. We believe that our analysis complements existing techniques for invariant analysis and will find applications outside of bound analysis.

## Program Model and Algorithm

In this section we present our algorithm for computing worst-case upper bounds on the number of executions of a given transition (transition bound) and on the value of a given program expression (variable bound and upper bound invariant).

### Definition 1

(*Program*) Let ${\varSigma }$ be a set of *states*. A *program* over ${\varSigma }$ is a directed labeled graph $\mathcal {P}= (L, T, l_b,l_e)$, where $L$ is a finite set of *locations*, $l_b \in L$ is the entry location, $l_e \in L$ is the exit location and $T\subseteq L\times 2^{{\varSigma }\times {\varSigma }} \times L$ is a finite set of *transitions*. We write $l_1 \xrightarrow {\lambda } l_2$ to denote a transition $(l_1,\lambda ,l_2) \in T$. We call $\lambda \in 2^{{\varSigma }\times {\varSigma }}$ a *transition relation*. A *path* of $\mathcal {P}$ is a sequence $l_0 \xrightarrow {\lambda _0} l_1 \xrightarrow {\lambda _1} \cdots$ with $l_i \xrightarrow {\lambda _i} l_{i+1} \in E$ for all *i*. A *run* of $\mathcal {P}$ is a sequence $\rho = (l_b,\sigma _0) \xrightarrow {\lambda _0} (l_1,\sigma _1) \xrightarrow {\lambda _1} \cdots$ such that $l_b \xrightarrow {\lambda _0} l_1 \xrightarrow {\lambda _1} \cdots$ is a path of $\mathcal {P}$ and for all $0 < i$ it holds that $(\sigma _{i-1},\sigma _i) \in \lambda _{i-1}$. A run $\rho$ is *complete* if it ends at $l_e$.

Note that a *run* of $\mathcal {P}= (L, T, l_b,l_e)$ starts at location $l_b$. Further note that we call an *edge* ${l_1 \xrightarrow {\lambda } l_1} \in T$ of the program a *transition*, whereas $\lambda$ is its *transition relation*. In the following we will refer to *transitions* by $\tau$ and to *transition relations* by $\lambda$.

*Transition bounds* are at the core of our analysis: we infer bounds on the number of loop iterations, on computational complexity, on resource consumption, etc., by computing bounds on the number of times that one or several transitions can be executed. Before we formally define our notion of a transition bound we have to introduce some notation.

### Definition 2

(*Counter Notation I*) Let $\mathcal {P}(L, T,l_b, l_e)$ be a program over ${\varSigma }$. Let $\tau \in T$. Let $\rho = (l_b,\sigma _0) \xrightarrow {\lambda _0} (l_1,\sigma _1) \xrightarrow {\lambda _1} \cdots$ be a run of $\mathcal {P}$. By $\sharp (\tau , \rho )$ we denote the number of times that $\tau$ occurs on $\rho$.

In the following, we denote by ‘$\infty$’ a value s.t. $a < \infty$ for all $a \in \mathbb {Z}$ (infinity).

### Definition 3

(*Transition Bound*) Let $\mathcal {P}= (L, T, l_b,l_e)$ be program over states ${\varSigma }$. Let $\tau \in T$. A value $\mathtt {b}\in \mathbb {N}_0 \cup \{\infty \}$ is a bound for $\tau$ on a run $\rho = (l_{b}, \sigma _0) \xrightarrow {\lambda _0} (l_{1}, \sigma _1) \xrightarrow {\lambda _1} (l_{2}, \sigma _2) \xrightarrow {\lambda _2} \cdots$ of $\mathcal {P}$ iff $\sharp (\tau ,\rho ) \le \mathtt {b}$, i.e., iff $\tau$ appears not more than $\mathtt {b}$ times on $\rho$. A function $\mathtt {b}: {\varSigma }\rightarrow \mathbb {N}_{0} \cup \{\infty \}$ is a *bound* for $\tau$ iff for all runs $\rho$ of $\mathcal {P}$ it holds that $\mathtt {b}(\sigma _0)$ is a bound for $\tau$ on $\rho$, where $\sigma _0$ denotes the initial state of $\rho$.

Given a program transition $\tau$, our bound algorithm (which we define below) computes a *bound* for $\tau$. If possible, the bound computed by our algorithm should be *precise* or *tight*, in particular the trivial bound ${\varSigma }\rightarrow \infty$ is (most often) of no value to us.

### Definition 4

(*Precise Transition Bound*) Let $\mathcal {P}(L, T,l_b, l_e)$ be a program over states ${\varSigma }$. Let $\tau \in T$. We say that a transition bound $\mathtt {b}: {\varSigma }\rightarrow \mathbb {N}_{0} \cup \{\infty \}$ for $\tau$ is *precise* iff for each $\sigma _0 \in {\varSigma }$ there is a run $\rho = (l_b,\sigma _0) \xrightarrow {\lambda _0} (l_1,\sigma _1) \xrightarrow {\lambda _1} \cdots$ such that $\sharp (\tau ,\rho ) = \mathtt {b}(\sigma _0)$.

*Informally* A transition bound is *precise* if it can be reached for all initial states $\sigma _0$. Note that there is exactly *one* precise transition bound.

### Definition 5

(*Tight Transition Bound*) Let $\mathcal {P}(L, T,l_b, l_e)$ be a program over states ${\varSigma }$. Let $\tau \in T$. We say that a transition bound $\mathtt {b}: {\varSigma }\rightarrow \mathbb {N}_{0} \cup \{\infty \}$ is *tight* iff there is a $c > 0$ such that either (1) for all $\sigma \in {\varSigma }$ we have $\mathtt {b}(\sigma ) < c$ ($\mathtt {b}$ is bounded), or (2) there is a family of states $(\sigma _{i})_{i\in \mathbb {N}}$ with $\lim \nolimits _{i\mapsto {\infty }}\mathtt {b}(\sigma _i) = \infty$ ($\mathtt {b}$ is unbounded) such that for all $\sigma _i$ there is a run $\rho _i$ starting in $\sigma _i$ with $\mathtt {b}(\sigma _i) \le c \times \sharp (\tau ,\rho _i)$.

*Informally* A transition bound is *tight* if it is in the same *asymptotic class* as the *precise* transition bound: let $\tau \in T$. For the special case ${\varSigma }= \mathbb {N}$ we have the following: let $f: \mathbb {N} \rightarrow \mathbb {N}$ denote the *precise* transition bound for $\tau$. Let $g: \mathbb {N} \rightarrow \mathbb {N}$ be *some* transition bound for $\tau$. Trivially $f \in O(g)$ (*f* does not grow faster than *g*). Now, *g* is *tight* if also $f \in {\varOmega }(g)$ (*f* does not always grow slower than *g*). With $f \in O(g)$ and $f \in {\varOmega }(g)$ we have that $f \in {\varTheta }(g)$. The same can be formulated for general state sets ${\varSigma }$ by mapping ${\varSigma }$ to the natural numbers.

We discussed in Sect. 2.1 that in the course of computing transition bounds, our analysis computes *invariants* of a special shape. We now formally define the form of the invariants that our analysis infers.

### Definition 6

(*Upper Bound Invariant*) Let $\mathcal {P}(L, T,l_b,l_e)$ be a program over ${\varSigma }$. Let $e: {\varSigma }\rightarrow \mathbb {Z}$. Let $l\in L$. Let $\rho = (l_{b}, \sigma _0) \xrightarrow {\lambda _0} (l_{1}, \sigma _1) \xrightarrow {\lambda _1} (l_{2}, \sigma _2) \xrightarrow {\lambda _2} \cdots$ be a run of $\mathcal {P}$. A value $\mathtt {b}\in \mathbb {Z} \cup \{\infty \}$ is an *upper bound invariant* for $e$ at $l$ on $\rho$ iff $e(\sigma _i) \le \mathtt {b}$ holds for all *i* on $\rho$ with $l_i = l$. A function $\mathtt {b}: {\varSigma }\rightarrow \mathbb {Z} \cup \{\infty \}$ is an *upper bound invariant* for $e$ at $l$ iff for all runs $\rho$ of $\mathcal {P}$ it holds that $\mathtt {b}(\sigma _0)$ is an *upper bound invariant* for $e$ at $l$ on $\rho$, where $\sigma _0$ denotes the initial state of $\rho$.

We now formally define the notion *local bound* that we motivated in Sect. 2.

### Definition 7

(*Counter Notation II*) Let $\mathcal {P}(L, T,l_b, l_e)$ be a program over ${\varSigma }$. Let $\rho = (l_b,\sigma _0) \xrightarrow {\lambda _0} (l_1,\sigma _1) \xrightarrow {\lambda _1} \cdots$ be a run of $\mathcal {P}$. Let $e: {\varSigma }\rightarrow \mathbb {Z}$ be a norm. By ${\downarrow }(e, \rho )$ we denote the number of times that the value of $e$ decreases on $\rho$, i.e., ${\downarrow }(e,\rho ) = |\{i \mid {e(\sigma _i) > e(\sigma _{i+1}) }\}|$.

### Definition 8

(*Norm*) Let ${\varSigma }$ be a set of *states*. A *norm* $e: {\varSigma }\rightarrow \mathbb {Z}$ over ${\varSigma }$ is a function that maps the states to the integers.

### Definition 9

(*Local Bound*) Let $\mathcal {P}(L, T,l_b, l_e)$ be a program over ${\varSigma }$. Let $\tau \in T$. Let $e: {\varSigma }\rightarrow \mathbb {N}$ be a *norm* that takes values in the *natural numbers*. Let $\rho = (l_b,\sigma _0) \xrightarrow {u_0} (l_1,\sigma _1) \xrightarrow {u_1} \cdots$ be a run of $\mathcal {P}$. $e$ is a *local bound* for $\tau$ on $\rho$ if it holds that $\sharp (\tau , \rho ) \le {\downarrow }(e, \rho )$. We call $e$ a local bound for $\tau$ if $e$ is a local bound for $\tau$ on all runs of $\mathcal {P}$.

*Discussion* A natural number valued norm $e$ is a *local bound* for $\tau$ on a run $\rho$ if $\tau$ appears not more often on $\rho$ than the number of times the value of $e$ decreases. I.e., a *local* bound $e$ for $\tau$ limits the number of executions of $\tau$ on a run $\rho$ as long as certain program parts (those were $e$ increases) are not executed. We argue in Sect. 9 that in our analysis *local bounds* play the role of *potential functions* in classical *amortized complexity analysis* [32]. We discuss how we obtain local bounds in Sect. 4.

### Difference Constraint Programs

As discussed introductory, we base our algorithm on the abstract program model of *difference constraint programs* which we now formally define in Definition 12. We discuss in Sect. 6 how we abstract a given program to a $DCP$.

#### Definition 10

(*Variables, Symbolic Constants, Atoms*) By $\mathcal {V}$ we denote a finite set of *variables*. By $\mathcal {C}$ we denote a finite set of symbolic *constants*. $\mathcal {A}= \mathcal {V}\cup \mathcal {C}$ is the set of *atoms*.

#### Definition 11

(*Difference Constraints*) A *difference constraint* over $\mathcal {A}$ is an inequality of form $x^\prime \le y + c$ with $x \in \mathcal {V}$, $y \in \mathcal {A}$ and $c \in \mathbb {Z}$. By $\mathcal {DC}(\mathcal {A})$ we denote the set of all difference constraints over $\mathcal {A}$.

*Notation* We often write $x^\prime \le y$ as a shorthand for the difference constraint $x^\prime \le y + \mathtt {c}$.

#### Definition 12

(*Difference Constraint Program, Syntax*) A *difference constraint program* ($DCP$) over $\mathcal {A}$ is a directed labeled graph ${\varDelta }\mathcal {P}= (L, E, l_b,l_e)$, where $L$ is a finite set of vertices, $l_b \in L$ and $l_e \in L$ and $E\subseteq L\times 2^{\mathcal {DC}(\mathcal {A})} \times L$ is a finite set of edges. We write $l_1 \xrightarrow {u} l_2$ to denote an edge $(l_1, u, l_2) \in E$ labeled by a set of difference constraints $u\in 2^{\mathcal {DC}(\mathcal {A})}$. We use the notation $l_1 \xrightarrow {} l_2$ to denote an edge that is labeled by the empty set of difference constraints. ${\varDelta }\mathcal {P}$ is *fan-in-free*, if for every edge $l_1 \xrightarrow {u} l_2 \in E$ and every $\mathtt {v}\in \mathcal {V}$ there is at most one $\mathtt {a}\in \mathcal {A}$ and $\mathtt {c}\in \mathbb {Z}$ s.t. $\mathtt {v}^\prime \le \mathtt {a}+ \mathtt {c}\in u$.

*Example* Figure 10b shows a fan-in free $DCP$.

#### Definition 13

(*Difference Constraint Program, Semantics*) The set of *valuations* of $\mathcal {A}$ is the set $Val _\mathcal {A}= \mathcal {A}\rightarrow \mathbb {N}$ of mappings from $\mathcal {A}$ to the natural numbers. Let $u\in 2^{\mathcal {DC}(\mathcal {A})}$. We define $\llbracket u\rrbracket \in 2^{( Val _\mathcal {A}\times Val _\mathcal {A})}$ s.t. $(\sigma ,\sigma ^\prime ) \in \llbracket u\rrbracket$ iff for all $x^\prime \le y + c \in u$ it holds that (i) $\sigma ^\prime (x) \le \sigma (y) + c$ and (ii) for all $\mathtt {s}\in \mathcal {C}$ $\sigma ^\prime (\mathtt {s}) = \sigma (\mathtt {s})$. A $DCP$ ${\varDelta }\mathcal {P}= (L, E, l_b,l_e)$ is a *program* over the set of states $Val _\mathcal {A}$ with locations $L$, entry location $l_b$, exit location $l_e$ and transitions $T= \{l_1 \xrightarrow {\llbracket u\rrbracket } l_2 \mid l_1 \xrightarrow {u} l_2 \in E\}$.

*Discussion* A $$ DCP \ $$ is a *program* (Definition 1) whose *transition relations* are solely specified by conjunctions of *difference constraints*. Note that variables in difference constraint programs take values only over the *natural numbers*. Further note that we refer to the syntactic representation of the transition relation in form of a set of difference constraints by $u$, whereas by $\llbracket u\rrbracket$ we refer to the transition relation itself.

#### Definition 14

(*Well-defined* $DCP$) Let ${\varDelta }\mathcal {P}= (L, E, l_b,l_e)$ be a $DCP$ over atoms $\mathcal {A}$. We say that a variable $x\in \mathcal {V}$ *is defined at* $l$ if $x \in \mathtt {def}(l)$, where $\mathtt {def}: L\rightarrow 2^\mathcal {A}$ is defined by $\mathtt {def}(l) = \bigcap \nolimits _{{l_1 \xrightarrow {u} l} \in E} \{x \mid \exists y \in \mathcal {V}~\exists \mathtt {c}\in \mathbb {Z} \text { s.t. } {x^\prime \le y + \mathtt {c}\in u}\} \cup \mathcal {C}$.

We say that a variable *x* *is used at* $l$ if $x \in \mathtt {use}(l)$, where $\mathtt {use}: L\rightarrow 2^\mathcal {A}$ is defined by $\mathtt {use}(l) = \bigcup \nolimits _{{l\xrightarrow {u} l_1} \in E} \{y \mid \exists x \in \mathcal {A}~\exists \mathtt {c}\in \mathbb {Z} \text { s.t. } {x^\prime \le y + \mathtt {c}\in u}\}$.

${\varDelta }\mathcal {P}$ is *well-defined* iff $l_b$ has no incoming edges and for all $l\in L$ it holds that $\mathtt {use}(l) \subseteq \mathtt {def}(l)$.

*Discussion* A $DCP$ ${\varDelta }\mathcal {P}$ is well-defined if $l_b$ has no incoming edges and for all $\mathtt {v}\in \mathcal {V}$ it holds that $\mathtt {v}$ is defined at all locations at which $\mathtt {v}$ is used (symbolic constants are always defined). Note that for well-defined programs we in particular require $\mathtt {use}(l_b) \subseteq \mathtt {def}(l_b)$. Because $l_b$ has no incoming edges we have $\mathtt {def}(l_b) = \mathcal {C}$. Thus only symbolic constants can be used at $l_b$.

Throughout this work we will only consider $DCP$s that are *fan-in free* and *well-defined*.

Let ${\varDelta }\mathcal {P}(L, E,l_b, l_e)$ be a $DCP$ over $\mathcal {A}$. Our bound algorithm, which we start to develop in the next section, computes a *bound* for a given transition $\tau \in E$ in form of an expression over $\mathcal {A}$ which involves the operators $+$,$\times$, / ,$\min$,$\max$ and the floor function $\lfloor \cdot \rfloor$. However, note that the *norms*, which are treated as *atoms* (elements of $\mathcal {A}$) in the abstraction, can involve arbitrary operators (see Sect. 6).

#### Definition 15

(*Expressions over* $\mathcal {A}$) By $Expr (\mathcal {A})$ we denote the set of expressions over $\mathcal {A}\cup \mathbb {Z} \cup \{\infty \}$ that are formed using the arithmetical operators addition ($+$), multiplication ($\times$), maximum ($\max$), minimum ($\min$) and integer division of form $\lfloor \frac{\mathtt {expr}}{\mathtt {c}}\rfloor$ where $\mathtt {expr}\in Expr (\mathcal {A})$ and $\mathtt {c}\in \mathbb {N}$. The semantics function $\llbracket \cdot \rrbracket : Expr (\mathcal {A}) \rightarrow ( Val _\mathcal {A}\rightarrow \mathbb {Z} \cup \{\infty \})$ evaluates an expression $\mathtt {expr}\in Expr (\mathcal {A})$ over a state $\sigma \in Val _\mathcal {A}$ using the usual operator semantics (we have $a + \infty = \infty$, $\min (a, \infty ) = a$, etc.).

Our bound algorithm, which we define next, computes a special case of an *upper bound invariant* which we call a *variable bound*.

#### Definition 16

(*Variable Bound*) Let ${\varDelta }\mathcal {P}(L, E,l_b, l_e)$ be a $DCP$ over $\mathcal {A}$. Let $\mathtt {a}\in \mathcal {A}$. We call $\mathtt {b}$ s.t. $\mathtt {b}$ is an *upper bound invariant* for $\llbracket \mathtt {a}\rrbracket$ at all $l\in L$ with $\mathtt {a}\in \mathtt {def}(l)$ a *variable bound* for $\mathtt {a}$.

Let variable *x* of the abstract program represent the expression $\mathtt {expr}$ of the concrete program. Note that by computing a *variable bound* for *x* in the abstract program, we compute an *upper bound invariant* for $\mathtt {expr}$ in the concrete program.

### Algorithm

Our bound algorithm computes a bound for a given transition $\tau \in E$ based on a mapping $\zeta : E\rightarrow Expr (\mathcal {A})$ (called *local bound mapping*) which assigns each transition $\tau \in E$ either (1) a *bound* for $\tau$ in form of an expression over the symbolic constants (i.e., $\zeta (\tau ) \in Expr (\mathcal {C})$) or (2) a *local bound* for $\tau$ in form of a variable (i.e, $\zeta (\tau ) \in \mathcal {V}$). Note that $\mathcal {V}\cap Expr (\mathcal {C}) = \emptyset$. In Case (1) our algorithm (Definition 19) returns $T\mathcal {B} (\tau ) = \zeta (\tau )$. In Case (2) a transition bound $T\mathcal {B} (\tau ) \in Expr (\mathcal {C})$ is computed by inferring *how often* and by *how much* the local transition bound $\zeta (\tau ) \in \mathcal {V}$ of $\tau$ may increase during program run.

#### Definition 17

(*Local Bound Mapping*) Let ${\varDelta }\mathcal {P}(L, E,l_b, l_e)$ be a $DCP$ over $\mathcal {A}$. Let $\rho = (l_b,\sigma _0) \xrightarrow {u_0} (l_1,\sigma _1) \xrightarrow {u_1} \cdots$ be a run of ${\varDelta }\mathcal {P}$. We call a function $\zeta : E\rightarrow Expr (\mathcal {A})$ a *local bound mapping* for $\rho$ if for all $\tau \in E$ it holds that either

1. $\zeta (\tau ) \in Expr (\mathcal {C})$ and $\llbracket \zeta (\tau )\rrbracket (\sigma _0)$ is a *bound* for $\tau$ on $\rho$ or
2. $\zeta (\tau ) \in \mathcal {V}$ and $\llbracket \zeta (\tau )\rrbracket$ is a *local bound* for $\tau$ on $\rho$.
  We say that $\zeta$ *is a local bound mapping* for ${\varDelta }\mathcal {P}$ if $\zeta$ is a local bound mapping for all runs of ${\varDelta }\mathcal {P}$.

Further, our bound algorithm is based on a syntactic distinction between two kinds of updates that modify the value of a given variable $\mathtt {v}\in \mathcal {V}$: we identify transitions which *increment* $\mathtt {v}$ and transitions which *reset* $\mathtt {v}$.

#### Definition 18

(*Resets and Increments*) Let ${\varDelta }\mathcal {P}= (L, E, l_b,l_e)$ be a $DCP$ over $\mathcal {A}$. Let $\mathtt {v}\in \mathcal {V}$. We define the *resets* $\mathcal {R}(\mathtt {v})$ and *increments* $\mathcal {I}(\mathtt {v})$ of $\mathtt {v}$ as follows:

$$\begin{aligned} \mathcal {R}(\mathtt {v})= & {} \{(l_1 \xrightarrow {u} l_2,\mathtt {a},\mathtt {c}) \in {E\times \mathcal {A}\times \mathbb {Z}} \mid {\mathtt {v}^\prime \le \mathtt {a}+ \mathtt {c}} \in u, \mathtt {a}\ne \mathtt {v}\}\nonumber \\ \mathcal {I}(\mathtt {v})= & {} \{(l_1 \xrightarrow {u} l_2, \mathtt {c}) \in {E\times \mathbb {N}} \mid {\mathtt {v}^\prime \le \mathtt {v}+ \mathtt {c}} \in u, \mathtt {c}> 0\} \end{aligned}$$

Given a path $\pi$ of ${\varDelta }\mathcal {P}$ we say that $\mathtt {v}$ is *reset* on $\pi$ if there is a transition $\tau$ on $\pi$ such that $(\tau , \mathtt {a}, \mathtt {c}) \in \mathcal {R}(\mathtt {v})$ for some $\mathtt {a}\in \mathcal {A}$ and $\mathtt {c}\in \mathbb {Z}$. We say that $\mathtt {v}$ is *incremented* on $\pi$ if there is a transition $\tau$ on $\pi$ such that $(\tau , \mathtt {c}) \in \mathcal {I}(\mathtt {v})$ for some $\mathtt {c}\in \mathbb {N}$.

I.e., we have that $(\tau , \mathtt {a}, \mathtt {c}) \in \mathcal {R}(\mathtt {v})$ if variable $\mathtt {v}$ is reset to a value smaller or equal to $\mathtt {a}+ \mathtt {c}$ when executing the transition $\tau$. Accordingly we have $(\tau , \mathtt {c}) \in \mathcal {I}(\mathtt {v})$ if variable $\mathtt {v}$ is incremented by a value smaller or equal to $\mathtt {c}$ when executing the transition $\tau$.

Our algorithm in Definition 19 is built on a *mutual recursion* between the two functions $V\mathcal {B} (\mathtt {v})$ and $T\mathcal {B} (\tau )$, where $V\mathcal {B} (\mathtt {v})$ infers a *variable bound* for variable $\mathtt {v}$ and $T\mathcal {B} (\tau )$ infers a *transition bound* for the transition $\tau$.

#### Definition 19

(*Bound Algorithm*) Let ${\varDelta }\mathcal {P}(L, E, l_b, l_e)$ be a $DCP$ over $\mathcal {A}$. Let $\zeta : E\rightarrow Expr (\mathcal {A})$. We define $V\mathcal {B} : \mathcal {A}\mapsto Expr (\mathcal {A})$ and $T\mathcal {B} : E\mapsto Expr (\mathcal {A})$ as:

$$\begin{aligned} V\mathcal {B} (\mathtt {a})&= \mathtt {a}\text {, if }\mathtt {a}\in \mathcal {C}\text {, else}\\ V\mathcal {B} (\mathtt {v})&= \mathtt {Incr}(\mathtt {v}) + \max \limits _{(\_, \mathtt {a}, \mathtt {c}) \in \mathcal {R}(\mathtt {v})} ( V\mathcal {B} (\mathtt {a}) + \mathtt {c})\\ T\mathcal {B} (\tau )&= \zeta (\tau )\text {, if }\zeta (\tau ) \not \in \mathcal {V}\text {, else}\\ T\mathcal {B} (\tau )&= \mathtt {Incr}(\zeta (\tau )) + \sum \limits _{( t ,\mathtt {a},\mathtt {c}) \in \mathcal {R}(\zeta (\tau ))} T\mathcal {B} ( t ) \times \max ( V\mathcal {B} (\mathtt {a}) + \mathtt {c}, 0) \end{aligned}$$

where $\mathtt {Incr}(\mathtt {v}) = \sum \nolimits _{(\tau ,\mathtt {c}) \in \mathcal {I}(\mathtt {v})}{ T\mathcal {B} (\tau ) \times \mathtt {c}}$ (we set $\mathtt {Incr}(\mathtt {v}) = 0$ for $\mathcal {I}(\mathtt {v}) = \emptyset$)

*Discussion* We first explain the subroutine $\mathtt {Incr}(\mathtt {v})$: with $(\tau , \mathtt {c}) \in \mathcal {I}(\mathtt {v})$ we have that a single execution of $\tau$ *increments* the value of $\mathtt {v}$ by not more than $\mathtt {c}$. $\mathtt {Incr}(\mathtt {v})$ multiplies the bound for $\tau$ with the increment $\mathtt {c}$ in order to summarize the total amount by which $\mathtt {v}$ may be incremented over all executions of $\tau$. $\mathtt {Incr}(\mathtt {v})$ thus computes a bound on the total amount by which the value of $\mathtt {v}$ may be *incremented* during program run.

The function $V\mathcal {B} (\mathtt {v})$ computes a variable bound for $\mathtt {v}$: after executing a transition $\tau$ with $(\tau , \mathtt {a}, \mathtt {c}) \in \mathcal {R}(\mathtt {v})$, the value of $\mathtt {v}$ is bounded by $V\mathcal {B} (\mathtt {a}) + \mathtt {c}$. As long as $\mathtt {v}$ is not *reset*, its value cannot increase by more than $\mathtt {Incr}(\mathtt {v})$.

The function $T\mathcal {B} (\tau )$ computes a transition bound for $\tau$ based on the following reasoning: (1) the total amount by which the local bound $\zeta (\tau )$ of transition $\tau$ can be *incremented* is bounded by $\mathtt {Incr}(\zeta (\tau ))$. (2) We consider a reset $( t , \mathtt {a}, \mathtt {c}) \in \mathcal {R}(\zeta (\tau ))$; in the worst case, a single execution of $t$ resets the local bound $\zeta ( t )$ to $V\mathcal {B} (\mathtt {a}) + \mathtt {c}$, adding $\max ( V\mathcal {B} (\mathtt {a}) + \mathtt {c},0)$ to the potential number of executions of $t$; in total all $T\mathcal {B} ( t )$ possible executions of $t$ add up to $T\mathcal {B} ( t ) \times \max ( V\mathcal {B} (\mathtt {a}) + \mathtt {c},0)$ to the potential number of executions of $t$.

**Table 1:**
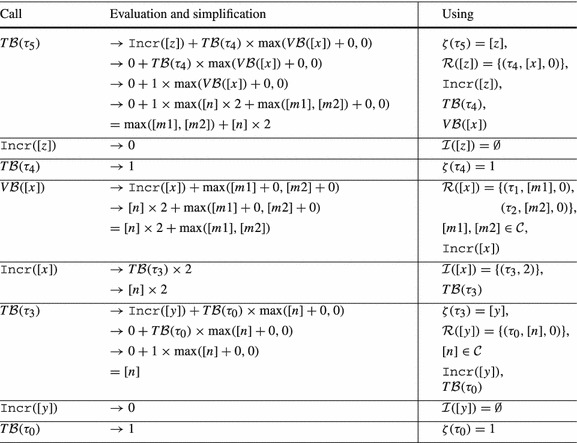
Computation of $T\mathcal {B} (\tau _5)$ for Example twoSCCs (Fig. 2) by Definition 19

*Example* We want to infer a bound for the loop at $l_3$ in Fig. 2. We thus compute a transition bound for $\tau _5$ (the single back edge of the loop at $l_3$). See Table 1 for details on the computation. We get $T\mathcal {B} (\tau _5) = \max ([m1],[m2]) + [n] \times 2$. Thus $\max (m1,m2) + 2n$ is a bound for the loop at $l_3$ ($m_1$, $m_2$ and *n* have type *unsigned*).

*Termination* Our algorithm does not terminate iff recursive calls cycle, i.e., if a call to $T\mathcal {B} (\tau )$ resp. $V\mathcal {B} (\mathtt {v})$ (indirectly) leads to a recursive call to $T\mathcal {B} (\tau )$ resp. $V\mathcal {B} (\mathtt {v})$. This can be detected easily, we return the expression ‘$\infty$’.

We distinguish three cases of cyclic computation: (1) there is a variable $\mathtt {v}\in \mathcal {V}$ such that the computation of $V\mathcal {B} (\mathtt {v})$ ends up calling $V\mathcal {B} (\mathtt {v})$ over a number of recursive calls to $V\mathcal {B}$. (2) There is a transition $\tau \in E$ such that the computation of $T\mathcal {B} (\tau )$ ends up calling $T\mathcal {B} (\tau )$ over a number of recursive calls to $T\mathcal {B}$. (3) There is a variable $\mathtt {v}\in \mathcal {V}$ and a transition $\tau \in E$ such that the computation of $T\mathcal {B} (\tau )$ calls $V\mathcal {B} (\mathtt {v})$ which in turn ends up calling $T\mathcal {B} (\tau )$ over a number of recursive calls to $V\mathcal {B}$ and $T\mathcal {B}$.

**Case (1)** occurs iff there is a cycle in the *reset graph* (Definition 20 in Sect. 3.3) of ${\varDelta }\mathcal {P}$. In Sect. 3.4 we discuss a preprocessing that ensures absence of cycles in the reset graph and thus absence of Case (1) by renaming the program variables appropriately.

**Case (2)** occurs iff there is a transition $\tau _1$ with local bound *x* that increases the local bound *y* of a transition $\tau _2$ which in turn increases *x*. We conclude that absence of Case (2) is ensured if for all *strongly connected components* (SCC) $\mathtt {SCC}$ of ${\varDelta }\mathcal {P}$ we can find an ordering $\tau _1,\ldots ,\tau _n$ of the transitions of $\mathtt {SCC}$ such that the local bound of transition $\tau _i$ is not increased on any transition $\tau _j$ with $n \ge j > i \ge 1$. Note that the existence of such an ordering for each SCC of ${\varDelta }\mathcal {P}$ proves termination of ${\varDelta }\mathcal {P}$: it allows to directly compose a termination proof in form of a *lexicographic ranking function* by ordering the respective local transition bounds accordingly.

**Fig. 3:**
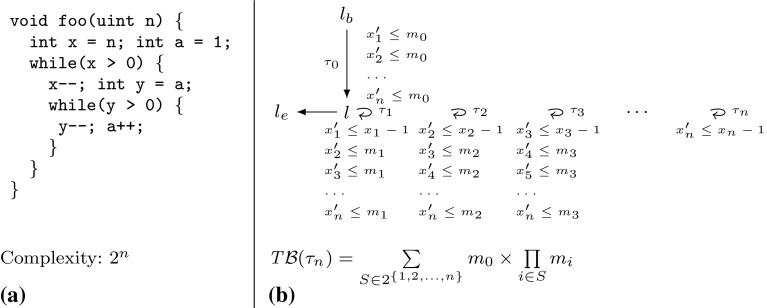
**a** Example with an exponential loop bound, **b** Example for which we obtain a bound expression of *exponential size*, transitions $\tau _{1\le i \le n}$ have source- and target-location $l$

An example for Case (3) is given in Fig. 3a. Let $\tau _1$ be the transition on which *y* is reset to *a*. Let $\tau _2$ be the single transition of the inner loop. Assume we want to compute a loop bound for the inner loop, i.e., a transition bound for $\tau _2$ with local bound *y*. This triggers a variable bound computation for *a* because *y* is reset to *a*. Since *a* is incremented on $\tau _2$, the variable bound computation for *a* will in turn trigger a transition bound computation for $\tau _2$. Note, however, that the loop bound for the inner loop is *exponential* ($2^n$). We consider exponential loop bounds very rare, we did not encounter an exponential loop bound during our experiments.

*Complexity* Our algorithm can be efficiently (polynomial in the number of variables and transitions of the abstract program) implemented using caches (dynamic programming): we set $\zeta (\tau ) = T\mathcal {B} (\tau )$ after having computed $T\mathcal {B} (\tau )$. Accordingly we introduce a cache to store the result of a $V\mathcal {B}$-computation. When $V\mathcal {B} (\mathtt {v})$ is called we first check if the result is already in the cache before performing the computation. The computed bound expressions, however, can be of exponential size: consider the $DCP$ ${\varDelta }\mathcal {P}=(\{l_b,l\}, \{\tau _0,\tau _1,\ldots ,\tau _n\},l_b,l_e)$ over variables $\{x_1,x_2,\ldots ,x_n\}$ and constants $\{m_1,m_2,\ldots ,m_n\}$ shown in Fig. 3b. In fact, $T\mathcal {B} (\tau _n) = \sum \nolimits _{S \in 2^{\{1,2,\ldots ,n\}}} m_0 \times \prod \nolimits _{i \in S} m_i$ is *precise* for Fig. 3b. However, the example is artificial. To our experience the computed bound expressions can, in practice, be reduced to human readable size by applying basic rules of arithmetic.

#### Theorem 1

(Soundness) Let ${\varDelta }\mathcal {P}(L, E, l_b, l_e)$ be a well-defined and fan-in free $DCP$ over atoms $\mathcal {A}$. Let $\zeta : E\mapsto Expr (\mathcal {A})$ be a *local bound mapping* for ${\varDelta }\mathcal {P}$. Let $\mathtt {a}\in \mathcal {A}$ and $\tau \in E$. Let $T\mathcal {B} (\tau )$ and $V\mathcal {B} (\mathtt {a})$ be as defined in Definition 19. We have: (1) $\llbracket T\mathcal {B} (\tau )\rrbracket$ is a *transition bound* for $\tau$. (2) $\llbracket V\mathcal {B} (\mathtt {a})\rrbracket$ is a *variable bound* for $\mathtt {a}$.

In the following we describe two straightforward improvements of the algorithm stated in Definition 19.

*Improvement I* Let $\tau \in E$. Let $\mathtt {v}\in \mathcal {V}$ be a *local bound* for $\tau$, i.e., for all runs $\rho$ of ${\varDelta }\mathcal {P}$ we have that $\sharp (\tau ,\rho ) \le {\downarrow }(\mathtt {v},\rho )$. Let $\mathtt {c}\in \mathbb {N}$. Let ${\downarrow }(\mathtt {v}, \mathtt {c}, \rho )$ denote the number of times that the value of $\mathtt {v}$ decreases on a run $\rho$ of ${\varDelta }\mathcal {P}$ by at least $\mathtt {c}$ (refines Definition 7). If for all runs $\rho$ of ${\varDelta }\mathcal {P}$ we have that $\sharp (\tau ,\rho ) \le {\downarrow }(\mathtt {v},\mathtt {c},\rho )$ (refines Definition 9) then $\llbracket \lfloor \frac{ T\mathcal {B} (\tau )}{\mathtt {c}}\rfloor \rrbracket$ is a *bound* for $\tau$ (assuming $\zeta (\tau ) = \mathtt {v}$). In our simple abstract program model, $\mathtt {c}\in \mathbb {N}$ is obtained syntactically from a constraint $\mathtt {v}^\prime \le \mathtt {v}- \mathtt {c}$. See Sect. 4 on how we determine relevant constraints. More details on the discussed improvement are given in [30].

*Improvement II* Let $\tau _1,\tau _2 \in E$ be two transitions with the same local bound, i.e., $\zeta (\tau _1) = \zeta (\tau _2)$. If $\tau _1$ and $\tau _2$ cannot be executed without decreasing the common local bound $\zeta (\tau _1)$ *twice*, once for $\tau _1$ and once for $\tau _2$ (e.g., $\tau _2$ and $\tau _5$ in xnuSimple, Fig. 1), we have that $\sharp (\tau _1,\rho ) + \sharp (\tau _2,\rho ) \le \llbracket T\mathcal {B} (\tau _1)\rrbracket (\sigma _0) = \llbracket T\mathcal {B} (\tau _2)\rrbracket (\sigma _0)$. Thus, $T\mathcal {B} (\tau _1)$ is a bound on the number of times that $\tau _1$ *and* $\tau _2$ can be executed on any run of ${\varDelta }\mathcal {P}$. We exploit this observation: assume some $\mathtt {v}\in \mathcal {V}$ is incremented by $\mathtt {c}_1$ on $\tau _1$ and by $\mathtt {c}_2$ on $\tau _2$. For computing $\mathtt {Incr}(\mathtt {v})$ we only add $T\mathcal {B} (\tau _1) \times \max \{\mathtt {c}_1,\mathtt {c}_2\}$ instead of $T\mathcal {B} (\tau _1) \times \mathtt {c}_1 + T\mathcal {B} (\tau _2) \times \mathtt {c}_2$. This idea can be generalized to multiple transitions. Further details on the discussed improvement are given in [30].

### Reasoning Based on Reset Chains

**Fig. 4:**
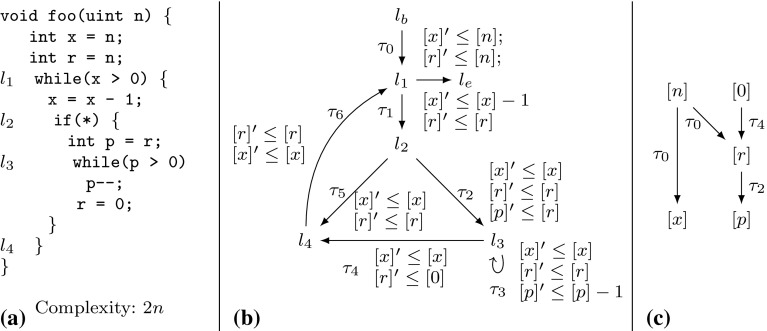
**a** Example, **b** Abstraction, **c** Reset Graph

Consider Fig. 4. The precise bound for the loop at $l_3$ is *n*: Initially *r* has value *n*, after we have iterated the loop at $l_3$, *r* is set to 0. Thus the loop can only be executed in at most one iteration of the outer loop. However, our algorithm from Definition 19 infers a quadratic bound for the loop at $l_3$: as shown in Table 2 we have $T\mathcal {B} (\tau _3) = [n] \times [n]$. We thus get $n^2$ (*n* has type *unsigned*) as bound for the loop at $l_3$ in the concrete program.

**Table 2:**
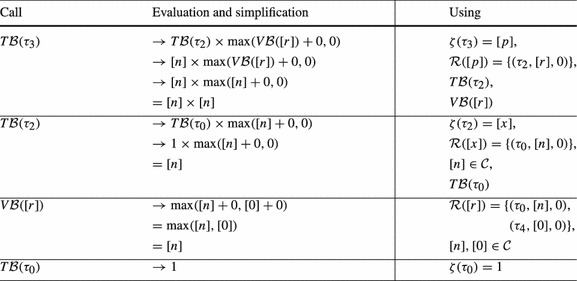
Computation of $T\mathcal {B} (\tau _3)$ for Fig. 4b by Definition 19 (without calls to $\mathtt {Incr}$ because $\mathcal {I}(\mathtt {v}) = \emptyset$ and thus $\mathtt {Incr}(\mathtt {v}) = 0$ for Fig. 4b)

Our algorithm from Definition 19 does not take into account that *r* is reset to 0 after executing the loop at $l_3$. In the following we discuss an extension of our algorithm which overcomes this imprecision by taking the *context* under which a transition is executed into account: we say that a transition $\tau _2$ is executed under *context* $\tau _1$ if transition $\tau _1$ was executed before the current execution of $\tau _2$ and after the previous execution of $\tau _2$ (if any).

As an example, consider Fig. 4b, the abstraction of Fig. 4a. We have that $\tau _2$ is always executed either under context $\tau _0$ or under context $\tau _4$. When executing $\tau _2$ under context $\tau _0$, *p* is set to *n*. But when executing $\tau _2$ under context $\tau _4$, *p* is set to 0. Moreover, $\tau _2$ can only be executed once under context $\tau _0$ because $\tau _0$ is executed only once.

We define the notion of a *reset graph* as a means to reason systematically about the context under which *resets* can be executed.

#### Definition 20

(*Reset Chain Graph*) Let ${\varDelta }\mathcal {P}(L, E,l_b,l_e)$ be a $DCP$ over $\mathcal {A}$. The *reset chain graph* or *reset graph* of ${\varDelta }\mathcal {P}$ is the directed graph $\mathcal {G}$ with node set $\mathcal {A}$ and edges $\mathcal {E}= \{(y, \tau , \mathtt {c}, x) \mid {(\tau , y, \mathtt {c}) \in \mathcal {R}(x)}\} \subseteq \mathcal {A}\times E\times \mathbb {Z} \times \mathcal {V}$, i.e., each edge has a label in $E\times \mathbb {Z}$. We call $\mathcal {G}(\mathcal {A},\mathcal {E})$ a *reset chain DAG* or *reset DAG* if $\mathcal {G}(\mathcal {A},\mathcal {E})$ is acyclic. We call $\mathcal {G}(\mathcal {A},\mathcal {E})$ a *reset chain forest* or *reset forest* if the sub-graph $\mathcal {G}(\mathcal {V},\mathcal {E})$ (recall that $\mathcal {V}\subset \mathcal {A}$) is a forest. We call a *finite* path $\kappa = \mathtt {a}_n \xrightarrow {\tau _n, c_n} \mathtt {a}_{n-1} \xrightarrow {\tau _{n-1}, c_{n-1}} \cdots \mathtt {a}_0$ in $\mathcal {G}$ with $n > 0$ a *reset chain* of ${\varDelta }\mathcal {P}$. We say that $\kappa$ is a reset chain from $\mathtt {a}_n$ to $\mathtt {a}_0$. Let $n \ge i \ge j \ge 0$. By $\kappa _{[i,j]}$ we denote the sub-path of $\kappa$ that starts at $\mathtt {a}_i$ and ends at $\mathtt {a}_j$. We define $in (\kappa ) = \mathtt {a}_n$, $c (\kappa ) = \sum \nolimits _{i=1}^n c_i$, $trn (\kappa ) = \{\tau _n,\tau _{n-1},\ldots ,\tau _1\}$, and $atm (\kappa ) = \{a_{n-1},\ldots ,a_0\}$. $\kappa$ is *sound* if for all $1 \le i < n$ it holds that $\mathtt {a}_i$ is *reset* on all paths from the target location of $\tau _1$ to the source location of $\tau _i$ in ${\varDelta }\mathcal {P}$. $\kappa$ is *optimal* if $\kappa$ is sound and there is no sound reset chain $\varkappa$ of length $n+1$ s.t. $\varkappa _{[n,0]} = \kappa$. Let $\mathtt {v}\in \mathcal {V}$, by $\mathfrak {R}(\mathtt {v})$ we denote the set of *optimal reset chains* ending in $\mathtt {v}$.

*Example* Figure 4c shows the reset graph of Fig. 4b.

We elaborate on the notions *sound* and *optimal* below. Let us first state a basic intuition on how we employ reset chains to enhance the precision of our reasoning:

For a given reset $(\tau , \mathtt {a}, \mathtt {c}) \in \mathcal {R}(\mathtt {v})$, the reset graph determines which atom flows into variable $\mathtt {v}$ under which context. For example, consider Fig. 4b and its reset graph in Fig. 4c: when executing the reset $(\tau _2,[r],0) \in \mathcal {R}([p])$ under the context $\tau _4$, $[p]$ is set to $[0]$, if the same reset is executed under the context $\tau _0$, $[p]$ is set to $[n]$. Note that the reset graph does not represent *increments* of variables. We discuss how we handle increments in Sect. 3.3.1.

Let $\mathtt {v}\in \mathcal {V}$. Given a reset chain $\kappa$ of length *n* that ends at $\mathtt {v}$, we say that $( trn (\kappa ), in (\kappa ), c (\kappa ))$ is a reset of $\mathtt {v}$ with context of length $n-1$. I.e., $\mathcal {R}(\mathtt {v})$ from Definition 18 is the set of *context-free* resets of $\mathtt {v}$ (context of length 0), because $( trn (\kappa ), in (\kappa ), c (\kappa )) \in \mathcal {R}(\mathtt {v})$ iff $\kappa$ ends at $\mathtt {v}$ and has length 1. Our previously defined algorithm from Definition 19 uses only *context-free* resets, we say that it reasons *context free*. For reasoning with context, we substitute the term

$$\begin{aligned} \sum \limits _{( t ,\mathtt {a},\mathtt {c}) \in \mathcal {R}(\zeta (\tau ))} T\mathcal {B} ( t ) \times \max ( V\mathcal {B} (\mathtt {a}) + \mathtt {c}, 0) \end{aligned}$$

in Definition 19 by the term

$$\begin{aligned} \sum \limits _{\kappa \in \mathfrak {R}(\zeta (\tau ))} T\mathcal {B} ( trn (\kappa )) \times \max ( V\mathcal {B} ( in (\kappa )) + c (\kappa ), 0). \end{aligned}$$

Note that we can compute a bound on the number of times that a sequence $\tau _1,\tau _2,\ldots ,\tau _n$ of transitions may occur on a run by computing $\min \nolimits _{1\le i \le n} T\mathcal {B} (\tau _i)$.

We now discuss how our algorithm infers the *linear* bound for $\tau _3$ of Fig. 4 when applying the described modification to Definition 19: the reset graph of Fig. 4b is shown in Fig. 4c. There are 3 reset chains ending in $[p]$: $\kappa _1 = [0] \xrightarrow {\tau _4,0} [r] \xrightarrow {\tau _2,0} [p]$, $\kappa _2 = [n] \xrightarrow {\tau _0,0} [r] \xrightarrow {\tau _2,0} [p]$ and $\kappa _3 = [r] \xrightarrow {\tau _2,0} [p]$. However, $\kappa _3$ is a sub-path of $\kappa _1$ and $\kappa _2$. Note that $\kappa _1$ and $\kappa _2$ are *sound* by Definition 20 because $[r]$ is reset on all paths from the target location $l_3$ of $\tau _2$ to the source location $l_2$ of $\tau _2$ in Fig. 4b (namely on $\tau _4$). $\kappa _1$ and $\kappa _2$ are both *optimal* because they are sound and of maximal length (we discuss the notions *sound* and *optimal* next). Thus $\mathfrak {R}([p]) = \{\kappa _1, \kappa _2\}$. Basing our analysis on $\mathfrak {R}([p])$ rather than $\mathcal {R}([p])$ our approach reasons as shown in Table 3. We get $T\mathcal {B} (\tau _3) = [n]$, i.e., we get the bound *n* (*n* has type *unsigned*) for the loop at $l_3$ in the concrete program (Fig. 4a).

**Table 3:**
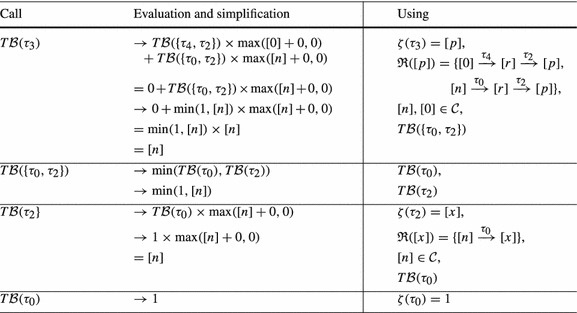
Computation of $T\mathcal {B} (\tau _3)$ for Fig. 4b by Definition 21 (without calls to $\mathtt {Incr}$ because $\mathcal {I}(\mathtt {v}) = \emptyset$ and thus $\mathtt {Incr}(\mathtt {v}) = 0$ for Fig. 4b)

*Sound and Optimal Reset Paths* A given reset chain $\mathtt {a}_n \xrightarrow {\tau _n, \mathtt {c}_n} \mathtt {a}_{n-1} \xrightarrow {\tau _{n-1}, \mathtt {c}_{n-1}} \cdots \xrightarrow {\tau _1,\mathtt {c}_1} \mathtt {a}_0$ is *sound* if in between any two executions of $\tau _1$ all atoms on the path (but not necessarily $\mathtt {a}_n$ where the path starts and $\mathtt {a}_0$ where it ends) are *reset*: Assume that *r* in Fig. 4a would not be reset after executing the inner loop. Then we could repeat the reset of *p* to *r* without resetting *r* to 0, and the inner loop would have a *quadratic * loop bound. For the abstract program the described modification replaces the constraint $[r]^\prime \le [0]$ on $\tau _4$ in Fig. 4b by $[r]^\prime \le [r]$. In the modified program, $[r]$ is not reset between two executions of $\tau _2$, i.e., the reset chain $[n] \xrightarrow {\tau _0} [r] \xrightarrow {\tau _2} [p]$ is not sound. Our algorithm therefore reasons based on the reset chain $[r] \xrightarrow {\tau _2} [p]$ and obtains a quadratic bound for $\tau _3$: $T\mathcal {B} (\tau _3) = T\mathcal {B} (\tau _2) \times V\mathcal {B} (r) = [n] \times [n]$. I.e., if *r* is not *reset* on the outer loop this is modeled in our analysis by considering the reset chain $[r] \xrightarrow {\tau _2} [p]$ rather than the maximal reset chain $[n] \xrightarrow {\tau _0} [r] \xrightarrow {\tau _2} [p]$. Considering the *maximal* reset chain $[n] \xrightarrow {\tau _0} [r] \xrightarrow {\tau _2} [p]$ would be unsound in the described scenario: $\min ( T\mathcal {B} (\tau _0), T\mathcal {B} (\tau _2)) \times [n] = [n]$ is *not* a valid transition bound for $\tau _3$ if *r* is not reset to 0 between two executions of the inner loop. The *optimal* reset chains are the sound reset chains with *maximal* context, i.e., those reset chains that are sound and cannot be extended without becoming *unsound*.

#### Algorithm Based on Reset Chain Forests

In the presence of cycles in the reset graph we get infinitely many reset chains. Let us for now assume that the given program has a *reset forest*, i.e., that the sub-graph of the reset graph, which has nodes only in $\mathcal {V}$, is a forest (Definition 20). Then also the complete reset graph is *acyclic* because $\mathcal {A}= \mathcal {V}\cup \mathcal {C}$ and the nodes in $\mathcal {C}$ cannot have incoming edges (Definition 20).

##### Definition 21

(*Bound Algorithm using Reset Chains (reset forest)*) Let $\zeta : E\rightarrow Expr (\mathcal {A})$ be a *local bound mapping* for ${\varDelta }\mathcal {P}$. Let $V\mathcal {B} : \mathcal {A}\mapsto Expr (\mathcal {A})$ be as defined in Definition 19. We override the definition of $T\mathcal {B} : E\mapsto Expr (\mathcal {A})$ in Definition 19 by stating:

$$\begin{aligned} T\mathcal {B} (\tau )&= \zeta (\tau )\text {, if } \zeta (\tau ) \not \in \mathcal {V}\text {, else}\\ T\mathcal {B} (\tau )&= \mathtt {Incr}\left( \bigcup \limits _{\kappa \in \mathfrak {R}(\zeta (\tau ))} atm (\kappa )\right) \\&~~~~~~~~~~~~+ \sum \limits _{\kappa \in \mathfrak {R}(\zeta (\tau ))} T\mathcal {B} ( trn (\kappa )) \times \max ( V\mathcal {B} ( in (\kappa )) + c (\kappa ), 0) \end{aligned}$$

where $$ T\mathcal {B} (\{\tau _1,\tau _2,\ldots ,\tau _n\}) = \min \nolimits _{1 \le i \le n} T\mathcal {B} (\tau _i)$$ and

$$\mathtt {Incr}(\{\mathtt {a}_1,\mathtt {a}_2,\ldots ,\mathtt {a}_n\}) = \sum \nolimits _{1 \le i \le n}\mathtt {Incr}(\mathtt {a}_i)$$ with $$\mathtt {Incr}(\emptyset ) = 0$$

**Table 4:**
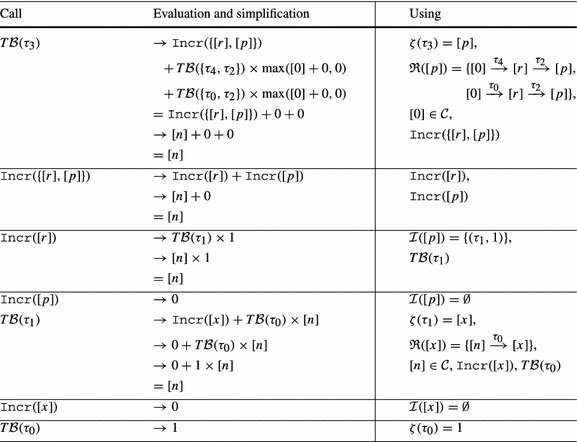
Computation of $T\mathcal {B} (\tau _3)$ for Example xnuSimple (Fig. 1) by Definition 21

*Discussion and Example* We have discussed above why we replace the term $T\mathcal {B} ( t ) \times \max ( V\mathcal {B} (\mathtt {a}) + \mathtt {c}, 0)$ from Definition 19 by the term $T\mathcal {B} ( trn (\kappa )) \times \max ( V\mathcal {B} ( in (\kappa )) + c (\kappa ), 0)$. We further discuss the term $\mathtt {Incr}(\bigcup \nolimits _{\kappa \in \mathfrak {R}(\zeta (\tau ))} atm (\kappa ))$ which replaces the term $\mathtt {Incr}(\zeta (\tau ))$ from Definition 19: consider Example xnuSimple in Fig. 1. Note that *r* may be incremented on $\tau _1$ between the reset of *r* to 0 on $\tau _0$ resp. $\tau _4$ and the reset of *p* to *r* on $\tau _{2}$. The term $\mathtt {Incr}(\bigcup \nolimits _{\kappa \in \mathfrak {R}(\zeta (\tau ))} atm (\kappa ))$ takes care of such increments which may increase the value that finally flows into $\zeta (\tau )$ (in the example *p*) when the last transition on $\kappa$ (in the example $\tau _{2}$) is executed. In Table 4 the details of the bound computation are given. We get $T\mathcal {B} (\tau _3) = [n]$, i.e, we have the bound *n* for the loop at $l_3$ in the concrete program (Fig. 1a, *n* has type *unsigned*).

**Fig. 5:**
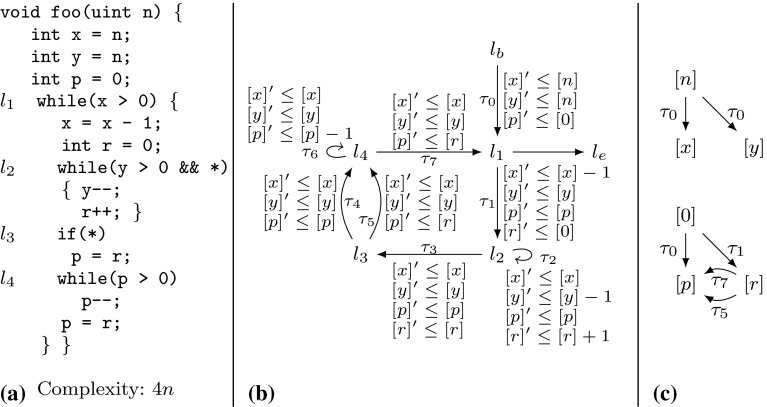
**a** Example, **b** Abstraction, **c** Reset Graph

*Soundness* Definition 21 for $DCP$s with a reset forest is a special case of Definition 23 for $DCP$s with a reset DAG. We prove soundness of Definition 23 in Electronic Supplementary Material.

*Complexity* The nodes of a reset forest are the variables and constants of the abstract program (the elements of $\mathcal {A}$). Since the number of paths of a forest is polynomial in the number of nodes, the run time of our algorithm remains polynomial.

#### Algorithm Based on Reset Chain DAGs

The examples we considered so far had reset forests. (Note that the definition of a *reset forest* (Definition 20) only requires the sub-graph over the variables, i.e., the reset graph without the nodes that are symbolic constants, to be a *forest*.) In the following we generalize Definition 21 to reset DAGs. We discuss in Sect. 3.4 how we ensure that the reset graph is *acyclic*.

Consider the Example shown in Fig. 5. The outer loop (at $l_1$) can be executed *n* times. The loop at $l_4$ resp. transition $\tau _6$ can be executed 2*n* times, e.g., by executing the program as depicted in Table 5:

The first row counts the number of iterations of the outer loop, the second row shows the transitions that are executed and in the last two rows the values of *r* resp. *p* are tracked. The execution switches between two iteration schemes of the outer loop: an uneven iteration increments *r* twice (by executing $\tau _2$ twice) and afterward assigns *r* to *p* by executing $\tau _5$. We can then execute $\tau _6$ two times. Afterward the value of *r* is “saved” in *p* for the next (even) iteration of the outer loop before *r* is set to 0 on $\tau _1$. Therefore $\tau _6$ can be executed again two times in the next, even iteration though *r* is not incremented on that iteration.

**Table 5:**
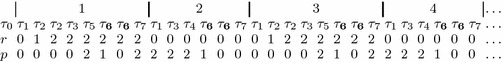
Run of Figure 5

Consider the abstracted $DCP$ in Fig. 5b and its reset graph in Fig. 5c. We have that $\kappa _2 = [0] \xrightarrow {\tau _1} [r] \xrightarrow {\tau _5} [p]$ and $\kappa _3 = [0] \xrightarrow {\tau _1} [r] \xrightarrow {\tau _7} [p]$ are two reset chains ending in $[p]$ (see Fig. 5 c). Observe that both are sound, i.e., between any two executions of $\tau _7$ resp. $\tau _5$ $[r]$ is reset. However, $[r]$ is *not* necessarily reset between the execution of $\tau _5$ and $\tau _7$, therefore the accumulated value 2 of *r* is used twice to increase the local bound $[p]$ of $\tau _6$.

I.e., since there are two paths from $[r]$ to $[p]$ in the reset graph (Fig. 5c) we have to count the increments of $[r]$ twice: once for $\kappa _2$ and once for $\kappa _3$. Definition 22 distinguishes between nodes that have a single resp. multiple path(s) to a given variable in the reset graph. This is used in Definition 23 for a sound handling of the latter case.

##### Definition 22

($atm _1(\kappa )$ *and* $atm _2(\kappa )$) Let ${\varDelta }\mathcal {P}(L, E,l_b,l_e)$ be a $DCP$ over $\mathcal {A}$. Let $P(\mathtt {a},\mathtt {v})$ denote the set of paths from $\mathtt {a}$ to $\mathtt {v}$ in the *reset graph* of ${\varDelta }\mathcal {P}$. Let $\mathtt {v}\in \mathcal {V}$. Let $\kappa$ be a reset chain ending in $\mathtt {v}$. We define $atm _1(\kappa ) = \{\mathtt {a}\in atm (\kappa ) \mid |P(\mathtt {a},\mathtt {v})| \le 1\}$ and $atm _2(\kappa ) = \{\mathtt {a}\in atm (\kappa ) \mid |P(\mathtt {a},\mathtt {v})| > 1\}$, where |*S*| denotes the number of elements in *S*.

##### Definition 23

(*Bound Algorithm Based on Reset Chains (reset DAG)*) Let ${\varDelta }\mathcal {P}(L, E,l_b,l_e)$ be a $DCP$ over $\mathcal {A}$. Let $\zeta : E\rightarrow Expr (\mathcal {A})$ be a *local bound mapping* for ${\varDelta }\mathcal {P}$. Let $V\mathcal {B} : \mathcal {A}\mapsto Expr (\mathcal {A})$ be as defined in Definition 19. We override the definition of $T\mathcal {B} : E\mapsto Expr (\mathcal {A})$ in Definition 19 by stating:

$$\begin{aligned} T\mathcal {B} (\tau )&= \zeta (\tau )\text {, if } \zeta (\tau ) \not \in \mathcal {V}\text {, else}\\ T\mathcal {B} (\tau )&= \mathtt {Incr}\left( \bigcup \limits _{\kappa \in \mathfrak {R}(\zeta (\tau ))} atm _1(\kappa )\right) \\&~~~~~~~+~\sum \limits _{\kappa \in \mathfrak {R}(\zeta (\tau ))} T\mathcal {B} ( trn (\kappa )) \times \max ( V\mathcal {B} ( in (\kappa )) + c (\kappa ), 0) + \mathtt {Incr}( atm _2(\kappa )) \end{aligned}$$

where $$ T\mathcal {B} (\{\tau _1,\tau _2,\ldots ,\tau _n\}) = \min \nolimits _{1 \le i \le n} T\mathcal {B} (\tau _i)$$ and

$$\mathtt {Incr}(\{\mathtt {a}_1,\mathtt {a}_2,\ldots ,\mathtt {a}_n\}) = \sum \nolimits _{1 \le i \le n}\mathtt {Incr}(\mathtt {a}_i)$$ with $$\mathtt {Incr}(\emptyset ) = 0$$

*Discussion* If $atm _2(\kappa ) = \emptyset$ for all reset chains $\kappa$, Definition 23 is equal to Definition 21. This is the case for all $DCP$s with a reset forest (all examples in this article except Fig. 5). Definition 23 thus is a generalization of Definition 21.

**Table 6:**
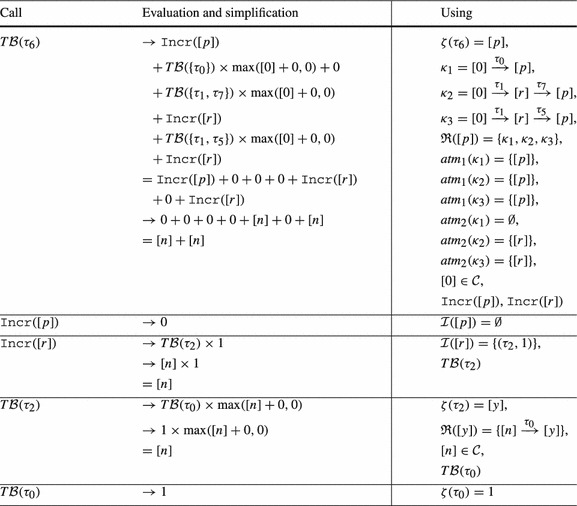
Computation of $T\mathcal {B} (\tau _6)$ for Fig. 5 by Definition 23

*Example* As shown in Table 6 we get $T\mathcal {B} (\tau _6) = [n] + [n]$ for Fig. 5 by Definition 23. I.e., we get the precise bound 2*n* for the loop at $l_4$ in Fig. 5 (*n* has type *unsigned*).

##### Theorem 2

( Soundness of Bound Algorithm using Reset Chains) Let ${\varDelta }\mathcal {P}(L, E, l_b, l_e)$ be a well-defined and fan-in free $DCP$ over atoms $\mathcal {A}$. Let $\zeta : E\mapsto Expr (\mathcal {A})$ be a *local bound mapping* for ${\varDelta }\mathcal {P}$. Let $T\mathcal {B}$ and $V\mathcal {B}$ be defined as in Definition 23. Let $\tau \in E$ and $\mathtt {a}\in \mathcal {A}$. If ${\varDelta }\mathcal {P}$ has a reset DAG then (1) $\llbracket T\mathcal {B} (\tau )\rrbracket$ is a *transition bound* for $\tau$ and (2) $\llbracket V\mathcal {B} (\mathtt {a})\rrbracket$ is a *variable bound* for $\mathtt {a}$.

##### Proof

See Electronic Supplementary Material.

*Complexity* A DAG can have exponentially many paths in the number of nodes. Thus there can be exponentially many reset chains in $\mathfrak {R}(\mathtt {v})$ (exponential in the number of variables and constants of the abstract program, i.e., the norms generated during the abstraction process, see Sect. 6). However, in our experiments enumeration of (optimal) reset chains did not affect performance. (See also our discussion on *scalability* in Sect. 10.1.)

### Preprocessing: Transforming a Reset Graph into a Reset DAG

**Fig. 6:**
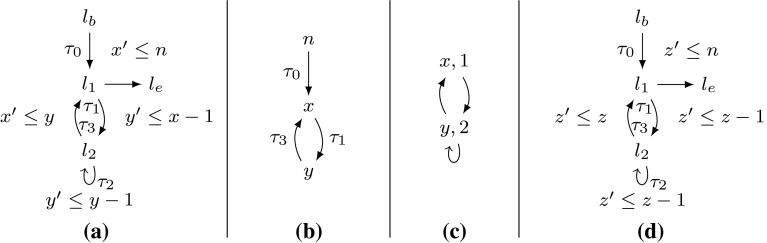
**a** Example, **b** Reset Graph, **c** Variable Flow Graph, **d** Variables Renamed

Consider the $DCP$ shown in Fig. 6a. Figure 6a has a cyclic reset graph as shown in Fig. 6b. In the following we describe an algorithm which transforms Fig. 6a into d by renaming the program variables. Figure 6d has an acyclic reset graph (a reset DAG).

#### Definition 24

(*Variable Flow Graph*) Let ${\varDelta }\mathcal {P}(L,E,l_b,l_e)$ be a $DCP$ over $\mathcal {A}$. We call the graph with node set $\mathcal {V}\times L$ and edge set

$$\begin{aligned} \{(y,l_1)\rightarrow (x,l_2) \mid {l_1 \xrightarrow {u} l_2 \in E\wedge x^\prime \le y + \mathtt {c}\in u\text { with } x, y \in \mathcal {V}}\} \end{aligned}$$

the *variable flow graph*.

For an example see Fig. 6c.

Let ${\varDelta }\mathcal {P}(L,E,l_b,l_e)$ be a $DCP$. Let $\{\mathtt {SCC}_1,\mathtt {SCC}_2,\ldots ,\mathtt {SCC}_n\}$ be the *strongly connected components* of its *variable flow graph*. For each SCC $\mathtt {SCC}_i$ we choose a fresh variable $\mathtt {v}_i \in \mathcal {V}$. Let $\varsigma : \mathcal {V}\times L\mapsto \mathcal {V}$ be the mapping $\varsigma (\mathtt {v}, l) = \mathtt {v}_i$, where *i* s.t. $(\mathtt {v}, l) \in \mathtt {SCC}_i$. We extend $\varsigma$ to $\mathcal {A}\times L\mapsto \mathcal {A}$ by defining $\varsigma (\mathtt {s},l) = \mathtt {s}$ for all $l\in L$ and $\mathtt {s}\in \mathcal {C}$.

We obtain ${\varDelta }\mathcal {P}^\prime (L,E^\prime ,l_b,l_e)$ from ${\varDelta }\mathcal {P}$ by setting $E^\prime = \{l_1 \xrightarrow {u^\prime } l_2 \mid l_1 \xrightarrow {u} l_2 \in E\}$, where $u^\prime$ is obtained from $u$ by generating the constraint $\varsigma (x,l_2)^\prime \le \varsigma (y,l_1) + \mathtt {c}$ from a constraint $x^\prime \le y + \mathtt {c}\in u$.

*Examples* Figure 6d is obtained from Fig. 6a by applying the described transformation using the mapping $\varsigma (x,l_1) = \varsigma (y,l_2) = z$.

*Soundness* Soundness of the described variable renaming is obvious if there are no two (different) variables $\mathtt {v}_1$ and $\mathtt {v}_2$ that are renamed to the same fresh variable at some location $l$. This is the case if in each SCC of the *variable flow graph* each location $l\in L$ appears at most once, i.e., if there is no SCC $\mathtt {SCC}$ in the *variable flow graph* of the program such that there is a location $l\in L$ and variables $\mathtt {v}_1,\mathtt {v}_2 \in \mathcal {V}$ with $\mathtt {v}_1 \ne \mathtt {v}_2$ and $(l,\mathtt {v}_1) \in \mathtt {SCC}$ and $(l,\mathtt {v}_2) \in \mathtt {SCC}$. In the literature, a program with this property is called *stratifiable* (e.g., [5]). A *fan-in free* $DCP$ that is *not stratifiable* can be transformed into a *stratifiable* and *fan-in free* $DCP$ by introducing appropriate case distinctions into the control flow of the program. Details are given in [30]. In the worst-case, however, this transformation can cause an exponential blow up of the number of transitions in the program (the size of the control flow graph).

## Finding Local Bounds

In this section we describe our algorithm for finding local bounds.

*Intuition* Let $\tau = {l_1 \xrightarrow {u} l_2} \in E$ and $\mathtt {v}\in \mathcal {V}$. Clearly, $\mathtt {v}$ is a *local bound* for $\tau$ if $\mathtt {v}$ decreases when executing $\tau$, i.e., if ${\mathtt {v}^\prime \le \mathtt {v}+ \mathtt {c}} \in u$ for some $\mathtt {c}< 0$. Moreover, $\mathtt {v}$ is a *local bound* for $\tau$, if every time $\tau$ is executed also some other transition $t \in E$ is executed and $\mathtt {v}$ is a local bound for $t$. This is, e.g., the case if $t$ is always executed either before each execution of $\tau$ or after each execution of $\tau$.

*Algorithm* The above intuition can be turned into a simple three-step algorithm. Let ${\varDelta }\mathcal {P}(L,E,l_b,l_e)$ be a $DCP$. (1) We set $\zeta (\tau ) = 1$ for all transitions $\tau$ that do not belong to a *strongly connected component* (SCC) of ${\varDelta }\mathcal {P}$. (2) Let $\mathtt {v}\in \mathcal {V}$. We define ${\xi }(\mathtt {v}) \subseteq E$ to be the set of all transitions $\tau = {l_1 \xrightarrow {u} l_2} \in E$ such that ${\mathtt {v}^\prime \le \mathtt {v}+ \mathtt {c}} \in u$ for some $\mathtt {c}< 0$. For all $\tau \in {\xi }(\mathtt {v})$ we set $\zeta (\tau ) = \mathtt {v}$. (3) Let $\mathtt {v}\in \mathcal {V}$ and $\tau \in E$. Assume $\tau$ was not yet assigned a local bound by (1) or (2). We set $\zeta (\tau ) = \mathtt {v}$ if $\tau$ does not belong to a *strongly connected component* (SCC) of the directed graph $(L,E^\prime )$ where $E^\prime = E{\setminus }\{{\xi }(\mathtt {v})\}$ (the control flow graph of ${\varDelta }\mathcal {P}$ without the transitions in ${\xi }(\mathtt {v})$).

If there are $\mathtt {v}_1 \ne \mathtt {v}_2$ s.t. $\tau \in {\xi }(\mathtt {v}_1) \cap {\xi }(\mathtt {v}_2)$ then $\zeta (\tau )$ is assigned either $\mathtt {v}_1$ or $\mathtt {v}_2$ non-deterministically. An alternative way of handling this case is as follows: we generate two local bound mappings, $\zeta _1$ and $\zeta _2$ where $\zeta _1(\tau ) = \mathtt {v}_1$ and $\zeta _2(\tau ) = \mathtt {v}_2$. This way we can systematically enumerate all possible choices, finally we apply our bound algorithm once based on $\zeta _1$, based on $\zeta _2$, etc., and finally take the minimum over all computed bounds. In our implementation, however, we follow the aforementioned greedy approach based on non-deterministic choice.

*Discussion on Soundness* Soundness of Steps (1) and (2) is obvious. We discuss soundness of Step (3): let $\tau \in E$. If $\tau$ does not belong to an SCC of $(L,E{\setminus }\{{\xi }(\mathtt {v})\})$ we have that some transition in ${\xi }(\mathtt {v})$ (which decreases $\mathtt {v}$) has to be executed in between any two executions of $\tau$. It remains to ensure that there is a decrease of $\mathtt {v}$ also for the last execution of $\tau$: for special cases this is unfortunately not the case. Consider Fig. 8b (Sect. 5). The above stated algorithm sets $\zeta (\tau _1) = [x]$. However, $[x]$ is not a *local bound* for $\tau _1$ of Fig. 8b because there is no decrease of $[x]$ for the last execution of $\tau _1$ (before executing $\tau _3$).

It is straightforward to ensure soundness of the algorithm: adding an edge from $l_e$ to $l_b$ forces the algorithm to take the last execution of a transition into account. I.e., we set $E^\prime = E\cup \{l_e \xrightarrow {\emptyset } l_b\}{\setminus }\{{\xi }(\mathtt {v})\}$. Now our algorithm fails to find a local bound for $\tau _1$ of Fig. 8b, which is sound. We discuss how we handle the example in Fig. 8 in Sect. 4.1.

*Complexity* Steps (1) and (2): can be implemented in linear time. Step (3): for each $\mathtt {v}\in \mathcal {V}$ we need to compute the SCCs of $(L,E{\setminus }{\xi }(\mathtt {v}))$. It is well known that SCCs can be computed in linear time (linear in the number of edges and nodes). Since we need to perform one SCC computation per variable, Step (3) is quadratic.

### Generalizing Local Bounds to Sets of Local Bounds

**Fig. 7:**
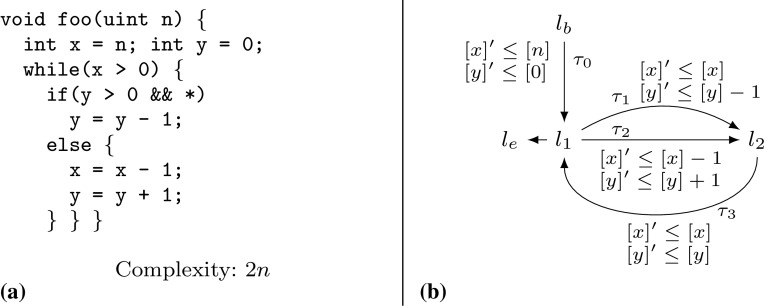
**a** Example, **b** $DCP$ obtained by abstraction

Consider the example in Fig. 7. In Fig. 7b the $DCP$ obtained by abstraction (Sect. 6) from the program in Fig. 7a is shown. We have that *x* is a local bound for $\tau _1$ and *y* is a local bound for $\tau _2$. However, it is not straightforward to find a local bound for $\tau _3$: in order to form a local bound for $\tau _3$ we need to combine *x* and *y* to a linear combination, e.g., $2x + y$. It is unclear how to automatically come up with such expressions.

In the following we discuss a simple generalization of our algorithm by which we avoid an explicit composition of local bounds.

We generalize the local bound mapping $\zeta : E\rightarrow Expr (\mathcal {A})$ (Definition 17) to a *local bound set mapping* $\zeta : E\rightarrow 2^{ Expr (\mathcal {A})}$.

#### Definition 25

(*Local Bound Set Mapping*) Let ${\varDelta }\mathcal {P}(L, E,l_b, l_e)$ be a $DCP$ over $\mathcal {A}$. Let $\rho = (l_b,\sigma _0) \xrightarrow {u_0} (l_1,\sigma _1) \xrightarrow {u_1} \cdots$ be a run of ${\varDelta }\mathcal {P}$. We call a function $\zeta : E\rightarrow 2^{ Expr (\mathcal {A})}$ a *local bound set mapping* for $\rho$ if for all $\tau \in E$ it holds that

$$\sharp (\tau ,\rho ) \le (\sum \nolimits _{\mathtt {v}\in \zeta (\tau ) \cap \mathcal {V}} {\downarrow }(\mathtt {v},\rho )) + \sum \nolimits _{\mathtt {expr}\in \zeta (\tau ){\setminus }\mathcal {V}} \llbracket \mathtt {expr}\rrbracket (\sigma _0)$$.

We say that $\zeta$ *is a local bound set mapping* for ${\varDelta }\mathcal {P}$ if $\zeta$ is a local bound set mapping for all runs of ${\varDelta }\mathcal {P}$.

*Example* For Fig. 7b we have that $\zeta : E\rightarrow 2^{ Expr (\mathcal {A})}$ with $\zeta (\tau _0) = \{1\}$, $\zeta (\tau _1) = \{[y]\}$, $\zeta (\tau _2) = \{[x]\}$ and $\zeta (\tau _3) = \{[x],[y]\}$ is a *local bound set mapping*.

We generalize the transition bound algorithm $T\mathcal {B}$ to local bound set mappings by summing up over all $\mathtt {expr}\in \zeta (\tau )$. We exemplify the generalization by extending Definition 19.

#### Definition 26

(*Bound Algorithm based on Local Bound Sets*) Let ${\varDelta }\mathcal {P}(L, E, l_b, l_e)$ be a $DCP$ over $\mathcal {A}$. Let $\zeta : E\rightarrow 2^{ Expr (\mathcal {A})}$. Let $V\mathcal {B} : \mathcal {A}\mapsto Expr (\mathcal {A})$ be defined as in Definition 19. We define $T\mathcal {B} : E\mapsto Expr (\mathcal {A})$ as:

$$\begin{aligned} T\mathcal {B} (\tau )&= \sum \limits _{\mathtt {lb}\in \zeta (\tau )} T\mathcal {B} (\mathtt {lb})\\ T\mathcal {B} (\mathtt {lb})&= \mathtt {lb}, \text { if } \mathtt {lb}\not \in \mathcal {V}, \text { else}\\ T\mathcal {B} (\mathtt {lb})&= \mathtt {Incr}(\mathtt {lb}) + \sum \limits _{( t ,\mathtt {a},\mathtt {c}) \in \mathcal {R}(\mathtt {lb})} T\mathcal {B} ( t ) \times \max ( V\mathcal {B} (\mathtt {a}) + \mathtt {c}, 0) \end{aligned}$$

where $\mathtt {Incr}(\mathtt {v}) = \sum \nolimits _{(\tau ,\mathtt {c}) \in \mathcal {I}(\mathtt {v})}{ T\mathcal {B} (\tau ) \times \mathtt {c}}$ (we set $\mathtt {Incr}(\mathtt {v}) = 0$ for $\mathcal {I}(\mathtt {v}) = \emptyset$).

*Example* For Fig. 7 we get $T\mathcal {B} (\tau _3) = 2n$, details are shown Table 7 ($[n] = n$ because *n* has type *unsigned*).

**Table 7:**
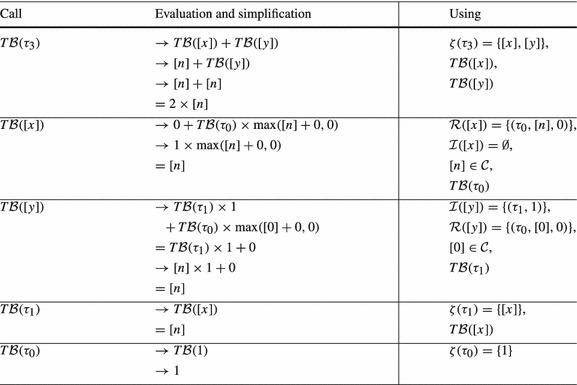
Computation of $T\mathcal {B} (\tau _3)$ for Fig. 7b by Definition 26

*Inferring a Local Bound Set Mapping* The algorithm for *finding local bounds* can be easily extended for finding *local bound sets*: steps (1) and (2) remain unchanged. Step (3) is generalized as follows: let $\mathtt {v}_1,\ldots ,\mathtt {v}_k \in \mathcal {V}$ and $\tau = l_1 \xrightarrow {u} l_2 \in E$. We set $\zeta (\tau ) = \{\mathtt {v}_1,\ldots ,\mathtt {v}_k\}$ if it holds that for each execution of $\tau$ a transition in ${\xi }(\mathtt {v}_1) \cup \cdots \cup {\xi }(\mathtt {v}_k)$ is executed. This can be implemented by checking, if $\tau$ does not belong to a *strongly connected component* (SCC) $\mathtt {SCC}$ of the directed graph $(L,E^\prime )$ where $E^\prime = E\cup \{l_e \xrightarrow {\emptyset } l_b\}{\setminus }({\xi }(\mathtt {v}_1) \cup \cdots \cup {\xi }(\mathtt {v}_k))$.

Note that Step (3) is parametrized in the number $k \in \mathbb {N}$ of variables considered. For obvious reasons it is preferable to find local bound sets of *minimal size*. Given a transition $\tau$, we therefore first try to find a local bound set of size $k=1$ for $\tau$ and increment *k* only if the search fails. With a fixed limit for *k* the complexity of our procedure for finding local bounds remains polynomial. To our experience limiting *k* to 3 is sufficient in practice.

**Fig. 8:**
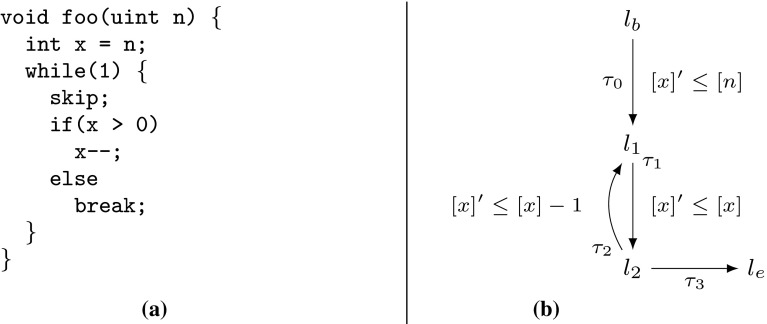
**a** Example with a “break”-statement, **b** $DCP$ obtained by abstraction

*Handling break statements* Consider Fig. 8a. The loop (resp. its back-edge) can be executed *n* times, the *skip* instruction (a placeholder for some code of interest), however, can be executed $n+1$ times. Consider the abstraction shown in Fig. 8b. Our algorithm for finding local bounds, as we discussed it so far, fails to find a local bound (set) for $\tau _1$ (modeling the *skip* instruction). We extend the algorithm as follows: we set ${\xi }(1) = \{\tau \in E\mid \tau \text { is not part of any SCC}\}$. I.e., for Fig. 8b we set ${\xi }(1) = \{\tau _0,\tau _3\}$. We add $1 \in Expr (\mathcal {A})$ to the set of “variables” $\mathtt {v}_1,\ldots ,\mathtt {v}_k$. I.e., for our example we have $\mathtt {v}_1 = [x]$ and $\mathtt {v}_2 = 1$. The algorithm now computes $\zeta (\tau _3) = \{[x], 1\}$ for $k = 2$ given that ${\xi }([x]) = \{\tau _2\}$. Based on $\zeta (\tau _3) = \{[x], 1\}$ our algorithm from Definition 26 correctly infers $T\mathcal {B} (\tau _3) = [n] + 1 = n + 1$ (*n* has type *unsigned*).

## Combined Bound Algorithm

We have developed our algorithm for computing transition bounds and variable bounds on $DCPs$ step-wise in Sect. 3 (Definitions 19, 21, 23), in each step adding new features to the algorithm. In this sense Definition 23 subsumes Definitions 21 and 19. We now combine Definition 23 with the extension to *sets of local bounds* (Sect. 4.1) and obtain Definition 27.

### Definition 27

(*Combined Bound Algorithm*) Let ${\varDelta }\mathcal {P}(L, E, l_b, l_e)$ be a $DCP$ over $\mathcal {A}$. Let $\zeta : E\rightarrow 2^{ Expr (\mathcal {A})}$. Let $V\mathcal {B} : \mathcal {A}\mapsto Expr (\mathcal {A})$ be defined as in Definition 19. We override the definition of $T\mathcal {B} : E\mapsto Expr (\mathcal {A})$ in Definition 19 by stating:

$$\begin{aligned} T\mathcal {B} (\tau )&= \sum \limits _{\mathtt {lb}\in \zeta (\tau )} T\mathcal {B} (\mathtt {lb})\\ T\mathcal {B} (\mathtt {lb})&= \mathtt {lb}, \text { if } \mathtt {lb}\not \in \mathcal {V}, \text { else}\\ T\mathcal {B} (\mathtt {lb})&= \mathtt {Incr}\left( \bigcup \limits _{\kappa \in \mathfrak {R}(\mathtt {lb})} atm _1(\kappa )\right) \\&\quad +\sum \limits _{\kappa \in \mathfrak {R}(\mathtt {lb})} T\mathcal {B} ( trn (\kappa )) \times \max ( V\mathcal {B} ( in (\kappa )) + c (\kappa ), 0) + \mathtt {Incr}( atm _2(\kappa )) \end{aligned}$$

where $$ T\mathcal {B} (\{\tau _1,\tau _2,\ldots ,\tau _n\}) = \min \nolimits _{1 \le i \le n} T\mathcal {B} (\tau _i)$$ and

$\mathtt {Incr}(\{\mathtt {a}_1,\mathtt {a}_2,\ldots ,\mathtt {a}_n\}) = \sum \nolimits _{1 \le i \le n}\mathtt {Incr}(\mathtt {a}_i)$ (we set $\mathtt {Incr}(\emptyset ) = 0$) and

$\mathtt {Incr}(\mathtt {v}) = \sum \nolimits _{(\tau ,\mathtt {c}) \in \mathcal {I}(\mathtt {v})}{ T\mathcal {B} (\tau ) \times \mathtt {c}}$ (we set $\mathtt {Incr}(\mathtt {v}) = 0$ for $\mathcal {I}(\mathtt {v}) = \emptyset$).

We introduced and discussed the terms from which Definition 27 is composed in Sects. 3 and 4.1.

*Soundness* Soundness of Definition 27 results from Theorem 2 (proven in Appendix and the discussion in Sect. 4.1. Note that Definition 27 is only sound for $DCP$s that have a *reset DAG*. We have described in Sect. 3.4 how to transform a given $DCP$ into a $DCP$ with a reset DAG.

## Program Abstraction

In the following we discuss how we abstract a given program to a $DCP$.

### Definition 28

(*Difference Constraint Invariants*) Let $\mathcal {P}(L,T,l_e,l_b)$ be a *program* over states ${\varSigma }$. Let $e_1,e_2,e_3$ be norms, i.e., $e_1,e_2,e_3: {{\varSigma }\rightarrow \mathbb {Z}}$, and let $c \in \mathbb {Z}$ be some integer. We say $e_1^\prime \le e_2 + e_3$ is *invariant on* a transition ${l_1 \xrightarrow {\lambda } l_2} \in T$, if $e_1(\sigma _2) \le e_2(\sigma _1) + e_3(\sigma _1)$ holds for all $(\sigma _1, \sigma _2) \in \lambda$.

### Definition 29

(*DCP Abstraction of a Program*) Let $\mathcal {P}= (L, T, l_b, l_e)$ be a program and let $N$ be a set of functions from the states to the natural numbers, i.e., $N \in 2^{{\varSigma }\rightarrow \mathbb {N}}$. A $DCP$ ${\varDelta }\mathcal {P}= (L, E, l_b, l_e)$ over atoms $N$ is an abstraction of the program $\mathcal {P}$ iff for each transition ${l_1 \xrightarrow {\lambda } l_2} \in T$ there is a transition ${l_1 \xrightarrow {u} l_2} \in E$ s.t. every ${e_1^\prime \le e_2 + c} \in u$ is invariant on ${l_1 \xrightarrow {\lambda } l_2}$.

Our abstraction algorithm proceeds in two steps: we first abstract a given concrete program to a $DCP$ with integer semantics, in a second step we then further abstract the integer-$DCP$ to a $DCP$ over the natural numbers (as defined in Definition 12).

### Abstraction I: $DCP$s with Integer Semantics

We extend our abstract program model from Definition 12 to the non-well-founded domain $\mathbb {Z}$ by adding guards to the transitions of the program.

*Syntax of DCP s with guards* The edges $E$ of a *DCP with guards* ${\varDelta }\mathcal {P}_G(L, E,l_b, l_e)$ are a subset of $L\times 2^{\mathcal {V}} \times 2^{\mathcal {DC}(\mathcal {A})} \times L$. I.e., an edge of a *DCP with guards* is of form $l_1 \xrightarrow {g,u} l_2$ with $l_1,l_2 \in L$, $g\in 2^\mathcal {V}$ and $u\in 2^{\mathcal {DC}(\mathcal {A})}$.

*Example* See Fig. 10a in Sect. 7.1 for an example.

*Semantics of DCP s with guards* We extend the range of the *valuations* $Val _\mathcal {A}$ of $\mathcal {A}$ from $\mathbb {N}$ to $\mathbb {Z}$. Let $u\in 2^{\mathcal {DC}(\mathcal {A})}$. Let $\llbracket u\rrbracket$ be as defined in Definition 13. Let $g\in 2^\mathcal {V}$. We define $\llbracket g, u\rrbracket = \{{(\sigma _1,\sigma _2) \in \llbracket u\rrbracket } \mid {\sigma _1(\mathtt {v}) > 0} \text { for all } \mathtt {v}\in g\}$. A *guarded* $DCP$ ${\varDelta }\mathcal {P}_G= (L, E, l_b,l_e)$ is a *program* over the set of states $Val _\mathcal {A}$ with locations $L$, entry location $l_b$, exit location $l_e$ and transitions $T= \{l_1 \xrightarrow {\llbracket g, u\rrbracket } l_2 \mid l_1 \xrightarrow {g, u} l_2 \in E\}$.

I.e., a transition $l_1 \xrightarrow {g,u} l_2$ of a *DCP with guards* can only be executed if the values of all $\mathtt {v}\in g$ are greater than 0.

#### Definition 30

(*Guard*) Let $\mathcal {P}(L,T,l_e,l_b)$ be a *program* over states ${\varSigma }$. Let $e$ be a norm ($e: {\varSigma }\rightarrow \mathbb {Z}$), let $c \in \mathbb {Z}$. We say $e$ is a *guard* of ${l_1 \xrightarrow {\lambda } l_2} \in T$ if $e(\sigma _1) > 0$ holds for all $(\sigma _1, \sigma _2) \in \lambda$.

We abstract a program $\mathcal {P}= (L, T,l_b,l_e)$ to a $DCP$ with guards ${\varDelta }\mathcal {P}_G= (L, E,l_b,l_e)$ as follows:

1. *Choosing an initial set of Norms* We aim at creating a suitable abstract program for bound analysis. In our non-recursive setting complexity evolves from iterating loops. Therefore we search for expressions which limit the number of loop iterations. We consider conditions of form $a > b$ resp. $a \ge b$ found in loop headers or on loop-paths if they involve loop counter variables, i.e., variables which are incremented and/or decremented inside the loop. Such conditions are likely to limit the consecutive execution of single or multiple loop-paths. From each condition of form $a > b$ we create the integer expression $a - b$, from each condition of form $a \ge b$ we create the integer expression $a + 1 - b$. These expressions form our initial set of norms $N$. Note that on those transitions on which $a > b$ holds, $a - b > 0$ must hold, whereas with $a \ge b$ we have $a + 1 - b > 0$.
  In ${\varDelta }\mathcal {P}_G$ we interpret a norm $e\in N$ from our initial set of norms $N$ as *variable*, i.e., we have $e\in \mathcal {V}$ for all $e\in N$.
2. *Abstracting Transitions* For each transition ${l_1 \xrightarrow {\lambda } l_2} \in T$ we generate a set $u_\lambda$ of difference constraints: initially we set $u_\lambda = \emptyset$ for all transitions ${l_1 \xrightarrow {\lambda } l_2} \in T$.
  We repeat the following construction *until* the set of norms $N$ becomes *stable*: For each $e_1 \in N$ and for each ${{l_1 \xrightarrow {\lambda } l_2}} \in T$, such that all variables in $e_1$ are defined at $l_2$, we check whether there is a difference constraint of form $e_1^\prime \le e_2 + \mathtt {c}$ with $e_2\in N$ and $\mathtt {c}\in \mathbb {Z}$ in $u_\lambda$. If not, we derive a difference constraint ${e_1^\prime \le e_2 + \mathtt {c}}$ as follows: we symbolically execute $\lambda$ for deriving $e_1^\prime$ from $e_1$: e.g., let $e_1 = x + y$ and assume *x* is assigned $x + 1$ on ${l_1 \xrightarrow {\lambda } l_2}$ while *y* stays unchanged. We get $e_1^\prime = x + 1 + y$ through symbolic execution. In order to keep the number of norms low, we first try
  - to find a norm $e_2 \in N$ and $\mathtt {c}\in \mathbb {Z}$ s.t. $e_1^\prime \le e_2 + \mathtt {c}$ is invariant on $l_1 \xrightarrow {\lambda } l_2$ (see Definition 28). If we succeed we add the predicate $e_1^\prime \le e_2 + \mathtt {c}$ to $u_\lambda$. E.g., for $e_1 = x + y$ and $e_1^\prime = x + 1 + y$ we get the transition invariant $(x + y)^\prime \le (x + y) + 1$ and will thus add $e_1^\prime \le e_1 + 1$ to $u_\lambda$. In general, we find a norm $e_2$ and a constant $\mathtt {c}$ by separating constant parts in the expression $e_1^\prime$ using associativity and commutativity, thereby forming an expression $e_3$ over variables and program parameters and an integer constant $\mathtt {c}$. E.g., given $e_1^\prime = 5 + z$ we set $e_3 = z$ and $\mathtt {c}= 5$. We then search a norm $e_2 \in N$ with $e_2 = e_3$ where the check on equality is performed modulo associativity and commutativity.
  - If (a) fails, i.e., no such $e_2 \in N$ exists, we add $e_3$ to $N$ and derive the predicate $e_1^\prime \le e_3 + \mathtt {c}$. In ${\varDelta }\mathcal {P}_G$ we interpret $e_3$ as atom, i.e., $e_3 \in \mathcal {A}$. We interpret $e_3$ as a symbolic constant, i.e., $e_3 \in \mathcal {C}$, only if $e_3$ is purely built over the program’s input parameters and constants. Note that this step increases the number of norms.
3. *Inferring Guards* For each transition ${l_1 \xrightarrow {\lambda } l_2}$ we generate a set $g_\lambda$ of guards: initially we set $g_\lambda = \emptyset$ for all transitions ${l_1 \xrightarrow {\lambda } l_2}$. For each $e\in N$ and each transition ${l_1 \xrightarrow {\lambda } l_2}$ we check if $e$ is a *guard* of ${l_1 \xrightarrow {\lambda } l_2}$. If so, we add $e$ to $g_\lambda$. We use an SMT solver to perform this check. E.g., let $e= x + y$ and assume that ${l_1 \xrightarrow {\lambda } l_2}$ is guarded by the conditions $x \ge 0$ and $y > x$. An SMT solver supporting *linear arithmetic* proves that $x \ge 0 \wedge y > x$ implies $x + y$
4. We set $$E= \{{l_1 \xrightarrow {g_\lambda ,u_\lambda } l_2} \mid {l_1 \xrightarrow {\lambda } l_2} \in T\}$$.

Note that SMT reasoning is applied only *locally* to single transitions to check if an expression is greater than 0 on that transition.

*Propagation of Guards* We improve the precision of our abstraction by *propagating guards*: consider a transition $l_3 \xrightarrow {g_3,u_3} l_4$. Assume $l_3$ has the incoming edges $l_1 \xrightarrow {g_1,u_1} l_3$ and $l_2 \xrightarrow {g_2,u_2} l_3$. If $y \in g_1 \cap g_2$ (i.e., *y* is a guard on both incoming edges) *and* *y* does not decrease on the corresponding concrete transitions $l_1 \xrightarrow {\lambda _1} l_3$ and $l_1 \xrightarrow {\lambda _2} l_3$ (checked by symbolic execution) then *y* is also a guard on $l_3 \xrightarrow {g_3,u_3} l_4$ and we add *y* to $g_3$.

*Well-defined and Fan-in free* $DCP$s generated by our algorithm are always *fan-in free* by construction: for each transition we get at most one predicate ${e}^\prime \le e_2 + \mathtt {c}$ for each $e\in N$ because we check whether there is already a predicate for $e$ before a predicate is inferred resp. added. We ensure *well-definedness* of our abstraction by a final *clean-up*: we iterate over all $l\in L$ and check if $\mathtt {use}(l) \subseteq \mathtt {def}(l)$ holds. If this check fails we remove all difference constraints $x^\prime \le y + \mathtt {c}$ with $y \in \mathtt {use}(l){\setminus }\mathtt {def}(l)$ from all outgoing edges of $l$. We repeat this iteration until well-definedness is established, i.e., until $\mathtt {use}(l) \subseteq \mathtt {def}(l)$ holds for all $l\in L$.

*Termination* We have to ensure the termination of our abstraction procedure, since case (b) in step “*2. Abstracting Transitions*” triggers a recursive abstraction for the newly added norm: note that we can always stop the abstraction process at any point, getting a sound abstraction of the original program. We therefore ensure termination of the abstraction algorithm by limiting the chain of recursive abstraction steps that is triggered by entering case (2.b).

*Non-linear Iterations* We can handle counter updates such as $x^\prime = 2x$ or $x^\prime = x/2$ as follows: (1) We add the expression $\log x$ to our set of norms. (2) We derive the difference constraint $(\log x)^\prime \le (\log x) - 1$ from the update $x' = x / 2$ if $x > 1$ holds. Symmetrically we get $(\log x)^\prime \le (\log x) + 1$ from the update $x^\prime = 2x$ if $x > 0$ holds.

*Data Structures* In previous publications [13, 24] it has been described how to abstract programs with data structures to pure integer programs by making use of appropriate *norms* such as the length of a list or the number of elements in a tree. In our implementation we follow these approaches using a light-weight abstraction based on optimistic aliasing assumptions (see [30] for details). Once the program is transformed to an integer program, our abstraction algorithm is applied as described above for obtaining a difference constraint program.

### Abstraction II: From the Integers to the Natural Numbers

We now discuss how we abstract a *DCP with guards* ${\varDelta }\mathcal {P}_G= (L, E,l_b,l_e)$ to a $DCP$ ${\varDelta }\mathcal {P}= (L, E^\prime ,l_b,l_e)$ over $\mathbb {N}$ (Definition 12):

Let $e\in N$. By $[e]: {\varSigma }\rightarrow \mathbb {N}$ we denote the function $[e](\sigma ) = \max (e(\sigma ), 0)$. Recall that $e$ is interpreted as *atom* in ${\varDelta }\mathcal {P}_G$, i.e., $e\in \mathcal {A}$. In ${\varDelta }\mathcal {P}$, we interpret $[e]$ as *variable* (i.e., $[e] \in \mathcal {V}$) if $e\in \mathcal {V}$. We interpret $[e]$ as *symbolic constant* (i.e., $[e] \in \mathcal {C}$) if $e\in \mathcal {C}$.

Let $l_1 \xrightarrow {g,u} l_2 \in E$. We create a transition $l_1 \xrightarrow {u^\prime } l_2 \in E^\prime$ as follows: let $e_1^\prime \le e_2 + \mathtt {c}\in u$. If $\mathtt {c}\ge 0$, we add $[e_1]^\prime \le [e_2] + \mathtt {c}$ to $u^\prime$. If $\mathtt {c}< 0$ and $e_2 \in g$ we add the constraint $[e_1]^\prime \le [e_2] - 1$ to $u^\prime$. If $\mathtt {c}< 0$ and $e_2 \not \in g$ we add the constraint $[e_1]^\prime \le [e_2] + 0$ to $u^\prime$.

*Discussion* Soundness of Abstraction II is due to the following observation: consider a transition $l_1 \xrightarrow {g,u} l_2$ of ${\varDelta }\mathcal {P}_G$. Let $e_1^\prime \le e_2 + \mathtt {c}\in u$, i.e., $e_1^\prime \le e_2 + \mathtt {c}$ is *invariant* for the corresponding transition $\tau$ of the concrete program. Then $[e_1]^\prime \le [e_2] + 0$ is also invariant for $\tau$. Further: if $\mathtt {c}\ge 0$ then $[e_1]^\prime \le [e_2] + \mathtt {c}$ is invariant for $\tau$. And if $\mathtt {c}< 0$ and $e_2 \in g$ (i.e., $e_2 > 0$ must hold before executing $\tau$), then $[e_1]^\prime \le [e_2] - 1$ is invariant for $\tau$.

*Modeling arbitrary Decrements* Consider a transition $l_1 \xrightarrow {g,u} l_2$ of ${\varDelta }\mathcal {P}_G$. Assume $e_1 \in G$ and ${e_1^\prime \le e_1 - 2} \in u$. Our abstraction procedure, as discussed so far, adds $[e_1]^\prime \le [e_1] - 1$ to $u^\prime$, where $l_1 \xrightarrow {u^\prime } l_2$ is the corresponding transition of ${\varDelta }\mathcal {P}$. We discuss how we can model a decrease by 2 in ${\varDelta }\mathcal {P}$ (such decreases are handled by Improvement I in Sect. 3.2): let $\tau = l_1 \xrightarrow {\lambda } l_2$ denote the corresponding transition of the concrete program. We make the following observation: since $e_1$ is a *guard invariant* for $\tau$ ($e_1 > 0$ before executing $\tau$) and ${e_1^\prime \le e_1 - 2}$ is *invariant* for $\tau$ we have that $[e_1 + 1]^\prime \le [e_1 + 1] - 2$ is invariant for $\tau$. We thus add the predicate $[e_1 + 1]^\prime \le [e_1 + 1] - 2$ to $u^\prime$. Further: let $\tau ^\prime \ne \tau$ be some transition of the concrete program. If $[e_1]^\prime \le [e_1] + \mathtt {c}$ is invariant for $\tau ^\prime$ then $[e_1 + 1]^\prime \le [e_1 + 1] + \mathtt {c}$ is also invariant for $\tau ^\prime$. Let $e_1 \ne e_2$. If $[e_1]^\prime \le [e_2] + \mathtt {c}$ is invariant for $\tau ^\prime$ then $[e_1 + 1]^\prime \le [e_2] + (\mathtt {c}+ 1)$ is also invariant for $\tau ^\prime$. We add the corresponding predicates to ${\varDelta }\mathcal {P}$.

We can handle decrements greater than 2 accordingly: e.g., if ${e_1^\prime \le e_1 - 3} \in u$ we add the predicate $[e_1 + 2]^\prime \le [e_1 + 2] - 3$ to $u^\prime$, etc.

**Fig. 9:**
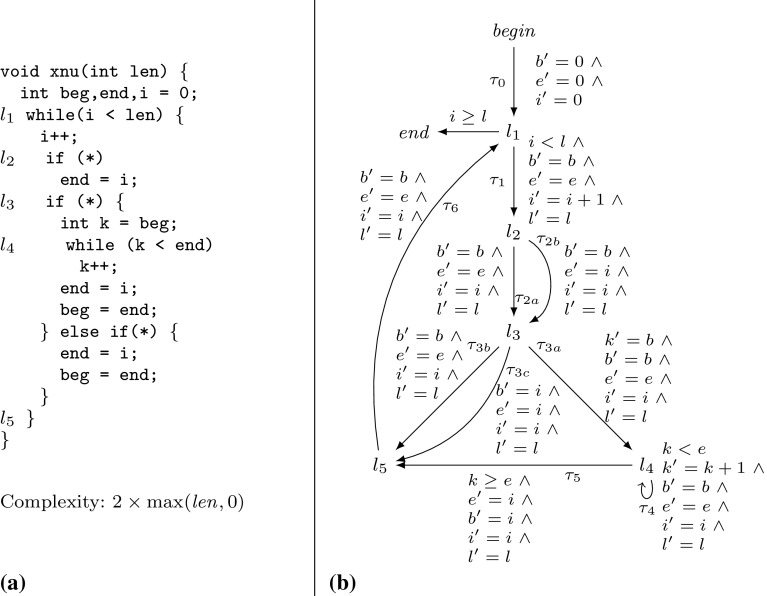
**a** Example xnu  **b** formal representation of xnu by an LTS

## A Complete Example

Example xnu in Fig. 9a contains a snippet of the source code after which we have modeled Example xnuSimple in Fig. 1. The full version of Example xnu can be found in the SPEC CPU2006 benchmark,^1^ in function XNU of 456.hmmer/src/masks.c. The outer loop in Example xnu partitions the interval [0, *len*] into disjoint sub-intervals [*beg*, *end*]. The inner loop iterates over the sub-intervals. Therefore the inner loop has an overall linear iteration count. Example xnu is a natural example for amortized complexity: Though a single visit to the inner loop can cost *len* (if $beg = 0$ and $end = len$), several visits can also not cost more than *len* since in each visit the loop iterates over a disjoint sub-interval. We therefore have: The *amortized cost* of a visit to the inner loop, i.e., the cost of executing the inner loop within an iteration of the outer loop averaged over all *len* iterations of the outer loop, is 1. Here, we refer by *cost* to the number of consecutive back jumps in the inner loop. But in general, any *resource consumption* inside the inner loop can, in total, only be repeated up to $\max ( len , 0)$ times.

Together with the loop bound $\max ( len , 0)$ of the outer loop, our observation yields an overall complexity of $2 \times \max ( len , 0)$.

Our experimental results (Sect. 8.3) demonstrate that state-of-the-art bound analyses fail to infer tight bounds for Example xnu and similar problems.

### Abstraction

We give a formal representation of the *concrete* program semantics of Example xnu in form of a *labeled transition system* (LTS) shown in Fig. 9b. Each edge in the LTS is labeled by a formula which encodes the transition relation. Consider, e.g., the edge from $l_1$ to $l_2$ ($\tau _1$) labeled by the formula ${i < l} \wedge {b^\prime = b} \wedge {e^\prime = e} \wedge {i^\prime = i + 1} \wedge {l^\prime = l}$. This formula induces the transition relation $\lambda _1 = \{(\sigma ,\sigma ^\prime ) \in {\varSigma }\times {\varSigma }\mid \sigma (i) < \sigma (l) \wedge \sigma ^\prime (b) = \sigma (b) \wedge \sigma ^\prime (e) = \sigma (e) \wedge \sigma ^\prime (i) = \sigma (i) + 1 \wedge \sigma ^\prime (l) = \sigma (l)\}$.

**Fig. 10:**
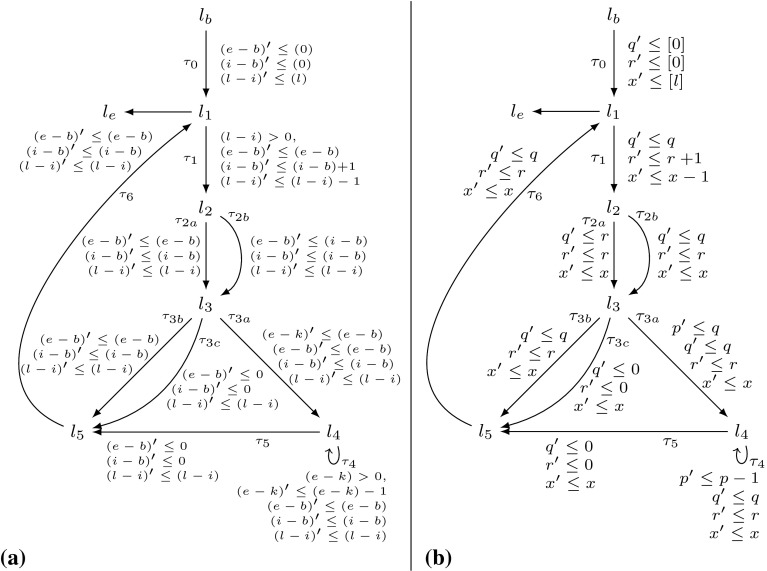
**a** Abstraction I: $DCP$ with guards for Example xnu . **b** Abstraction II: $DCP$ for Example xnu, assuming $p,q,r,x \in \mathcal {V}$ and $[e - k] = p$, $[e - b] = q$, $[i - b] = r$, $[l - i] = x$

We now discuss how our abstraction algorithm from Sect. 6 abstracts Example xnu to the $DCP$ shown in Fig. 10b. Recall abstraction Step I discussed in Sect. 6.1.

1. *Choosing an initial set of Norms* Our described heuristic adds the expressions $l - i$ and $e - k$ generated from the conditions $k < e$ and $i < l$ to the initial set of norms *N*. Thus our initial set of norms is $N = \{l - i, e - k\}$.
2. *Abstracting Transitions*
  We check how $l - i$ changes on the transitions $\tau _0$, $\tau _1$, $\tau _{2a}$, $\tau _{2b}$, $\tau _{3a}$,$\tau _{3b}$, $\tau _{3c}$, $\tau _4$, $\tau _5$, $\tau _6$:
$\tau _0$: we derive $[l - i]^\prime \le l$ (reset), we add *l* to *N*. Since *l* is an input parameter we have $l \in \mathcal {C}$.
$\tau _1$: we derive $[l - i]^\prime \le [l - i] - 1$ (decrement)
$$\tau _{2a},\tau _{2b},\tau _{3a},\tau _{3b},\tau _{3c},\tau _4,\tau _5,\tau _6$$: $$l - i$$ unchanged
  We check how $e - k$ changes on the transitions $\tau _{3a},\tau _4$ (*k* is only defined at $l_4$):
$\tau _{3a}$: we derive $[e -k]^\prime \le [e - b]$ (reset), we add $e - b$ to *N*
$\tau _4$: we derive $[e -k]^\prime \le [e - k] - 1$ (decrement)
  We check how $e - b$ changes on the transitions $\tau _0$, $\tau _1$, $\tau _{2a}$, $\tau _{2b}$, $\tau _{3a}$, $\tau _{3b}$, $\tau _{3c}$, $\tau _4$, $\tau _5$, $\tau _6$:
$\tau _0$: we derive $[e - b]^\prime \le 0$ (reset), we add 0 to *N*. Since 0 is a constant we have $0 \in \mathcal {C}$.
$\tau _{2a}$: we derive $[e - b]^\prime \le [i - b]$, we add $i - b$ to *N*.
$\tau _{3c}$: we derive $[e - b]^\prime \le 0$ (reset)
$\tau _5$: we derive $[e - b]^\prime \le 0$ (reset)
$$\tau _1,\tau _{2b},\tau _{3a},\tau _{3b},\tau _4,\tau _6$$: $$e - b$$ unchanged
  We check how $i - b$ changes on the transitions $\tau _0$, $\tau _1$, $\tau _{2a}$, $\tau _{2b}$, $\tau _{3a}$,$\tau _{3b}$, $\tau _{3c}$, $\tau _4$, $\tau _5$, $\tau _6$:
$\tau _0$: we derive $[i - b]^\prime \le 0$ (reset)
$\tau _1$: we derive $[i - b]^\prime \le [i - b] + 1$ (increment)
$\tau _{3c}$: we derive $[i - b]^\prime \le 0$ (reset)
$\tau _5$: we derive $[i - b]^\prime \le 0$ (reset)
$$\tau _{2a},\tau _{2b},\tau _{3a},\tau _{3b},\tau _4,\tau _6$$: unchanged
  We have processed all norms in *N*
3. *Inferring Guards* We add the guard $[l - i]$ to $\tau _1$ in ${\varDelta }\mathcal {P}_G$ because $l - i$ is a *guard* of $\tau _1$ in $\mathcal {P}$ (due to the condition $i < l$), we add the guard $[e - k]$ to $\tau _4$ in ${\varDelta }\mathcal {P}_G$ because $e - k$ is a *guard* of $\tau _4$ in $\mathcal {P}$ (due to the condition $k < e$).
4. The resulting $DCP$ with guards is shown in Fig. 10a.

**Fig. 11:**
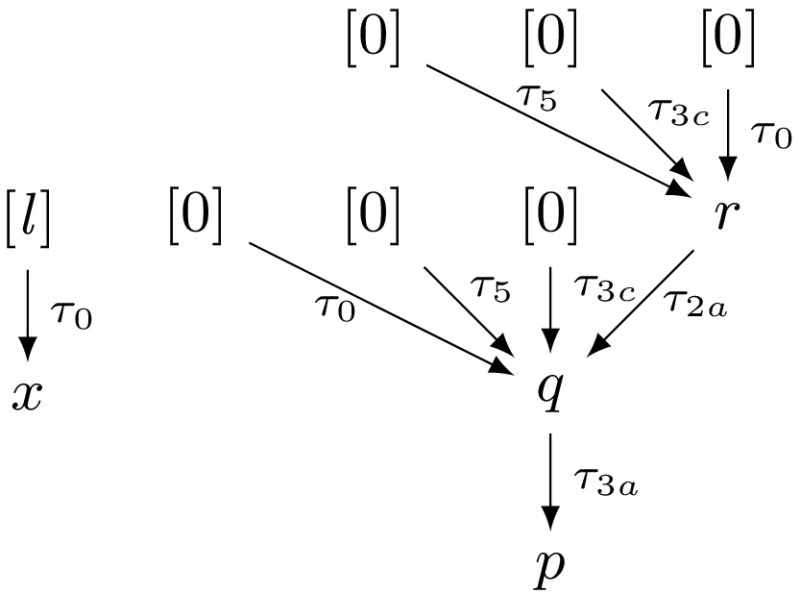
Reset graph

Applying abstraction Step II discussed in Sect. 6.2 gives us the $DCP$ shown in Fig. 10b. In the depiction of the abstraction we assume $p,q,r,x \in \mathcal {V}$ and $[e - k] = p$, $[e - b] = q$, $[i - b] = r$, $[l - i] = x$.

### Bound Computation

In Fig. 11 the reset graph of Fig. 10b is shown. Table 8 shows how our bound algorithm from Sect. 3 infers the *linear* bound $\max (l, 0)$ for the inner loop at $l_4$ of Example xnu by computing $T\mathcal {B} (\tau _4) = [l]$ on the abstraction shown in Fig. 10b. Recall that the abstract variable $[l]$ represents the expression $\max (l, 0)$ in the concrete program.

**Table 8:**
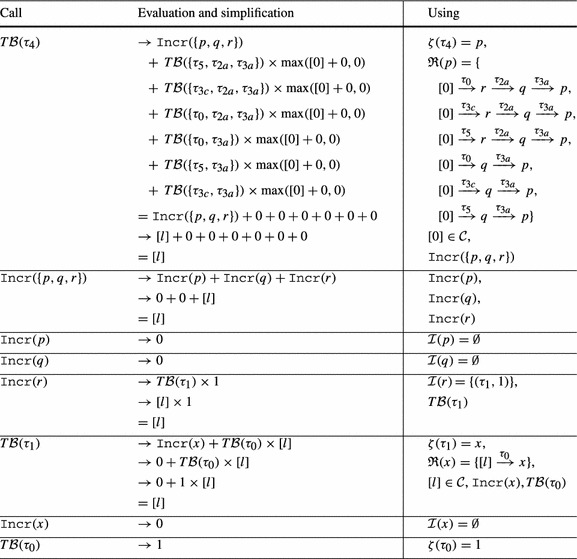
Computation of $T\mathcal {B} (\tau _4)$ for Example xnu (Fig. 9) by Definition 21 resp. Definition 23

Note that the $DCP$ in Fig. 10b has a *reset forest*. Therefore $atm _1(\kappa ) = atm (\kappa )$ for all reset paths $\kappa$ of Fig. 10b, as discussed in Sect. 3.3.2. The computation traces of Definitions 21 and 23 are thus equivalent for Fig. 10b.

## Experiments

*Implementation* The presented analysis defines the core ideas and techniques of our implementation loopus. A complete description of the implemented techniques, including a *path-sensitive* extension of our bound algorithm, is given in [30]. Our implementation is open-source and available at [18]. loopus reads in the LLVM [23] intermediate representation and performs an intra-procedural analysis. It is capable of computing bounds for loops as well as analyzing the complexity of non-recursive functions.

In the following we discuss three experimental setups and tool comparisons. Our first experiment, which we discuss in Sect. 8.1 is performed on a benchmark of open-source C programs. For our second experiment (Sect. 8.2), we assembled a benchmark of challenging programs from the literature on automatic bound analysis. The third experiment was performed on a set of interesting loop iteration patterns that we found in real source code.

### Evaluation on Real World C Code

*Experimental Setup* We base our experiment on the program and compiler optimization benchmark *Collective Benchmark* [17] (cBench), which contains a total of 1027 different C files (after removing code duplicates) with 211.892 lines of code. We set up the first comparison of complexity analysis tools on real-world code. For comparing our tool (loopus’15) we chose the three most promising tools from recent publications: the tool KoAT implementing the approach of [6], the tool CoFloCo implementing [10] and our own earlier implementation loopus’14 [29]. Note that we compared against the most recent versions of KoAT and CoFloCo (download 01/23/15).^2^ We were not able to evaluate Rank (implementing [2]) and C4B (implementing [7]) on our benchmark because both tools support only a limited subset of C. The experiments were performed on a Linux system with an Intel dual-core 3.2 GHz processor and 16 GB memory. The task was to perform a complexity analysis on function level. We used the following experimental set up:

1. We compiled all 1027 C files in the benchmark into the LLVM intermediate representation using clang.
2. We extracted all 1751 functions which contain at least one loop using the tool *llvm-extract* (comes with the LLVM tool suite). Extracting the functions to single files guarantees an intra-procedural setting for all tools.
3. We used the tool *llvm2kittel* [20] to translate the 1751 LLVM modules into 1751 text files in the integer transition system (ITS) format that is read in by KoAT.
4. We used the transformation described in [10] to translate the ITS format of KoAT into the cost equations representation that is read in by CoFloCo. This last step is necessary because there exists no direct way for translating C or the LLVM intermediate representation into the CoFloCo input format.
5. We decided to exclude the 91 recursive functions from the benchmark set because we were not able to run CoFloCo on these examples (the transformation tool does not support recursion), KoAT was not successful on any of them, and loopus does not support recursion. In total our example set thus comprises 1659 functions.

**Table 9:** Tool results on analyzing the complexity of 1659 functions in the cBench benchmark, none of the tools infers $log$ bounds

	Succ.	1	*n*	$n^2$	$n^3$	$n^{> 3}$	$2^n$	Total time	# Time outs
loopus’15	806	205	489	97	13	2	0	15 m	6
loopus’14	431	200	188	43	0	0	0	40 m	20
KoAT	430	253	138	35	2	0	2	5.6 h	161
CoFloCo	386	200	148	38	0	0	0	4.7 h	217

*Evaluation* Table 9 shows the results of all four tools on our benchmark using a time out of 60 s (Table 10 shows the results on the subset of those functions on which no tool timed out). The first column shows the number of functions which were successfully bounded by the respective tool, the last column shows the number of time outs, on the remaining examples (not shown in the table) the respective tool did not time out but was also not able to compute a bound. The column *Time* shows the total time used by the respective tool to process the benchmark. loopus’15 obtains results for about twice as many functions as KoAT, CoFloCo, and loopus’14 while needing an order of magnitude less time than KoAT and CoFloCo, and significantly less time than loopus’14. We conclude that our implementation is both more scalable and, on real C code, more successful than implementations of other state-of-the-art approaches. However, while the experiment clearly demonstrates that our implementation outperforms the competitors with respect to scalability, it does not allow to compare the strengths of the different bound analyses conclusively: we observed that llvm2kittel, the only tool available for translating C-code resp. the LLVM intermediate representation into the ITS format of KoAT, looses information that is kept by our analysis. As a result, it is unclear if a failure to compute a bound is due to different analysis strength or due to information loss during translation (we have not seen such an information loss for our second and third experiment, on which we report in Sects. 8.2 and 8.3, where the considered benchmarks consist of rather small, pure integer programs for which llvm2kittel works well).

**Table 10:** Tool results on analyzing the complexity of the subset of those functions in the cBench benchmark on which no tool timed out

	Succ.	1	*n*	$n^2$	$n^3$	$n^{> 3}$	$2^n$	Total time	# Time outs
loopus’15	753	196	466	84	7	0	0	9 m	0
loopus’14	414	192	181	41	0	0	0	20 m	0
KoAT	420	245	136	35	2	0	2	2.9 h	0
CoFloCo	382	198	146	38	0	0	0	1.1 h	0

We hope that our experiment motivates the development of better tools for bound analysis of real world-code and drives the research towards solving realistic complexity analysis problems. We want to add that to our experience, working with C programs instead of integer transition systems is very helpful for developing and debugging a complexity analysis tool: looking at C code, we can use our own intuition as programmers about the expected complexity of the analyzed code and compare it to the complexity reported by the tool.

*Pointers and Shapes* Even loopus’15 computed bounds for only about half of the functions in the benchmark. Studying the benchmark code we concluded that for many functions pointer alias and/or shape analysis is needed for inferring functional complexity. In our experimental comparison such information was not available to the tools. Using optimistic (but unsound) assumptions on pointer aliasing and heap layout, our tool loopus’15 was able to compute the complexity for in total 1185 out of the 1659 functions in the benchmark, using 28 min total time. A discussion of our optimistic pointer aliasing and heap layout assumption and on the reasons of failure can be found in [30].

The benchmark and more details on our experimental results can be found on [18] where our tool is also offered for download.

### Evaluation on Examples from the Literature

**Table 11:** Tool Results on analyzing examples from the literature, none of the tools infers $log$ bounds

	Succ.	1	*n*	$n^2$	$n^3$	$n^{> 3}$	$2^n$	Time w/o TO	# Time outs
loopus’15	86	2	51	27	1	5	0	4 s	0
loopus’14	86	2	50	28	2	4	0	4 s	0
CoFloCo	87	3	45	34	2	3	0	1 min 40 s	1
Rank	78	3	49	21	3	2	0	20 s	0
C4B	36	0	36	0	0	0	0	6 s	0
KoAT	90	3	43	36	3	5	0	3 min 50 s	3

The time out was 120 s. A higher time out did not yield additional results

In order to evaluate the precision of our approach on a number of known challenges to bound analysis, we performed a tool comparison on 110 examples from the literature. Our example set comprises those examples from the tool evaluation in [6, 29] that were available as imperative code (C or pseudo code, in total 89 examples), and additionally the examples used for the evaluation of Ref. [7] (15 examples) as well as the running examples of Ref. [27] (6 examples). We added the tools *Rank* (implementing [2]) and *C4B* (implementing [7]) to the comparison, because we were able to formulate the examples over the restricted C subset that is supported by these two tools (this was not possible for our experiment on real-world code).

The results of our evaluation are shown in Table 11. Our two tools loopus’15 and loopus’14 compute the highest number of *linear* bounds and are also significantly faster than the other tools, in particular than KoAT and CoFloCo. On the other hand, KoAT computes the highest number of bounds in total (4 more than loopus). CoFloCo computes, in total, 1 bound more than our tool. The comparable low number of bounds computed by C4B is also due to the fact that the approach implemented in C4B is limited to linear bounds.

In summary, our second evaluation shows that our approach is not only successful on the class of problems on which we focused in this article, but solves also many other bound analysis problems from the literature. Note, that in contrast to our first evaluation, our second benchmark contains small examples from academia (1293 LOC, in average 12 lines per file). On these examples our implementation is comparable in strength to the implementation of other state-of-the-art approaches to bound analysis. Given that our tool is a prototype implementation, there is room for improvement, concrete suggestions are discussed in [30]. More details on the results computed by each tool can be found on [19].

### Evaluation on Challenging Iteration Patterns from Real Code

Scanning through two C-code benchmarks (*cBench* [17] and *SPEC CPU 2006* [21]), we found a number of 23 *different* loop iteration patterns which we consider to be particular challenging for state-of-the-art bound analyses. The 23 patterns have the following property in common: (1) there is an inner loop *L* with loop counter *c*, such that *c* is *increased* on an outer loop of *L*. (2) Nevertheless, the *amortized cost of L* (the overall worst-case cost of executing *L*, averaged over the number of executions of its outer loop) is lower than the worst-case cost of a *single* execution (a single instance of *consecutive* iterations) of *L*.

**Table 12:** Tool results on 23 challenging loop iteration patterns from *cBench* and *SPEC CPU 2006* Benchmarks

		loopus’15	loopus’14	CoFloCo	KoAT	Rank	C4B
cf_decode_eol	*O*(*n*)	$\checkmark$	$\checkmark$	$\times$	$\mathbf {O(n^{2})}$	$\times$	$\times$
cryptRandWriteFile	*O*(*n*)	$\checkmark$	$\mathbf {O(n^{2})}$	$\checkmark$	$\mathbf {O(n^{2})}$	$\checkmark$	$\checkmark$
encode_mcu_AC_refine	*O*(*n*)	$\checkmark$	$\checkmark$	$\times$	$\mathbf {O(n^{2})}$	$\checkmark$	$\times$
hc_compute	$O(n^2)$	$\checkmark$	$\mathbf {O(n^{3})}$	$\mathbf {O(n^{3})}$	**TO**	$\checkmark$	$\times$
inflated_stored	*O*(*n*)	$\checkmark$	$\checkmark$	$\checkmark$	$\times$	$\times$	$\times$
PackBitsEncode	*O*(*n*)	$\checkmark$	$\times$	**TO**	$\mathbf {O(n^{2})}$	$\mathbf {\bigcirc }$	$\times$
s_SFD_process	*O*(*n*)	$\checkmark$	$\checkmark$	$\times$	$\mathbf {O(n^{2})}$	$\times$	$\times$
send_tree	*O*(*n*)	$\checkmark$	$\checkmark$	$\mathbf {O(n^{2})}$	$\mathbf {O(n^{2})}$	$\mathbf {O(n^{2})}$	$\times$
sendMTFValues	*O*(*n*)	$\checkmark$	$\checkmark$	$\mathbf {O(n^{2})}$	$\checkmark$	$\mathbf {O(n^{2})}$	$\checkmark$
set_color_ht	$O(n^2)$	$\checkmark$	$\mathbf {O(n^{3})}$	$\checkmark$	$\mathbf {O(n^{3})}$	$\times$	$\times$
subsetdump	*O*(*n*)	$\checkmark$	$\checkmark$	$\mathbf {O(n^{2})}$	$\mathbf {O(n^{2})}$	$\times$	$\times$
zwritehexstring_at	*O*(*n*)	$\checkmark$	$\mathbf {O(n^{2})}$	$\checkmark$	$\mathbf {O(n^{2})}$	$\checkmark$	$\checkmark$
analyse_other	$O(n^3)$	$\mathbf {O(n^{4})}$	$\mathbf {O(n^{4})}$	$\times$	$\mathbf {O(n^{13})}$	$\times$	$\times$
ApplyBndRobin	$O(n^4)$	$\checkmark$	$\checkmark$	$\times$	$\checkmark$	$\mathbf {\bigcirc }$	$\mathbf {\bigcirc }$
asctoeg	$O(n^2)$	$\checkmark$	$\checkmark$	$\checkmark$	$\mathbf {O(n^{3})}$	$\checkmark$	$\times$
Configure	*O*(*n*)	$\mathbf {O(n^{2})}$	$\mathbf {O(n^{2})}$	$\mathbf {O(n^{2})}$	$\mathbf {O(n^{2})}$	$\checkmark$	$\times$
load_mems	*O*(*n*)	$\checkmark$	$\mathbf {O(n^{3})}$	$\times$	$\mathbf {O(n^{3})}$	$\times$	$\checkmark$
local_alloc	*O*(*n*)	$\checkmark$	$\mathbf {O(n^{2})}$	$\checkmark$	$\mathbf {O(n^{2})}$	$\checkmark$	$\checkmark$
ParseFile	*O*(*n*)	$\checkmark$	$\checkmark$	**TO**	$\mathbf {O(n^{3})}$	$\mathbf {\bigcirc }$	$\times$
Perl_scan_vstring	*O*(*n*)	$\checkmark$	$\mathbf {O(n^{2})}$	$\times$	$\mathbf {O(n^{2})}$	$\times$	$\checkmark$
SingleLinkCluster	$O(n^2)$	$\checkmark$	$\checkmark$	$\times$	**TO**	$\times$	$\times$
xdr3dfcoord	*O*(*n*)	$\checkmark$	$\checkmark$	$\mathbf {O(n^{2})}$	$\mathbf {O(n^{2})}$	$\mathbf {\bigcirc }$	$\times$
XNU	*O*(*n*)	$\checkmark$	$\mathbf {O(n^{2})}$	$\mathbf {O(n^{2})}$	$\mathbf {O(n^{2})}$	$\times$	$\times$
Total tight		*21*	*12*	*6*	*2*	*7*	*6*
Total Over-approx.		*2*	*10*	*7*	*18*	*2*	*0*
Total fail		*0*	*1*	*8*	*1*	*10*	*16*
Total timed Out		*0*	*0*	*2*	*2*	*0*	*0*
Total time		*2 s*	*1 s*	*41 m*	*74 m*	*28 s*	*20 m*
Total time w/o TO		*2 s*	*1 s*	*1 m*	*34 m*	*28 s*	*20 m*

The time out was 20 min, a longer time out did not yield additional results

Example xnu (discussed in Sect. 7) is a natural example for the described behaviour.

The complete benchmark is available at [19]. For each pattern we link its origin in the header of the respective file. Note that for some patterns we found several instances.

Table 12 states the results that were obtained by loopus’15, loopus’14, *CoFloCo*, *KoAT*, *Rank* and *C4B*: ‘$\checkmark$’ denotes that the bound computed by the respective tool is *tight* (in the same asymptotic class as the precise bound, see Definition 5), ‘$\mathbf {O(n^{x})}$’ denotes that the respective tool did not infer a *tight* bound but a bound in the asymptotic class $\mathbf {O(n^{x})}$, ‘$\times$’ denotes that no bound was inferred, ‘**TO**’ denotes that the tool timed out (the time out limit was 20 min, a longer time out did not yield additional results), ‘$\mathbf {\bigcirc }$’ denotes that we were not able to translate the example into the input format of the tool. For each file we annotate its asymptotic complexity (an asymptotic bound on the total number of loop iterations, determined manually) behind its file name in Table 12.

We explain the last 5 rows of Table 12: ‘**Total Tight**’ states the number of examples for which the respective tool inferred a *tight* bound (see Definition 5). ‘**Total Over-approx.**’ states the number of examples for which the respective tool inferred a bound that is *not* tight. ‘**Total Fail**’ states the number of examples for which the respective tool did not report a bound, but returned within the time out limit of 20 min. ‘**Total Timed Out**’ states the total number of examples on which the respective tool timed out (the time out limit was 20 min). ‘**Total Time**’ states the overall time consumed by the respective tool for processing the complete benchmark. ‘**Total Time w/o TO**’ states the overall time consumed by the respective tool on those examples on which the tool did not time out.

loopus’15 fails to infer a *tight* bound only for Configure and analyse_other. For both examples a precise bound can be obtained by an improvement of our variable bound function ($V\mathcal {B}$) described in [30] which is not yet implemented into our tool. loopus’15 is far more successful in inferring tight bounds for the examples than any of the competitors. loopus’15 infers 21, loopus’14 12, *Rank* 7, *C4B* 6, *CoFloCo* 6 and *KoAT* 2 tight bounds. There are 9 examples for which *only* our tool loopus (loopus’15 and loopus’14) infers a tight bound: cf_decode_eol, PackBitsEncode, s_SFD_process, send_tree, subsetdump, ParseFile, SingleLinkCluster,xdr3dfcoord, and XNU.

The experiment demonstrates, that our bound analysis complements the state-of-the-art, by inferring tight bounds for a class of real-world loop iterations, on which existing techniques mostly fail or obtain coarse over-approximations.

*Technical remarks* (1) We counted the time needed by the tool *Aspic* (a preprocessor for *Rank* which performs *invariant generation*) into the time of the bound analysis performed by *Rank*. (2) *Rank* reported an unsound bound and an error message for the examples s_SFD_process.c, load_mems.c and SingleLinkCluster.c. On these examples we therefore assessed *Rank*’s return value as *fail* (‘$\times$’).

## Amortized Complexity Analysis

In the following we discuss how our approach relates to *amortized complexity analysis* as introduced by Tarjan in his influential paper [32]. We recall Tarjan’s idea of using *potential functions* for amortized analysis in Sect. 9. In Sect. 9.1 we explain how our approach can be viewed as an instantiation of amortized analysis via potential functions.

*Amortized Analysis using Potential Functions* Amortized complexity analysis [32] aims at inferring the worst-case average cost over a sequence of calls to an operation or function rather than the worst-case cost of a single call. In (resource) bound analysis the difference between the single worst-case cost and the amortized cost is relevant, e.g., if a function $\mathtt {f}$ is called inside a loop: assume the loop bound is *n* and the single worst-case cost of a call to $\mathtt {f}$ is also *n*. The cost of a single call to $\mathtt {f}$ *amortized* over all *n* calls might, however, be lower than *n*, e.g., 2. In this case the total worst-case cost of iterating the loop is 2*n* rather than $n^2$. Note that in our non-recursive setting function calls can always be inlined. The amortized analysis problem thus boils down to the problem of inferring the cost of executing an inner loop averaged over all executions of the outer loop.

Tarjan [32] motivates amortized complexity analysis on the example of a program which executes *n* stack operations $\mathtt {StackOp}$. Each $\mathtt {StackOp}$ operation consists of a $push$ instruction, adding an element to the stack, followed by a $pop$ instruction, removing an arbitrary number of elements from the stack. Initially the stack is empty. The *cost* of a single $push$ is 1 and the *cost* of a single $pop$ is the number of elements removed from the stack. Tarjan points out that the worst-case cost of a single $pop$ is *n*: the *n*th $pop$ instruction may pop *n* elements (cost *n*) from the stack, if the previous $pop$ instructions did not remove any elements from the stack. i.e., the worst-case cost of a single $\mathtt {StackOp}$ operation is $n+1$. Nevertheless all *n* operations $\mathtt {StackOp}$ cannot cost more than 2*n* in total since we cannot remove more elements from the stack than have been added to the stack and thus the overall cost of the $pop$ instructions is bounded by the total number of $push$ instructions (*n* by assumption). The *amortized cost* of $\mathtt {StackOp}$, i.e., the cost of $\mathtt {StackOp}$ averaged over the sequence of all *n* operations, is therefore 2.

*Potential Function* As a means to reason about the amortized cost of an operation or a sequence of operations, Tarjan introduces the notion of a *potential function*. A potential function is a function ${\varPhi }: {\varSigma }\rightarrow \mathbb {Z}$ from the program states to the integers. Let $\mathcal {C}_{\mathtt {op}}(\sigma )$ denote the cost of executing operation $\mathtt {op}$ at program state $\sigma \in {\varSigma }$. Let ${\varPhi }$ be a potential function. Tarjan defines the *amortized cost* $\mathcal {C} _{\mathtt {op}}^\mathcal {A}(\sigma )$ as $\mathcal {C} _{\mathtt {op}}^\mathcal {A}(\sigma ) = \mathcal {C}_{\mathtt {op}}(\sigma ) + {\varPhi }(\sigma ^\prime ) - {\varPhi }(\sigma )$ where $\sigma$ denotes the program state before and $\sigma ^\prime$ denotes the program state after executing $\mathtt {op}$. I.e., the amortized cost is the cost plus the decrease resp. increase in the value of the potential. Consider a sequence of *n* operations, let $\mathtt {op}_i$ denote the *i*th operation in the sequence. Let $\sigma _i$ denote the program state before executing operation $\mathtt {op}_{i}$, $\sigma _{i+1}$ is the program state after executing $\mathtt {op}_{i}$. In general, the total cost of executing all *n* operations is:

1 $$\begin{aligned} \sum \limits _{i=1}^{n} \mathcal {C}_{\mathtt {op}_i}(\sigma _i) = \sum \limits _{i=1}^{n} \mathcal {C} _{\mathtt {op}_i}^\mathcal {A}(\sigma _i) - {\varPhi }(\sigma _{i+1}) + {\varPhi }(\sigma _{i}) = {\varPhi }(\sigma _1) - {\varPhi }(\sigma _{n+1}) + \sum \limits _{i=1}^{n} \mathcal {C} _{\mathtt {op}_i}^\mathcal {A}(\sigma _i) \end{aligned}$$

*That is, the total time of the operations equals the sum of their amortized times plus the net decrease in potential from the initial to the final configuration. [...] In most cases of interest, the initial potential is zero and the potential is always non-negative. In such a situation the total amortized time is an upper bound on the total time* [32]. I.e., if ${\varPhi }_i \ge 0$ and ${\varPhi }_0 = 0$ then

2 $$\begin{aligned} \sum \limits _{i=1}^{n} \mathcal {C}_{\mathtt {op}_i}(\sigma _i) \le \sum \limits _{i=1}^{n} \mathcal {C} _{\mathtt {op}_i}^\mathcal {A}(\sigma _i) \end{aligned}$$

Reconsider Tarjan’s previously discussed example of a sequence of *n* executions of operation $\mathtt {StackOp}$. Let $j$ denote the stack size, i.e., $\sigma _i( j )$ is the size of the stack in program state $\sigma _i$. The cost of executing $\mathtt {StackOp}$ in program state $\sigma _i$ is $\mathcal {C}_{\mathtt {StackOp}}(\sigma _i) = 1 + (\sigma _i( j ) + 1 - \sigma _{i+1}( j ))$ (where $\sigma _i( j ) + 1 - \sigma _{i+1}( j )$ is the cost of the pop operation). Tarjan proposes to use the stack size *j* as a potential function, i.e. we choose ${\varPhi }(\sigma _i) = \sigma _i( j )$. We have

$$\begin{aligned} \mathcal {C} _{\mathtt {StackOp}}^\mathcal {A}(\sigma _i)&= \mathcal {C}_{\mathtt {StackOp}}(\sigma _i) + {\varPhi }(\sigma _{i+1}) - {\varPhi }(\sigma _i)\\&= \mathcal {C}_{\mathtt {StackOp}}(\sigma _i) + \sigma _{i+1}( j ) - \sigma _i( j )\\&= 1 + (\sigma _i( j ) + 1 - \sigma _{i+1}( j ) + \sigma _{i+1}( j ) - \sigma _i( j )\\&= 2 \end{aligned}$$

With (2) we get:

$$\begin{aligned} \sum \limits _{i=1}^{n} \mathcal {C}_{\mathtt {StackOp}_i}(\sigma _i) \le \sum \limits _{i=1}^{n} \mathcal {C} _{\mathtt {StackOp}_i}^\mathcal {A}(\sigma _i) = \sum \limits _{i=1}^{n} 2 = 2n \end{aligned}$$

**Fig. 12:**
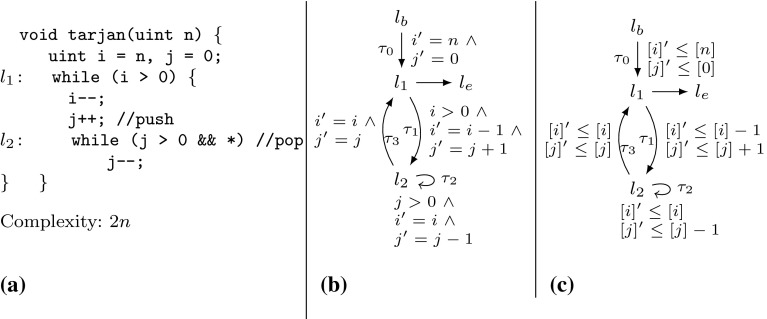
**a** Example tarjan  **b** LTS of Example tarjan  **c** $DCP$ obtained by abstraction from tarjan

### Amortized Analysis in our Algorithm

Example tarjan in Fig. 12 is a model of Tarjan’s motivating example (discussed above): variable *j* models the stack size. The $push$ instruction is modeled by increasing the stack size *j* by 1. The $pop$ instruction is modeled by decreasing the stack size. Further all calls to $\mathtt {StackOp}$, $push$ and $pop$ are *inlined*. Consider the *labeled transition system* of Example tarjan shown in Fig. 12b. We have that transition $\tau _1$ models the $push$ instruction, increasing the stack size *j* by 1, a sequence of transitions $\tau _2$ models the $pop$ instruction, decreasing the stack by an arbitrary number of elements. A *complete* run $\rho$ of Example tarjan can be decomposed into the initial transition $\tau _0$ and a number of sub-runs $\rho _{[i_k,i_{k+1}]}$ with $1 \le i_1 < i_2 \ldots$ s.t. each $\rho _{[i_k,i_{k+1}]}$ consists of a single transition $\tau _1$ ($push$) followed by a sequence of transitions $\tau _2$ ($pop$), followed by a single execution of transition $\tau _3$. Each sub-run $\rho _{[i_k,i_{k+1}]}$ models Tarjan’s $\mathtt {StackOp}$ operation. We thus have that the *amortized cost* of a sub-run $\rho _{[i_k,i_{k+1}]}$ is 2. Given that $\tau _1$ cannot be executed more than *n* times and each $\rho _{[i_k,i_{k+1}]}$ contains exactly one $\tau _1$, we get that the overall cost of executing Example tarjan is bounded by $n \times 2 = 2n$.

In the following we argue that our transition bound algorithm $T\mathcal {B}$ is an instantiation of amortized analysis using *potential functions*. We base our discussion on the concrete semantics of Example tarjan given by the LTS in Fig. 12b. Note, however, that our algorithm runs on the abstracted $DCP$ in Fig. 12c where the same reasoning applies: suppose we want to compute the transition bound of transition $\tau _2$ in order to compute the total cost of the $pop$ instructions. Let $\rho = (\sigma _0,l_0) \xrightarrow {\lambda _0} (\sigma _1, l_1) \xrightarrow {\lambda _1} \cdots$ be a run of Example tarjan. Let $len (\rho )$ denote the *length* of $\rho$ (i.e, total number of transitions on $\rho$). We define the cost of executing $\tau _2$ in program state $\sigma _i$ as $C_{\tau _2}(\sigma _i) = 1$ and the cost of executing $\tau _1$ and $\tau _3$ as $C_{\tau _1}(\sigma _i) = C_{\tau _3}(\sigma _i) = 0$ since we are only interested in $\tau _2$. We have

$$\begin{aligned} \sharp (\tau _2,\rho ) = \sum \limits _{i = 1}^{ len (\rho )-1} C_{\rho (i)}(\sigma _i) \end{aligned}$$

where $\rho (i)$ denotes the $i+1$th transition $l_i \xrightarrow {u_i} l_{i+1}$ on $\rho$. Our algorithm reduces the question “how often can $\tau _2$ be executed?” to the question “how often can the local bound ’*j*’ of $\tau _2$ be increased on $\tau _1$?”. This reasoning uses the *local bound* *j* of $\tau _2$ as a *potential function*, as we show next: we get the following *amortized* costs for executing $\tau _1,\tau _2$ and $\tau _3$ respectively:

$$\begin{aligned} C_{\tau _2}^\mathcal {A}(\sigma _i)&= C_{\tau _2}(\sigma _i) + \sigma _{i+1}(j) - \sigma _i(j) = 1 + \sigma _{i+1}(j) - \sigma _i(j) = 0\\ C_{\tau _1}^\mathcal {A}(\sigma _i)&= C_{\tau _1}(\sigma _i) + \sigma _{i+1}(j) - \sigma _i(j) = 0 + \sigma _{i+1}(j) - \sigma _i(j) = 1\\ C_{\tau _3}^\mathcal {A}(\sigma _i)&= C_{\tau _3}(\sigma _i) + \sigma _{i+1}(j) - \sigma _i(j) = 0 + \sigma _{i+1}(j) - \sigma _i(j) = 0 \end{aligned}$$

With $\sigma _i(j) \ge 0$, $\sigma _1(j) = 0$ and (2) we have:

$$\begin{aligned} \sharp (\tau _2,\rho ) = \sum \limits _{i = 1}^{ len (\rho )-1} C_{\rho (i)}(\sigma _i) \le \sum \limits _{i = 1}^{ len (\rho )-1} C_{\rho (i)}^\mathcal {A}(\sigma _i) = \sharp (\tau _1,\rho ) \times 1 \end{aligned}$$

We point out that choosing the *local bound* *j* of $\tau _2$ as potential function causes the amortized cost of executing $\tau _2$ to be 0 and reduces the question how often $\tau _2$ can be executed to how often the potential *j* can be incremented on $\tau _1$.

Using $\sharp (\tau _1,\rho ) \le \sharp (\tau _0,\rho ) \times n = n$ one obtains the upper bound *n* for the total cost of the $pop$ instructions.

## Conclusion

We presented a new approach to (resource) bound analysis. Our approach complements existing approaches in several aspects, as discussed in Sect. 2.3. Our analysis handles bound analysis problems of high practical relevance which current approaches cannot handle: current techniques [6, 7, 10, 29] fail on Example xnu and similar problems. We have argued that such problems, e.g., occur naturally in parsing and string matching routines. During our experiments on real-world source code, we found 23 different iteration patterns that pose a challenge for similar reasons as Example xnu: in these patterns, the worst-case cost of a single inner loop execution is lower than the worst-case cost of the inner loop averaged over the iterations of the outer loop. Our implementation obtains tight bounds for 21 out of these 23 iteration patterns (Sect. 8.3).

Our algorithm (Sect. 3) obtains invariants by means of bound analysis and does not rely on external techniques for invariant generation. This is in contrast to current bound analysis techniques (see discussion on related work in Sect. 2). We have compared our algorithm to classical invariant analysis and argued that we can efficiently compute invariants which are difficult to obtain by standard abstract domains such as octagon or polyhedra (Sect. 2). We have demonstrated that the limited form of invariants (upper bound invariants) that our algorithm obtains is sufficient for the bound analysis of a large class of real-world programs.

We have demonstrated that *difference constraints* are a suitable abstract program model for automatic complexity and resource bound analysis. Despite their syntactic simplicity, difference constraints are expressive enough to model the complexity-related aspects of many imperative programs. In particular, difference constraints allow to model *amortized complexity* problems such as the bound analysis challenge posed by Example xnu (discussed in Sect. 7). We developed appropriate techniques for abstracting imperative programs to $DCPs$ (Sect. 6): we described how to extract *norms* (integer-valued expressions over the program state) from imperative programs and showed how to use these norms as variables in $DCPs$.

Our approach deals with many challenges bound analysis is known to be confronted: in Sect. 8.2 we compared our tool on a benchmark of challenging problems from publications on bound analysis. The results show that our prototype implementation can handle most of these problems. Here, our implementation, while comparable in terms of strengths to other implementations of state-of-the-art bound analysis techniques, performs the task significantly faster than the competitors. The results obtained by our prototype tool could be further enhanced by extending our implementation with additional techniques discussed in [30].

We stress that our approach is more *scalable* than existing approaches. We presented empirical evidence of the good performance characteristics of our analysis by a large experiment and tool comparison on real source code in Sect. 8.1. We discuss the main technical reasons for scalability of our analysis in Sect. 10.1.

We think that the abstract program model of difference constraint programs is worth further investigation: given that difference constraints can model standard counter manipulations (counter increments, decrements and resets), a further research on complexity analysis of difference constraint programs is of high value. We consider $DCP$s to be a very suitable program model for studying the principle challenges of automated complexity and resource bound analysis for imperative programs.

### Discussion on the Scalability of Our Analysis

In the following we state what we consider to be the main technical reasons that make our analysis scale:

First of all, we achieve scalability by *local* reasoning: note that our abstraction procedure relies on purely *local information*, i.e., information that is available on *single* program transitions. In particular, we do not apply global invariant analysis. Further, the sets $\mathcal {I}(\mathtt {v})$ and $\mathcal {R}(\mathtt {v})$, by which our main algorithm is parametrized, are built by categorizing the difference constraints on *single* (abstract) program transitions based on simple syntactic criteria. Our algorithm for computing the local bound mapping $\zeta$ (Sect. 4) is *polynomial* even in the generalized case (Sect. 4.1).

We use bound analysis to infer bounds on variable values (variable bounds). Unlike classical invariant analysis this approach is *demand-driven* and does *not* perform a *fixed point* iteration (see discussion in Sect. 2.3).

Note that the only general purpose reasoner we employ is an SMT solver. Further, the SMT solver is only employed in the program abstraction phase. In terms of size, the problems we feed to the SMT solver are *small*, namely simple linear arithmetic formulas, composed of the arithmetic of single transitions. Our approach instruments the SMT solver only for *yes/no* answers, no *optimal* solution (e.g., minimum or minimal unsatisfiable core) is required.

Our basic bound algorithm (Definition 19) runs in *polynomial time*. The reasoning based on reset chains (Definition 23), however, has *exponential* worst-case complexity, resulting from the potentially exponential number of paths in the program (exponential in the number of program transitions). We did not experience this to be an issue in practice because the *simplicity* of our abstract program model allows to take straightforward engineering measures: program slicing reduces the number of paths in the program *significantly*, further, *merging* of similar paths can be applied (details are given in [28]).

## Electronic supplementary material

Below is the link to the electronic supplementary material.

Supplementary material 1 (pdf 303 KB)

## Footnotes

1
https://www.spec.org/cpu2006/.

2
https://github.com/s-falke/kittel-koat, https://github.com/aeflores/CoFloCo.
