# Supplementary material for: Complexity and Resource Bound Analysis of Imperative Programs Using Difference Constraints
Source: J Autom Reason. 2017 Jan 11;59(1):3–45. doi: 10.1007/s10817-016-9402-4 (PMC6044401; doi:10.1007/s10817-016-9402-4)
Supplement: Supplementary file 1 — Supplementary material 1 (pdf 303 KB) [file 10817_2016_9402_MOESM1_ESM.pdf]

A Soundness Proofs

We prove soundness of our basic bound algorithm for *DCP*s (Definition 19, Theorem 1) in Section A.1. In Section A.2 we prove soundness of our reasoning on reset chains (Definition 23, Theorem 2). Throughout this section we assume a *well-defined* and *fan-in free DCP* $\Delta\mathcal{P}(L, E, l_b, l_e)$ over $\mathcal{A}$ to be given.

We first define some basic notions which we use to state our proofs precisely.

Definition A.1 (Indices) Let $\pi = l_0 \xrightarrow{u_0} l_1 \xrightarrow{u_1} \dots$ be a path of $\Delta\mathcal{P}$. By $len(\pi)$ we denote the *length* of π, i.e., the total number of transitions on π (possibly ∞). Let $0 \leq i \leq j$. By $\pi_{[i,j]}$ we denote the sub-path of π that starts at l_i and ends at l_j. By $\pi(i) = l_i \xrightarrow{u_i} l_{i+1}$ we denote the $(i+1)$th transition on π.
Let $\tau \in E$. We define $\Theta(\tau, \pi) = \{0 \leq i < len(\pi) \mid \pi(i) = \tau\}$. Let $E' \subseteq E$. We define $\Theta(E', \pi) = \bigcup_{\tau \in E'} \Theta(\tau, \pi)$. We write $\Theta(\mathcal{R}(\mathtt{v}), \pi)$ to denote $\Theta(\{\tau \mid (\tau, \_, \_) \in \mathcal{R}(\mathtt{v})\}, \pi)$, and $\Theta(\mathcal{I}(\mathtt{v}), \pi)$ to denote $\Theta(\{\tau \mid (\tau, \_) \in \mathcal{I}(\mathtt{v})\}, \pi)$. We use the same notation for runs ρ of $\Delta\mathcal{P}$.

I.e., $\Theta(\tau, \pi)$ is the set of all indices of τ on π, $\Theta(\mathcal{R}(\mathtt{v}), \pi)$ is the set of indices of all transitions on π which reset $\mathtt{v}$ and $\Theta(\mathcal{I}(\mathtt{v}), \pi)$ is the set of indices of all transitions on π which increment $\mathtt{v}$.

On a run of $\Delta\mathcal{P}$ a variable $\mathtt{v}$ may take arbitrary values at locations at which $\mathtt{v}$ is not defined, i.e., at locations l with $\mathtt{v} \notin \mathtt{def}(l)$. In a well-defined *DCP* the value of a variable at a location where it is not defined can, however, not affect the program's behaviour. This observation motivates the notion of a *normalized run*: a normalized run is a run on which a variable takes value '0' at locations where it is not defined.

Definition A.2 (Normalized Run) Let $\rho = (l_0, \sigma_0) \xrightarrow{u_0} (l_1, \sigma_1) \xrightarrow{u_1} \cdots$ be a run of $\Delta\mathcal{P}$. Let
$\sigma'_i(\mathtt{a}) = \begin{cases} 0 & \text{if } \mathtt{a} \in \mathcal{V} \text{ and } \mathtt{a} \notin \mathtt{def}(l_i) \\ \sigma_i(\mathtt{a}) & \text{else} \end{cases}$ for all $0 \leq i \leq len(\rho)$ and all $\mathtt{a} \in \mathcal{A}$.

We call $\lfloor\rho\rfloor = (l_0, \sigma'_0) \xrightarrow{u_0} (l_1, \sigma'_1) \xrightarrow{u_1} \cdots$ a *normalized run*.

Let Ξ be a set of runs of $\Delta\mathcal{P}$. We say that Ξ is closed under *normalization* if $\rho \in \Xi$ implies that $\lfloor\rho\rfloor \in \Xi$.

Lemma A.1 states that the set of all runs of $\Delta\mathcal{P}$ is closed under normalization.

Lemma A.1 *Let ρ be a run of $\Delta\mathcal{P}$. Then $\lfloor\rho\rfloor$ is a run of $\Delta\mathcal{P}$.*

Proof Follows directly from Definition 14 (well-definedness) and Definition A.2. □

A.1 Soundness of Basic Bound Algorithm

In Lemma A.2 and Lemma A.3 we formulate the two key insights on which our algorithm is based. Lemma A.2 formalizes the intuition given in Section 9: Let $\mathtt{v}$ be a local transition bound for τ. The question how often τ can appear on a run ρ is translated to the question how often the transitions which increase the value of $\mathtt{v}$ (i.e., $(t, \_) \in \mathcal{I}(\mathtt{v})$ and $(t, \_, \_) \in \mathcal{R}(\mathtt{v})$) can appear on ρ.

Lemma A.2 *Let ρ be a run of $\Delta\mathcal{P}$. Let $\tau \in E$. Let $\mathtt{v} \in \mathcal{V}$ be a* local transition bound *for τ on $\lfloor\rho\rfloor$. Let $vb : \mathcal{A} \to \mathbb{Z}$ be s.t. $vb(\mathtt{a})$ is a* variable bound *for $\mathtt{a}$ on ρ for all $(\_, \mathtt{a}, \_) \in \mathcal{R}(\mathtt{v})$. Then*

$$\left(\sum_{(t,\mathtt{c})\in\mathcal{I}(\mathtt{v})} \sharp(t,\rho) \times \mathtt{c}\right) + \sum_{(t,\mathtt{a},\mathtt{c})\in\mathcal{R}(\mathtt{v})} \sharp(t,\rho) \times (vb(\mathtt{a}) + \mathtt{c})$$

is a transition bound *for τ on ρ.*

Proof We first show that it is sufficient to consider the case $\lfloor\rho\rfloor = \rho$:
1. Let $\mathtt{expr}$ be a *transition bound* for τ on $\lfloor\rho\rfloor$. Then $\mathtt{expr}$ is also a *transition bound* for τ on ρ (follows directly from Definition A.2).
2. By assumption $vb(\mathtt{a})$ is a *variable bound* for $\mathtt{a}$ on ρ. By Definition A.2 we have that $vb(\mathtt{a})$ is also a *variable bound* for $\mathtt{a}$ on $\lfloor\rho\rfloor$. We thus assume that $\lfloor\rho\rfloor = \rho$.

We have to show:

$$\sharp(\tau,\rho) \leq \left(\sum_{(t,\mathtt{c})\in\mathcal{I}(\mathtt{v})} \sharp(t,\rho) \times \mathtt{c}\right) + \sum_{(t,\mathtt{a},\mathtt{c})\in\mathcal{R}(\mathtt{v})} \sharp(t,\rho) \times (vb(\mathtt{a}) + \mathtt{c})$$

A) We first show that

$$\sharp(\tau,\rho) \leq \left(\sum_{(t,\mathtt{c})\in\mathcal{I}(\mathtt{v})} \sharp(t,\rho) \times \mathtt{c}\right) + \sum_{j\in\Theta(\mathcal{R}(\mathtt{v}),\rho)} \sigma_{j+1}(\mathtt{v})$$

We have

$$\begin{aligned}
\sharp(\tau,\rho) &\overset{(1)}{\leq} \sharp(\tau,\rho) + \sum_{i=0}^{len(\rho)-1} \sigma_{i+1}(\mathtt{v}) - \sigma_i(\mathtt{v}) \\
&\overset{(2a)}{=} \sharp(\tau,\rho) + \sum_{i=0}^{len(\rho)-1} \max(\sigma_{i+1}(\mathtt{v}) - \sigma_i(\mathtt{v}), 0) + \sum_{i=0}^{len(\rho)-1} \min(\sigma_{i+1}(\mathtt{v}) - \sigma_i(\mathtt{v}), 0) \\
&\overset{(2)}{\leq} \sum_{i=0}^{len(\rho)-1} \max(\sigma_{i+1}(\mathtt{v}) - \sigma_i(\mathtt{v}), 0) \\
&\overset{(3a)}{=} \left(\sum_{i\in\Theta(\mathcal{I}(\mathtt{v}),\rho)} \max(\sigma_{i+1}(\mathtt{v}) - \sigma_i(\mathtt{v}), 0)\right) + \sum_{i\in\Theta(\mathcal{R}(\mathtt{v}),\rho)} \max(\sigma_{i+1}(\mathtt{v}) - \sigma_i(\mathtt{v}), 0) \\
&\overset{(3)}{\leq} \left(\sum_{i\in\Theta(\mathcal{I}(\mathtt{v}),\rho)} \max(\sigma_{i+1}(\mathtt{v}) - \sigma_i(\mathtt{v}), 0)\right) + \sum_{j\in\Theta(\mathcal{R}(\mathtt{v}),\rho)} \sigma_{j+1}(\mathtt{v}) \\
&\overset{(4)}{\leq} \left(\sum_{(t,\mathtt{c})\in\mathcal{I}(\mathtt{v})} \sum_{0\leq i<len(\rho) \text{ s.t. } \rho(i)=t} \mathtt{c}\right) + \sum_{j\in\Theta(\mathcal{R}(\mathtt{v}),\rho)} \sigma_{j+1}(\mathtt{v}) \\
&\overset{(5)}{=} \left(\sum_{(t,\mathtt{c})\in\mathcal{I}(\mathtt{v})} \sharp(t,\rho) \times \mathtt{c}\right) + \sum_{j\in\Theta(\mathcal{R}(\mathtt{v}),\rho)} \sigma_{j+1}(\mathtt{v})
\end{aligned}$$

(1) We have $\sum_{i=0}^{len(\rho)-1} \sigma_{i+1}(\mathtt{v}) - \sigma_i(\mathtt{v}) = \sigma_{len(\rho)}(\mathtt{v}) - \sigma_0(\mathtt{v}) = \sigma_{len(\rho)}(\mathtt{v})$
because $\sigma_0(\mathtt{v}) = 0$ with i) $\rho = \lfloor \rho \rfloor$ and ii) $\mathtt{v} \notin \mathtt{def}(l_b)$ (Definition 14).
Trivially $\sigma_{len(\rho)}(\mathtt{v}) \geq 0$. Therefore $\sum_{i=0}^{len(\rho)-1} \sigma_{i+1}(\mathtt{v}) - \sigma_i(\mathtt{v}) \geq 0$.

(2a) Case Distinction

(2) We have $\sharp(\tau, \rho) \leq \downarrow(\mathtt{v}, \rho)$ (Definition 9).
Further $\downarrow(\mathtt{v}, \tau) \leq \left(\sum_{i=0}^{len(\rho)-1} \min(\sigma_{i+1}(\mathtt{v}) - \sigma_i(\mathtt{v}), 0) \right) \times -1$.
Thus $\sharp(\tau, \rho) + \sum_{i=0}^{len(\rho)-1} \min(\sigma_{i+1}(\mathtt{v}) - \sigma_i(\mathtt{v}), 0) \leq 0$.

(3a) $\sigma_{i+1}(\mathtt{v}) - \sigma_i(\mathtt{v}) > 0$ implies in particular that $\sigma_{i+1}(\mathtt{v}) > 0$. Thus $\mathtt{v} \in \mathtt{def}(l_{i+1})$ because $\rho = \lfloor \rho \rfloor$ by assumption. With $\sigma_{i+1}(\mathtt{v}) > \sigma_i(\mathtt{v})$ we have that either:
Case 1) $(\rho(i), \_) \in \mathcal{I}(\mathtt{v})$, i.e., $i \in \Theta(\mathcal{I}(\mathtt{v}), \rho)$, or
Case 2) $(\rho(i), \_, \_) \in \mathcal{R}(\mathtt{v})$, i.e., $i \in \Theta(\mathcal{R}(\mathtt{v}), \rho)$.

(3) Since $\sigma_i(\mathtt{v}) \geq 0$ we have that $\sigma_{i+1}(\mathtt{v}) - \sigma_i(\mathtt{v}) \leq \sigma_{i+1}(\mathtt{v})$.

(4) If $i \in \Theta(\mathcal{I}(\mathtt{v}), \rho)$ then there is $(t, \mathtt{c}) \in \mathtt{Incr}(\mathtt{v})$ s.t. $\rho(i) = t$ (Definition A.1).
Further $\sigma_{i+1}(\mathtt{v}) - \sigma_i(\mathtt{v}) \leq \mathtt{c}$ and $\mathtt{c} > 0$ (Definition 18).

(5) By definition of $\sharp(t, \rho)$ (Definition 7).

B) We show that $\sum_{j \in \Theta(\mathcal{R}(\mathtt{v}), \rho)} \sigma_{j+1}(\mathtt{v}) \leq \sum_{(t,\mathtt{a},\mathtt{c}) \in \mathcal{R}(\mathtt{v})} \sharp(t, \rho) \times (vb(\mathtt{a}) + \mathtt{c})$:

$$\begin{aligned}
\sum_{j \in \Theta(\mathcal{R}(\mathtt{v}), \rho)} \sigma_{j+1}(\mathtt{v}) &\overset{(1)}{=} \sum_{(t,\mathtt{a},\mathtt{c}) \in \mathcal{R}(\mathtt{v})} \sum_{j \in \Theta(t,\rho)} \sigma_{j+1}(\mathtt{v}) \\
&\overset{(2)}{\leq} \sum_{(t,\mathtt{a},\mathtt{c}) \in \mathcal{R}(\mathtt{v})} \sum_{j \in \Theta(t,\rho)} \sigma_j(\mathtt{a}) + \mathtt{c} \\
&\overset{(3)}{\leq} \sum_{(t,\mathtt{a},\mathtt{c}) \in \mathcal{R}(\mathtt{v})} \sum_{j \in \Theta(t,\rho)} vb(\mathtt{a}) + \mathtt{c} \\
&\overset{(4)}{=} \sum_{(t,\mathtt{a},\mathtt{c}) \in \mathcal{R}(\mathtt{v})} \sharp(t, \rho) \times (vb(\mathtt{a}) + \mathtt{c})
\end{aligned}$$

(1) By commutativity: Let $j \in \Theta(\mathcal{R}(\mathtt{v}), \rho)$. By the assumption that $\Delta\mathcal{P}$ is *fan-in free* there is only exactly one $\mathtt{a} \in \mathcal{A}$ and exactly one $\mathtt{c} \in \mathbb{Z}$ s.t. $(\rho(j), \mathtt{a}, \mathtt{c}) \in \mathcal{R}(\mathtt{v})$.

(2) With $(\rho(j), \mathtt{a}, \mathtt{c}) \in \mathcal{R}(\mathtt{v})$ we have that $\sigma_{j+1}(\mathtt{v}) \leq \sigma_j(\mathtt{a}) + \mathtt{c}$ (Definition 18).

(3) Let $(t, \mathtt{a}, \_) \in \mathcal{R}(\mathtt{v})$. By assumption $vb(\mathtt{a})$ is a variable bound for $\mathtt{a}$ on ρ. Let $j \in \Theta(t, \rho)$. We have that $\mathtt{a} \in \mathtt{def}(l_j)$ by *well-definedness* of $\Delta\mathcal{P}$. Thus $\sigma_j(\mathtt{a}) \leq vb(\mathtt{a})$.

(4) Let $(t, \mathtt{a}, \mathtt{c}) \in \mathcal{R}(\mathtt{v})$. We have $\sum_{j \in \Theta(t,\rho)} vb(\mathtt{a}) + \mathtt{c} = |\Theta(t, \rho)| \times (vb(\mathtt{a}) + \mathtt{c})$.
Further $|\Theta(t, \rho)| = \sharp(t, \rho)$ (Definition 7).

With A) and B) we have

$$\sharp(\tau, \rho) \leq \left(\sum_{(t,\mathtt{c}) \in \mathcal{I}(\mathtt{v})} \sharp(t, \rho) \times \mathtt{c} \right) + \sum_{(t,\mathtt{a},\mathtt{c}) \in \mathcal{R}(\mathtt{v})} \sharp(t, \rho) \times (vb(\mathtt{a}) + \mathtt{c}).$$

□

Lemma A.3 states that the value of a variable $\mathtt{v} \in \mathcal{V}$ on a run ρ of $\Delta\mathcal{P}$ is limited by the maximum over all values to which $\mathtt{v}$ is reset on ρ plus the total amount by which $\mathtt{v}$ is incremented on ρ.

Lemma A.3 *Let* $\mathtt{v} \in \mathcal{V}$*. Let* ρ *be a run of* $\Delta\mathcal{P}$*. Let* $vb : \mathcal{A} \to \mathbb{Z}$ *be s.t.* $vb(\mathtt{a})$ *is a* variable bound *for* $\mathtt{a}$ *on* ρ *for all* $(\_, \mathtt{a}, \_) \in \mathcal{R}(\mathtt{v})$*. Then*

$$\max_{(\_,\mathtt{a},\mathtt{c})\in\mathcal{R}(\mathtt{v})} (vb(\mathtt{a}) + \mathtt{c}) + \sum_{(\tau,\mathtt{c})\in\mathcal{I}(\mathtt{v})} \sharp(\tau, \rho) \times \mathtt{c}$$

is a variable bound *for* $\mathtt{v}$ *on* ρ*.*

Proof We have to show that

$$\sigma_i(\mathtt{v}) \leq \max_{(\_,\mathtt{a},\mathtt{c})\in\mathcal{R}(\mathtt{v})} (vb(\mathtt{a}) + \mathtt{c}) + \sum_{(\tau,\mathtt{c})\in\mathcal{I}(\mathtt{v})} \sharp(\tau, \rho) \times \mathtt{c}$$

holds for all $0 \leq i \leq len(\rho)$ with $\mathtt{v} \in \mathtt{def}(l_i)$.

Let $0 \leq i \leq len(\rho)$ be s.t. $\mathtt{v} \in \mathtt{def}(l_i)$. By *well-definedness* of $\Delta\mathcal{P}$ there is a $0 \leq j < i$, a $b \in \mathcal{A}$ and a $c \in \mathbb{Z}$ s.t. $(\rho(j), b, c) \in \mathcal{R}(\mathtt{v})$ and $\mathtt{v}$ is not reset on $\rho_{[j+1,i]}$, i.e., for all $j < k < i$ $(\rho(k), \_, \_) \notin \mathcal{R}(\mathtt{v})$. In other words: there is a maximal index $j < i$ such that $\mathtt{v}$ is reset on $\rho(j)$. We have:

$$\begin{aligned}
\sigma_i(\mathtt{v}) &\overset{(1)}{\leq} \sigma_{j+1}(\mathtt{v}) + \sum_{(\tau,\mathtt{c})\in\mathcal{I}(\mathtt{v})} \sharp(\tau, \rho_{[j+1,i]}) \times \mathtt{c} \\
&\overset{(2)}{\leq} \sigma_{j+1}(\mathtt{v}) + \sum_{(\tau,\mathtt{c})\in\mathcal{I}(\mathtt{v})} \sharp(\tau, \rho) \times \mathtt{c} \\
&\overset{(3)}{\leq} \sigma_j(b) + c + \sum_{(\tau,\mathtt{c})\in\mathcal{I}(\mathtt{v})} \sharp(\tau, \rho) \times \mathtt{c} \\
&\overset{(4)}{\leq} vb(b) + c + \sum_{(\tau,\mathtt{c})\in\mathcal{I}(\mathtt{v})} \sharp(\tau, \rho) \times \mathtt{c} \\
&\overset{(5)}{\leq} \max_{(_,\mathtt{a},\mathtt{c})\in\mathcal{R}(\mathtt{v})} (vb(\mathtt{a}) + \mathtt{c}) + \sum_{(\tau,\mathtt{c})\in\mathcal{I}(\mathtt{v})} \sharp(\tau, \rho) \times \mathtt{c}
\end{aligned}$$

(1) We have that $\mathtt{v}$ is not reset on $\rho_{[j+1,i]}$. If $\mathtt{v}$ is incremented on $\rho_{[j+1,i]}$ there are indices $j < k < i$ s.t. $(\rho(k), \_) \in \mathcal{I}(\mathtt{v})$. Let $(\tau, \mathtt{c}) \in \mathcal{I}(\mathtt{v})$. An execution of τ can increase the value of $\mathtt{v}$ by at most $\mathtt{c}$ (Definition 18). Therefore the total number $\sharp(\tau, \rho_{[j+1,i]})$ of executions of τ on $\rho_{[j+1,i]}$ adds at most $\sharp(\tau, \rho_{[j+1,i]}) \times \mathtt{c}$ to $\mathtt{v}$. Thus in total $\mathtt{v}$ cannot be increased by more than $\sum_{(\tau,\mathtt{c})\in\mathcal{I}(\mathtt{v})} \sharp(\tau, \rho_{[j+1,i]}) \times \mathtt{c}$ on ρ.
(2) $\sharp(\tau, \rho_{[j+1,i]}) \leq \sharp(\tau, \rho)$. Further for all $(\_, \mathtt{c}) \in \mathcal{I}(\mathtt{v})$ $\mathtt{c} \geq 0$ (Definition 18).
(3) $\sigma_{j+1}(\mathtt{v}) \leq \sigma_j(b) + c$ (Definition 12).
(4) With $(\rho(j), b, c) \in \mathcal{R}(\mathtt{v})$ we have by assumption that $vb(b)$ is a variable bound for b on ρ. Further $b \in \mathtt{def}(l_j)$ by *well-definedness* of $\Delta\mathcal{P}$. Thus $\sigma_j(b) \leq vb(b)$.
(5) We have $(\rho(j), b, c) \in \mathcal{R}(\mathtt{v})$. Therefore $vb(b) + c \leq \max_{(\_,\mathtt{a},\mathtt{c})\in\mathcal{R}(\mathtt{v})} (vb(\mathtt{a}) + \mathtt{c})$. □

A.1.1 Proof of Theorem 1

We show the more general claim formulated in Theorem A.1.

Theorem A.1 *Let $\Delta\mathcal{P}(L, E, l_b, l_e)$ be a well-defined and fan-in free DCP over atoms $\mathcal{A}$. Let Ξ be a set of runs of $\Delta\mathcal{P}$ closed under* normalization. *Let $\zeta : E \mapsto Expr(\mathcal{A})$ be a* local bound mapping *for all $\rho \in \Xi$. Let $T\mathcal{B}$ and $V\mathcal{B}$ be defined as in Definition 19. Let $\mathtt{a} \in \mathcal{A}$ and $\tau \in E$. Let $\rho \in \Xi$. Let σ_0 be the initial state of ρ. We have: (I) $[\![T\mathcal{B}(\tau)]\!](\sigma_0)$ is a* transition bound *for τ on ρ. (II) $[\![V\mathcal{B}(\mathtt{a})]\!](\sigma_0)$ is a* variable bound *for $\mathtt{a}$ on ρ.*

Proof Let $\rho = (\sigma_0, l_0) \xrightarrow{u_0} (\sigma_1, l_1) \xrightarrow{u_1} \cdots \in \Xi$.

If $[\![T\mathcal{B}(\tau)]\!] = \infty$ (I) holds trivially. If $[\![V\mathcal{B}(\mathtt{a})]\!] = \infty$ (II) holds trivially.

Assume $[\![T\mathcal{B}(\tau)]\!] \neq \infty$ and $[\![V\mathcal{B}(\mathtt{a})]\!] \neq \infty$. Then in particular the computation of $T\mathcal{B}(\tau)$ resp. $V\mathcal{B}(\mathtt{a})$ terminates. We proceed by induction over the call tree of $T\mathcal{B}(\tau)$ resp. $V\mathcal{B}(\mathtt{a})$.

Base Case:

(I) No function call is triggered when computing $V\mathcal{B}(\mathtt{a})$. This is the case iff $\mathtt{a} \in \mathcal{C}$ (Definition 19). Then $V\mathcal{B}(\mathtt{a}) = \mathtt{a}$ and the claim holds trivially with $\mathtt{a} \in \mathcal{C}$ (Definition 13).

(II) No function call is triggered when computing $T\mathcal{B}(\tau)$. This is the case iff $\zeta(\tau) \notin \mathcal{V}$ (Definition 19). Then $[\![T\mathcal{B}(\tau)]\!](\sigma_0) = [\![\zeta(\tau)]\!](\sigma_0)$ is a *transition bound* for τ on ρ by Definition 17.

Step Case:

(I) $\mathtt{a} \notin \mathcal{C}$, thus $\mathtt{a} \in \mathcal{V}$. Let $\mathtt{v} = \mathtt{a}$. Let $0 \leq i \leq len(\rho)$ be s.t. $\mathtt{v} \in \mathtt{def}(l_i)$. We have:

$$\begin{aligned}
\sigma_i(\mathtt{v}) &\overset{(1)}{\leq} \max_{(_,b,\mathtt{c}) \in \mathcal{R}(\mathtt{v})} ([\![V\mathcal{B}(b)]\!](\sigma_0) + \mathtt{c}) + \sum_{(t,\mathtt{c}) \in \mathcal{I}(\mathtt{v})} \sharp(t, \rho) \times \mathtt{c} \\
&\overset{(2)}{\leq} \max_{(_,b,\mathtt{c}) \in \mathcal{R}(\mathtt{v})} ([\![V\mathcal{B}(b)]\!](\sigma_0) + \mathtt{c}) + \sum_{(t,\mathtt{c}) \in \mathcal{I}(\mathtt{v})} [\![T\mathcal{B}(t)]\!](\sigma_0) \times \mathtt{c} \\
&\overset{(3)}{=} [\![\max_{(_,b,\mathtt{c}) \in \mathcal{R}(\mathtt{v})} (V\mathcal{B}(b) + \mathtt{c})]\!](\sigma_0) + [\![\mathtt{Incr}(\mathtt{v})]\!](\sigma_0) \\
&\overset{(4)}{=} [\![V\mathcal{B}(\mathtt{v})]\!](\sigma_0)
\end{aligned}$$

(1) By Lemma A.3: Let $(\_, b, \_) \in \mathcal{R}(\mathtt{v})$. We have that $V\mathcal{B}(b)$ is recursively called when computing $V\mathcal{B}(\mathtt{v})$ (Definition 19). Note that with $[\![V\mathcal{B}(\mathtt{v})]\!] \neq \infty$ also $[\![V\mathcal{B}(b)]\!] \neq \infty$. By I.H. $[\![V\mathcal{B}(b)]\!](\sigma_0)$ is a *variable bound* for b on ρ.

(2) Let $(t, \_) \in \mathcal{I}(\mathtt{v})$. We have that $T\mathcal{B}(t)$ is called when computing $V\mathcal{B}(\mathtt{v})$ (Definition 19). Note that with $[\![V\mathcal{B}(\mathtt{v})]\!] \neq \infty$ also $[\![T\mathcal{B}(t)]\!] \neq \infty$. By I.H. $\sharp(t, \rho) \leq [\![T\mathcal{B}(t))]\!](\sigma_0)$. We thus get $\sum_{(t,\mathtt{c}) \in \mathcal{I}(\mathtt{v})} \sharp(t, \rho) \times \mathtt{c} \leq \sum_{(t,\mathtt{c}) \in \mathcal{I}(\mathtt{v})} [\![T\mathcal{B}(t)]\!](\sigma_0) \times \mathtt{c}$ because for all $(\_, \mathtt{c}) \in \mathcal{I}(\mathtt{v})$ we have $\mathtt{c} > 0$ (Definition 18).

(3) $[\![\mathtt{Incr}(\mathtt{v})]\!](\sigma_0) = \sum_{(t,\mathtt{c}) \in \mathcal{I}(\mathtt{v})} [\![T\mathcal{B}(t)]\!](\sigma_0) \times \mathtt{c}$ (Definition 19 and Definition 15).

(4) Definition 19 and Definition 15.

(II) $\zeta(\tau) \in \mathcal{V}$. We have:

$$\begin{aligned}
\sharp(\tau,\rho) &\overset{(1)}{\leq} (\sum_{(t,\mathtt{c})\in\mathcal{I}(\zeta(\tau))} \sharp(t,\rho)\times \mathtt{c}) + \sum_{(t,b,c)\in\mathcal{R}(\zeta(\tau))} \sharp(t,\rho)\times [\![V\mathcal{B}(b)]\!](\sigma_0) + c \\
&\overset{(2)}{\leq} \sum_{(t,\mathtt{c})\in\mathcal{I}(\zeta(\tau))} [\![T\mathcal{B}(t)]\!](\sigma_0)\times \mathtt{c} + \sum_{(t,b,c)\in\mathcal{R}(\zeta(\tau))} \sharp(t,\rho)\times [\![V\mathcal{B}(b)]\!](\sigma_0) + c \\
&\overset{(3)}{=} [\![\mathtt{Incr}(\zeta(\tau))]\!](\sigma_0) + \sum_{(t,b,c)\in\mathcal{R}(\zeta(\tau))} \sharp(t,\rho)\times [\![V\mathcal{B}(b)]\!](\sigma_0) + c \\
&\overset{(4)}{\leq} [\![\mathtt{Incr}(\zeta(\tau))]\!](\sigma_0) + \sum_{(t,b,c)\in\mathcal{R}(\zeta(\tau))} [\![T\mathcal{B}(t)]\!](\sigma_0)\times \max([\![V\mathcal{B}(b)]\!](\sigma_0)+c,0) \\
&\overset{(5)}{=} [\![T\mathcal{B}(\tau)]\!](\sigma_0)
\end{aligned}$$

(1) By Lemma A.2: Since $\varXi$ is closed under *normalization* we have that $\zeta(\tau)$ is a *local transition bound* for τ on $\lfloor\rho\rfloor$. Further: Let $(\_,b,\_) \in \mathcal{R}(\zeta(\tau))$. We have that $V\mathcal{B}(b)$ is called during the computation of $T\mathcal{B}(\tau)$ (Definition 19). Note that with $[\![T\mathcal{B}(\tau)]\!] \neq \infty$ also $[\![V\mathcal{B}(b)]\!] \neq \infty$. By I.H. $[\![V\mathcal{B}(b)]\!](\sigma_0)$ is a *variable bound* for b.
(2) Let $(t,\_) \in \mathcal{I}(\zeta(\tau))$. We have that there is a recursive call to $T\mathcal{B}(t)$ during the computation of $T\mathcal{B}(\tau)$ (Definition 19). Note that with $[\![T\mathcal{B}(\tau)]\!] \neq \infty$ also $[\![T\mathcal{B}(t)]\!] \neq \infty$. By I.H. $\sharp(t,\rho) \leq [\![T\mathcal{B}(t)]\!](\sigma_0)$. Further for all $(\_,\mathtt{c}) \in \mathcal{I}(\mathtt{v})$ $\mathtt{c} \geq 0$ (Definition 18).
(3) Definition 19 and Definition 15.
(4) Let $(t,\_,\_) \in \mathcal{R}(\zeta(\tau))$. We have that $T\mathcal{B}(t)$ is recursively called during the computation of $T\mathcal{B}(\tau)$ (Definition 19). Note that with $[\![T\mathcal{B}(\tau)]\!] \neq \infty$ also $[\![T\mathcal{B}(t)]\!] \neq \infty$. By I.H. $\sharp(t,\rho) \leq [\![T\mathcal{B}(t)]\!](\sigma_0)$.
(5) Definition 19 and Definition 15. □

A.2 Soundness of Reasoning Based on Reset Chains

Lemma A.7 extends Lemma A.2 by chained resets. Lemma A.4, Lemma A.5 and Lemma A.6 are helper lemmas needed for the proof of Lemma A.7.

Definition A.3 (Matching of a Reset Chain) Let $\kappa = \mathtt{a}_n \xrightarrow{\tau_n,\mathtt{c}_n} \mathtt{a}_{n-1} \xrightarrow{\tau_{n-1},\mathtt{c}_{n-1}} \cdots \mathtt{a}_0$ be a *reset chain* of $\Delta\mathcal{P}$. Let ρ be a *run* of $\Delta\mathcal{P}$. We call $i_n, i_{n-1} \dots i_1 \in \mathbb{N}$ with $0 \leq i_n < i_{n-1} \cdots < i_1 < \mathit{len}(\rho)$ a *matching of* κ on ρ iff $\rho(i_j) = \tau_j$ holds for all $n \geq j \geq 1$. We call i_n the first index and i_1 is the last index. A matching $i_n, i_{n-1}, \dots, i_1$ of κ on ρ is precise iff for all $n > j \geq 1$ it holds that a_j is *not reset* on $\rho_{[i_{j+1}+1,i_j]}$, i.e., $(\rho(k),\_,\_) \notin \mathcal{R}(\mathtt{a}_j)$ for all $i_{j+1} < k < i_j$.

Informally: There is a matching of $\kappa = \mathtt{a}_n \xrightarrow{\tau_n,\mathtt{c}_n} \mathtt{a}_{n-1} \xrightarrow{\tau_{n-1},\mathtt{c}_{n-1}} \cdots \mathtt{a}_0$ on a run ρ if ρ contains the transitions $\tau_n, \tau_{n-1}, \dots, \tau_1$ in that order. A matching $i_n, i_{n-1}, \dots i_1$ is precise if for all $n > j \geq 1$ it holds that a_j flows into a_{j-1} when executing $\rho(i_j)$ because a_j is not reset between the reset of a_j to a_{j+1} on $\rho(i_{j+1})$ and the reset of a_{j-1} to a_j on $\rho(i_j)$.

Definition A.4 (First- and Last-Indices of Precise Matchings) Let ρ be a run of $\Delta\mathcal{P}$. Let $\kappa = \mathtt{a}_n \xrightarrow{\tau_n,c_n} \mathtt{a}_{n-1} \xrightarrow{\tau_n,c_n} \dots \xrightarrow{\tau_1,c_1} \mathtt{a}_0$ be a reset chain. We define

$\alpha(\kappa, \rho)$ to denote the set
$\{(i_n, i_1) \mid \exists i_{n-1}, \ldots, i_2 \text{ s.t. } i_n, i_{n-1}, i_{n-2}, \ldots, i_2, i_1 \text{ is a precise matching of } \kappa \text{ on } \rho\}$.

I.e., $\alpha(\kappa, \rho)$ is the set of first- and last-indices of all precise matchings of κ on ρ. Note that in particular $i \leq j$ for all $(i, j) \in \alpha(\kappa, \rho)$, i.e., the interval $[i \ldots j]$ is non-empty.

Given a reset chain κ from b to $\mathtt{v}$ and a precise matching of κ on a run ρ with first index i and last index j, Lemma A.4 states that the value of $\mathtt{v}$ in state σ_j on ρ is bounded by the value of b in state σ_i on ρ and the increments of $\mathtt{a} \in \mathit{atm}(\kappa)$ between index i and index j on ρ.

Lemma A.4 *Let ρ be a run of $\Delta\mathcal{P}$. Let $b \in \mathcal{A}$ and $\mathtt{v} \in \mathcal{V}$. Let κ be a reset chain from b to $\mathtt{v}$. Let $(i, j) \in \alpha(\kappa, \rho)$. Then*

$$\sigma_{j+1}(\mathtt{v}) \leq \sigma_i(b) + c(\kappa) + \sum_{\mathtt{a} \in \mathit{atm}(\kappa) \setminus \{\mathtt{v}\}} \sum_{(\tau, \mathtt{c}) \in \mathcal{I}(\mathtt{a})} \sharp(\tau, \rho_{[i+1, j]}) \times \mathtt{c}$$

holds.

Proof We show the claim by induction on the length of κ.

Base Case: Let $\kappa = b \xrightarrow{\tau, \mathtt{c}} \mathtt{v}$. With $(i, j) \in \alpha(\kappa, \rho)$ we have that $i = j$ and $\rho(i) = \rho(j) = \tau$. Further we have that $(\tau, b, \mathtt{c}) \in \mathcal{R}(\mathtt{v})$ (Definition 20). Thus $\sigma_{j+1}(\mathtt{v}) = \sigma_{i+1}(\mathtt{v}) \leq \sigma_i(b) + \mathtt{c}$ (Definition 18). Note that $\mathit{atm}(\kappa) \setminus \{\mathtt{v}\} = \emptyset$ since $b \notin \mathit{atm}(\kappa)$ (Definition 20).

Step Case: Let $\kappa = \mathtt{a}_{n+1} \xrightarrow{\tau_{n+1}, c_{n+1}} \mathtt{a}_n \xrightarrow{\tau_n, c_n} \ldots \xrightarrow{\tau_1, c_1} \mathtt{v}$ with $\mathtt{a}_{n+1} = b$. Let $i_{n+1}, i_n, i_{n-1}, \ldots, i_1$ be a precise matching of κ on ρ with $i_{n+1} = i$ and $i_1 = j$.

$$\begin{aligned}
\sigma_{j+1}(\mathtt{v}) = \sigma_{i_1+1}(\mathtt{v}) &\overset{(1)}{\leq} \sigma_{i_n}(\mathtt{a}_n) + c(\kappa_{[n,0]}) + \sum_{\mathtt{a} \in \mathit{atm}(\kappa_{[n,0]}) \setminus \{\mathtt{v}\}} \sum_{(\tau, \mathtt{c}) \in \mathcal{I}(\mathtt{a})} \sharp(\tau, \rho_{[i_n+1, i_1]}) \times \mathtt{c} \\
&\overset{(2)}{\leq} \sigma_{i_{n+1}+1}(\mathtt{a}_n) + \Big(\sum_{(\tau, \mathtt{c}) \in \mathcal{I}(\mathtt{a}_n)} \sharp(\tau, \rho_{[i_{n+1}+1, i_n]}) \times \mathtt{c}\Big) \\
&\quad + c(\kappa_{[n,0]}) + \sum_{\mathtt{a} \in \mathit{atm}(\kappa_{[n,0]}) \setminus \{\mathtt{v}\}} \sum_{(\tau, \mathtt{c}) \in \mathcal{I}(\mathtt{a})} \sharp(\tau, \rho_{[i_n+1, i_1]}) \times \mathtt{c} \\
&\overset{(3)}{\leq} \sigma_{i_{n+1}}(\mathtt{a}_{n+1}) + c_{n+1} + \Big(\sum_{(\tau, \mathtt{c}) \in \mathcal{I}(\mathtt{a}_n)} \sharp(\tau, \rho_{[i_{n+1}+1, i_n]}) \times \mathtt{c}\Big) \\
&\quad + c(\kappa_{[n,0]}) + \sum_{\mathtt{a} \in \mathit{atm}(\kappa_{[n,0]}) \setminus \{\mathtt{v}\}} \sum_{(\tau, \mathtt{c}) \in \mathcal{I}(\mathtt{a})} \sharp(\tau, \rho_{[i_n+1, i_1]}) \times \mathtt{c} \\
&\overset{(4)}{\leq} \sigma_{i_{n+1}}(\mathtt{a}_{n+1}) + c_{n+1} + \Big(\sum_{(\tau, \mathtt{c}) \in \mathcal{I}(\mathtt{a}_n)} \sharp(\tau, \rho_{[i_{n+1}+1, i_1]}) \times \mathtt{c}\Big) \\
&\quad + c(\kappa_{[n,0]}) + \sum_{\mathtt{a} \in \mathit{atm}(\kappa_{[n,0]}) \setminus \{\mathtt{v}\}} \sum_{(\tau, \mathtt{c}) \in \mathcal{I}(\mathtt{a})} \sharp(\tau, \rho_{[i_{n+1}+1, i_1]}) \times \mathtt{c} \\
&\overset{(5)}{=} \sigma_{i_{n+1}}(\mathtt{a}_{n+1}) + c(\kappa) + \sum_{\mathtt{a} \in \mathit{atm}(\kappa) \setminus \{\mathtt{v}\}} \sum_{(\tau, \mathtt{c}) \in \mathcal{I}(\mathtt{a})} \sharp(\tau, \rho_{[i_{n+1}+1, i_1]}) \times \mathtt{c}
\end{aligned}$$

(1) By I.H.: We have that $\kappa_{[n,0]}$ is also a reset chain (Definition 20, note that $\kappa_{[n,0]}$ is non-empty by definition of κ) and since $i_{n+1}, i_n, \ldots, i_1$ is a precise matching of κ on ρ, $i_n, \ldots, i_1$ is a precise matching of $\kappa_{[n,0]}$ on ρ (Definition A.3).

(2) We have that for all $i_{n+1} < j < i_n$ $(\rho(j), \_, \_) \notin \mathcal{R}(\mathtt{a}_n)$ (Definition A.3), i.e., $\mathtt{a}_n$ is not reset on $\rho_{[i_{n+1}+1, i_n]}$. In the proof of Lemma A.3 we show that $\sigma_{i_n}(\mathtt{a}_n) \leq \sigma_{i_{n+1}+1}(\mathtt{a}_n) + \sum_{(\tau,\mathtt{c}) \in \mathcal{I}(\mathtt{a}_n)} \sharp(\tau, \rho_{[i_{n+1}+1, i_n]}) \times \mathtt{c}$.

(3) $\sigma_{i_{n+1}+1}(\mathtt{a}_n) \leq \sigma_{i_n+1}(\mathtt{a}_{n+1}) + \mathtt{c}_{n+1}$ (Definition 20)

(4) Note that $[i_{n+1}+1 \dots i_n]$ is a sub-interval of $[i_{n+1}+1 \dots i_1]$. Therefore $\sharp(\tau, \rho_{[i_{n+1}+1, i_n]}) \leq \sharp(\tau, \rho_{[i_{n+1}+1, i_1]})$ for all $\tau \in E$. Accordingly $[i_n + 1 \dots i_1]$ is a sub-interval of $[i_{n+1}+1 \dots i_1]$. Therefore $\sharp(\tau, \rho_{[i_n+1, i_1]}) \leq \sharp(\tau, \rho_{[i_{n+1}+1, i_1]})$ for all $\tau \in E$. We thus get $\sum_{(\tau,\mathtt{c}) \in \mathcal{I}(\mathtt{a})} \sharp(\tau, \rho_{[i_{n+1}+1, i_n]}) \times \mathtt{c} \leq \sum_{(\tau,\mathtt{c}) \in \mathcal{I}(\mathtt{a})} \sharp(\tau, \rho_{[i_{n+1}+1, i_1]}) \times \mathtt{c}$ and $\sum_{(\tau,\mathtt{c}) \in \mathcal{I}(\mathtt{a})} \sharp(\tau, \rho_{[i_n+1, i_1]}) \times \mathtt{c} \leq \sum_{(\tau,\mathtt{c}) \in \mathcal{I}(\mathtt{a})} \sharp(\tau, \rho_{[i_{n+1}+1, i_1]}) \times \mathtt{c}$ because we have that for all $\mathtt{v} \in \mathcal{V}$ and for all $(\_, \mathtt{c}) \in \mathcal{I}(\mathtt{v})$ it holds that $\mathtt{c} > 0$ (Definition 18).

(5) We have $c(\kappa) = c(\kappa_{[n,0]}) + \mathtt{c}_{n+1}$ and $atm(\kappa) = atm(\kappa_{[n,0]}) \cup \{\mathtt{a}_n\}$ (Definition 20). □

Let $\mathtt{v} \in \mathcal{V}$. Lemma A.5 states that for each index j on a run ρ s.t. $\mathtt{v}$ is reset on $\rho(j)$, there is a corresponding *optimal* reset chain κ and a precise matching of κ on ρ ending at j.

Lemma A.5 *Let ρ be a run of $\Delta\mathcal{P}$. Let $\mathtt{v} \in \mathcal{V}$. Let $(\tau, \_, \_) \in \mathcal{R}(\mathtt{v})$. Let j be s.t. $\rho(j) = \tau$. There is a $\kappa \in \mathfrak{R}(\mathtt{v})$ and a $i \leq j$ s.t. $(i, j) \in \alpha(\kappa, \rho)$.*

Proof Let $\mathtt{a} \in \mathcal{A}$ and $\mathtt{c} \in \mathbb{Z}$ be such that $(\tau, \mathtt{a}, \mathtt{c}) \in \mathcal{R}(\mathtt{v})$ (note that by *determinism* of $\Delta\mathcal{P}$ there is exactly one such $\mathtt{a}$ and $\mathtt{c}$). We proof the claim by the following recursive reasoning:

[Start] We show that there is a *sound* reset chain κ that ends at $\mathtt{v}$ and a precise matching of κ on ρ that ends at j: Obviously $\mathtt{a} \xrightarrow{\tau,\mathtt{c}} \mathtt{v}$ is a reset chain. Further $\mathtt{a} \xrightarrow{\tau,\mathtt{c}} \mathtt{v}$ is trivially *sound* (Definition 20). We have that j is a *precise* matching for $\mathtt{a} \xrightarrow{\tau,\mathtt{c}} \mathtt{v}$ on ρ because by assumption $\rho(j) = \tau$ (Definition A.3).
[Recursive Step] We thus have that there is a *sound* reset chain $\kappa = \mathtt{a}_n \xrightarrow{\tau_n,\mathtt{c}_n} \mathtt{a}_{n-1} \xrightarrow{\tau_{n-1},\mathtt{c}_{n-1}} \dots \xrightarrow{\tau_1,\mathtt{c}_1} \mathtt{v}$ and a precise matching $i_n, i_{n-1}, \dots, i_1$ of κ on ρ with $i_1 = j$. If κ is *optimal* then $\kappa \in \mathfrak{R}(\mathtt{v})$ (Definition 20) and with $(i_n, j) \in \alpha(\kappa, \rho)$ the claim is proven. Assume κ is *not* optimal. Then $\mathtt{a}_n \in \mathcal{V}$ because κ is not maximal (Definition 20). By well-definedness of $\Delta\mathcal{P}$ $\mathtt{a}_n$ is *reset* on $\rho_{[0,i_n]}$, i.e., there is a $0 \leq k < i_n$ s.t. $(\rho(k), \_, \_) \in \mathcal{R}(\mathtt{a}_n)$. Let i_{n+1} denote the maximal such k. Let $\tau_{n+1} = \rho(i_{n+1})$. Let $\mathtt{a}_{n+1} \in \mathcal{A}$ and $\mathtt{c}_{n+1} \in \mathbb{Z}$ be s.t. $(\tau_{n+1}, \mathtt{a}_{n+1}, \mathtt{c}_{n+1}) \in \mathcal{R}(\mathtt{a}_n)$. Then $\varkappa = a_{n+1} \xrightarrow{\tau_{n+1},\mathtt{c}_{n+1}} \mathtt{a}_n \xrightarrow{\tau_n,\mathtt{c}_n} \mathtt{a}_{n-1} \dots \mathtt{v}$ is a *reset chain* ending in $\mathtt{v}$ and $i_{n+1}, i_n, \dots, i_1$ is a *precise matching* of $\varkappa$ on ρ (Definition A.3). We show that $\varkappa$ is *sound*: First note that $\varkappa_{[n,0]} = \kappa$ and because κ is *sound* we have that for all $1 \leq i < n$ it holds that $\mathtt{a}_i$ *is reset on all paths from the target location of* τ_1 *to the source location of* τ_i. It remains to show that this also holds for $\mathtt{a}_n$. Since κ is not optimal there is a *sound* reset chain that extends κ (Definition 20). Now, because $\mathtt{a}_n$ is on that extended *sound* reset chain we have that also $\mathtt{a}_n$ *is reset on all paths from the target location of* τ_1 *to the source location of* τ_n (Definition 20). We conclude that $\varkappa$ is *sound*. We can thus recursively apply our reasoning on $\varkappa$.
[Termination] Since by assumption the reset graph is *acyclic* and its node set $\mathcal{A}$ is *finite*, a *optimal* reset chain $\kappa \in \mathfrak{R}(\mathtt{v})$ and a matching of κ that ends at j is constructed by iterating the stated reasoning *finitely* often. □

Note that with Lemma A.4 and Lemma A.5 we can bound the value to which $\mathtt{v}$ is reset at index j in terms of the value of $in(\kappa)$ at index i, where i is the start-index of the matching that ends at j.

Lemma A.6 states that precise matchings of *optimal* reset chains that share a common suffix never overlap.

Lemma A.6 *Let ρ be a run of $\Delta\mathcal{P}$. Let $\mathtt{v} \in \mathcal{V}$. Let $\kappa, \varkappa \in \mathfrak{R}(\mathtt{v})$ be s.t. κ and $\varkappa$ have a common suffix, i.e., there exists $l > 0$ s.t. $\kappa_{[l,0]} = \varkappa_{[l,0]}$. Let $(i_k, i_1) \in \alpha(\kappa, \rho)$ and $(j_n, j_1) \in \alpha(\varkappa, \rho)$. Either $\kappa = \varkappa$ and $[i_k \dots i_1] = [j_n \dots j_1]$ or the two intervals $[i_k \dots i_1]$ and $[j_n \dots j_1]$ are* disjoint*, i.e., $i_1 < j_n$ or $j_1 < i_k$.*

Proof Let $\kappa = \mathtt{a}_k \xrightarrow{\tau_k, \mathtt{c}_k} \mathtt{a}_{k-1} \dots \mathtt{a}_1 \xrightarrow{\tau_1, \mathtt{c}_1} \mathtt{v}$. Let $\varkappa = b_n \xrightarrow{t_n, \mathtt{c}_n} b_{n-1} \dots b_1 \xrightarrow{t_1, \mathtt{c}_1} \mathtt{v}$.
Let $i_k, i_{k-1}, \dots i_1$ be a *precise* matching of κ on ρ.
Let $j_n, j_{n-1}, \dots j_1$ be a *precise* matching of $\varkappa$ on ρ.
[A] We show that if $i_1 = j_1$ then $i_k = j_n$ and $\kappa = \varkappa$: W.l.o.g. assume $k \leq n$.
[A.1] We show that for all $k \leq l \leq 1$ $i_l = j_l$: By assumption $i_1 = i_1 = j_1 = j_1$. We conclude that $\mathtt{a}_1 = b_1$ because since $\Delta\mathcal{P}$ is *fan-in free* there is exactly one $\mathtt{a}_1$ s.t. $(\mathtt{a}_1, \_, \rho(i_1)) \in \mathcal{R}(\mathtt{v})$. Assume $i_2 \neq j_2$. Case $j_2 < i_2$: By Definition A.3 $\mathtt{a}_1$ is *not reset* on $\rho_{[j_2+1, j_1]}$, i.e., $(\rho(k), \_\_) \notin \mathcal{R}(\mathtt{a}_1)$ for all $j_2 < k < j_1$. Note that $j_2 < i_2 < i_1 = j_1$. We have $(\rho(i_2), \_, \_) \in \mathcal{R}(b_1)$ (Definition A.3 and Definition 20). With $\mathtt{a}_1 = b_1$ we have $(\rho(i_2), \_, \_) \in \mathcal{R}(\mathtt{a}_1)$. Contradiction. Case $i_2 < j_2$: Analogous. Thus $i_2 = j_2$. We apply the same reasoning for $i_3, i_4 \dots i_k$ consecutively.
[A.2] We show that $k = n$: By [A.1] we have that $\varkappa_{[k,1]} = \kappa$ (Definition A.3). Thus κ is a *suffix* of $\varkappa$. But by assumption κ is *optimal*. Thus $\kappa = \varkappa$ (Definition 20).
[A] is proven with [A.1] and [A.2].
[B] We show that if $i_1 \neq j_1$ then $i_1 < j_n$ or $j_1 < i_k$, i.e., the intervals $[i_k \dots i_1]$ and $[j_n \dots j_1]$ are *disjoint*:
[B.1] We have $\rho(i_1) = \rho(j_1) = t_1$ because by assumption κ and $\varkappa$ have a common suffix.
[B.2] We show [B.2.i] that for all l with $j_n \leq l < j_1$ it holds that $\rho(l) \neq t_1$ and [B.2.ii] that for all l with $i_k \leq l < i_1$ it holds that $\rho(l) \neq t_1$.
[B.2.i] Assume there is some l with $j_n \leq l < j_1$ s.t. $\rho(l) = t_1$. Then there is some $n \geq r > 1$ s.t. $j_r \leq l < j_{r-1}$. Since $j_n, j_{n-1}, \dots j_1$ is a *precise* matching of $\varkappa$ we have that for all $j_r < s < j_{r-1}$ $(\rho(s), \_, \_) \notin \mathcal{R}(\mathtt{a}_{r-1})$ (Definition A.3). But since $\varkappa$ is *sound* $\mathtt{a}_{r-1}$ *must be reset* on *all paths from the target location of* t_1 *to the source location of* t_{r-1}, i.e., in particular on $\rho_{[l+1, j_{r-1}]}$ because $\rho(l) = t_1$ and $\rho(j_{r-1}) = t_{r-1}$ (Definition A.3). Thus there must be some s with $j_r \leq l < s < j_{r-1}$ s.t. $(\rho(s), \_, \_) \in \mathcal{R}(\mathtt{a}_{r-1})$. Contradiction.
[B.2.ii] Analogous.
[B.1] and [B.2] imply [B]: By assumption $i_1 \neq j_1$. W.l.o.g. let $i_1 < j_1$. With $i_k \leq i_1$ and $j_n \leq j_1$ we have $i_k < j_1$. We thus have to show that $i_1 < j_n$: Assume $j_n \leq i_1$: Then $j_n \leq i_1 < j_1$. But with [B.1] this contradicts [B.2]. Therefore $i_1 < j_n$.
With [A] and [B] the claim is proven. □

Lemma A.7 extends Lemma A.2 by chained resets. Let $\mathtt{v}$ be a local bound for τ: The question how often a given transition τ may appear on a run ρ is translated to the question how often the transitions that increase the value of the local bound $\mathtt{v}$ are executed. But in contrast to Lemma A.2 Lemma A.7 takes the context under which these transitions may increase $\mathtt{v}$ into account. See Section 3.3 for more details.

Lemma A.7 *Let* $\rho = (\sigma_0, l_0) \xrightarrow{u_0} (\sigma_1, l_1) \xrightarrow{u_1} \ldots$ *be a run of* $\Delta\mathcal{P}$. *Let* $\tau \in E$. *Let* $\mathtt{v} \in \mathcal{V}$ *be a* local bound *for* τ *on* $\lfloor\rho\rfloor$. *Let* $vb : \mathcal{A} \to \mathbb{Z}$ *be s.t.* $vb(\mathtt{a})$ *is a* variable bound *for* $\mathtt{a}$ *on* ρ *for all* $\mathtt{a} \in \{in(\kappa) \mid \kappa \in \mathfrak{R}(\mathtt{v})\}$. *Then*

$$\left(\sum_{\mathtt{a}\in \bigcup\limits_{\kappa\in\mathfrak{R}(\mathtt{v})} atm_1(\kappa)} \sum_{(t,\mathtt{c})\in\mathcal{I}(\mathtt{a})} \sharp(t,\rho) \times \mathtt{c}\right) + \sum_{\kappa\in\mathfrak{R}(\mathtt{v})} (\min_{t\in trn(\kappa)} \sharp(t,\rho)) \times \max(vb(in(\kappa)) + c(\kappa), 0) + \sum_{\mathtt{a}\in atm_2(\kappa)} \sum_{(t,\mathtt{c})\in\mathcal{I}(\mathtt{a})} \sharp(t,\rho) \times \mathtt{c}$$

is a transition bound *for* τ *on* ρ.

Proof As argued in the proof of Lemma A.2 it is sufficient to consider the case $\rho = \lfloor\rho\rfloor$.

A) As shown in the proof of Lemma A.2 we have that
$\sharp(\tau,\rho) \leq \left(\sum_{(t,\mathtt{c})\in\mathcal{I}(\mathtt{v})} \sharp(t,\rho) \times \mathtt{c}\right) + \sum_{j\in\Theta(\mathcal{R}(\mathtt{v}),\rho)} \sigma_{j+1}(\mathtt{v})$

B) We show that

$$\sum_{j\in\Theta(\mathcal{R}(\mathtt{v}),\rho)} \sigma_{j+1}(\mathtt{v}) \leq \left(\sum_{\mathtt{a}\in \bigcup\limits_{\kappa\in\mathfrak{R}(\mathtt{v})} atm_1(\kappa)\setminus\{\mathtt{v}\}} \sum_{(t,\mathtt{c})\in\mathcal{I}(\mathtt{a})} \sharp(t,\rho) \times \mathtt{c}\right) + \sum_{\kappa\in\mathfrak{R}(\mathtt{v})} (\min_{t\in trn(\kappa)} \sharp(t,\rho)) \times \max(vb(in(\kappa)) + c(\kappa), 0) + \sum_{\mathtt{a}\in atm_2(\kappa)} \sum_{(t,\mathtt{c})\in\mathcal{I}(\mathtt{a})} \sharp(t,\rho) \times \mathtt{c}$$

With Lemma A.5 we have that for each $j \in \Theta(\mathcal{R}(\mathtt{v}),\rho)$ there is *at least* one $\kappa \in \mathfrak{R}(\mathtt{v})$ and one $i \leq j$ s.t. $(i,j) \in \alpha(\kappa,\rho)$.

Further: Let $\kappa \in \mathfrak{R}(\mathtt{v})$. Let $(i,j) \in \alpha(\kappa,\rho)$. With Lemma A.4 we have that:
$\sigma_{j+1}(\mathtt{v}) \leq \sigma_i(in(\kappa)) + c(\kappa) + \sum_{\mathtt{a}\in atm(\kappa)\setminus\{\mathtt{v}\}} \sum_{(t,\mathtt{c})\in\mathcal{I}(\mathtt{a})} \sharp(t,\rho_{[i+1,j]}) \times \mathtt{c}$

Therefore:

$$\sum_{j\in\Theta(\mathcal{R}(\mathtt{v}),\rho)} \sigma_{j+1}(\mathtt{v}) \leq \sum_{\kappa\in\mathfrak{R}(\mathtt{v})} \sum_{(i,j)\in\alpha(\kappa,\rho)} \sigma_i(in(\kappa)) + c(\kappa) + \sum_{\mathtt{a}\in atm(\kappa)\setminus\{\mathtt{v}\}} \sum_{(t,\mathtt{c})\in\mathcal{I}(\mathtt{a})} \sharp(t,\rho_{[i+1,j]}) \times \mathtt{c}$$

$$\stackrel{(1a)}{=} \sum_{\kappa\in\mathfrak{R}(\mathtt{v})} \left(\sum_{(i,j)\in\alpha(\kappa,\rho)} \sigma_i(in(\kappa)) + c(\kappa)\right) + \left(\sum_{(i,j)\in\alpha(\kappa,\rho)} \sum_{\mathtt{a}\in atm(\kappa)\setminus\{\mathtt{v}\}} \sum_{(t,\mathtt{c})\in\mathcal{I}(\mathtt{a})} \sharp(t,\rho_{[i+1,j]}) \times \mathtt{c}\right)$$

$$
\begin{aligned}
&\overset{(1b)}{=} \sum_{\kappa \in \mathfrak{R}(\mathtt{v})} \left(\sum_{(i,j)\in\alpha(\kappa,\rho)\rho} \sigma_i(in(\kappa)) + c(\kappa) \right) \\
&\qquad + \sum_{\mathtt{a}\in atm(\kappa)\setminus\{\mathtt{v}\}} \sum_{(t,\mathtt{c})\in\mathcal{I}(\mathtt{a})} \left(\sum_{(i,j)\in\alpha(\kappa,\rho)} \sharp(t, \rho_{[i+1,j]}) \right) \times \mathsf{c}
\end{aligned}
$$

$$
\begin{aligned}
&\overset{(1)}{=} \sum_{\kappa \in \mathfrak{R}(\mathtt{v})} \left(\sum_{(i,j)\in\alpha(\kappa,\rho)\rho} \sigma_i(in(\kappa)) + c(\kappa) \right) \\
&\qquad + \sum_{\mathtt{a}\in atm_1(\kappa)\setminus\{\mathtt{v}\}} \sum_{(t,\mathtt{c})\in\mathcal{I}(\mathtt{a})} \left(\sum_{(i,j)\in\alpha(\kappa,\rho)} \sharp(t, \rho_{[i+1,j]}) \right) \times \mathsf{c} \\
&\qquad + \sum_{\mathtt{a}\in atm_2(\kappa)} \sum_{(t,\mathtt{c})\in\mathcal{I}(\mathtt{a})} \left(\sum_{(i,j)\in\alpha(\kappa,\rho)} \sharp(t, \rho_{[i+1,j]}) \right) \times \mathsf{c}
\end{aligned}
$$

$$
\begin{aligned}
&\overset{(2)}{\leq} \sum_{\kappa \in \mathfrak{R}(\mathtt{v})} \left(\sum_{(i,j)\in\alpha(\kappa,\rho)} \sigma_i(in(\kappa)) + c(\kappa) \right) \\
&\qquad + \sum_{\mathtt{a}\in atm_1(\kappa)\setminus\{\mathtt{v}\}} \sum_{(t,\mathtt{c})\in\mathcal{I}(\mathtt{a})} \left(\sum_{(i,j)\in\alpha(\kappa,\rho)} \sharp(t, \rho_{[i+1,j]}) \right) \times \mathsf{c} \\
&\qquad + \sum_{\mathtt{a}\in atm_2(\kappa)} \sum_{(t,\mathtt{c})\in\mathcal{I}(\mathtt{a})} \sharp(t, \rho) \times \mathsf{c}
\end{aligned}
$$

$$
\begin{aligned}
&\overset{(3a)}{=} \left(\sum_{\kappa \in \mathfrak{R}(\mathtt{v})} \sum_{\mathtt{a}\in atm_1(\kappa)\setminus\{\mathtt{v}\}} \sum_{(t,\mathtt{c})\in\mathcal{I}(\mathtt{a})} \left(\sum_{(i,j)\in\alpha(\kappa,\rho)} \sharp(t, \rho_{[i+1,j]}) \right) \times \mathsf{c} \right) \\
&\quad + \sum_{\kappa \in \mathfrak{R}(\mathtt{v})} \left(\sum_{(i,j)\in\alpha(\kappa,\rho)} \sigma_i(in(\kappa)) + c(\kappa) \right) + \sum_{\mathtt{a}\in atm_2(\kappa)} \sum_{(t,\mathtt{c})\in\mathcal{I}(\mathtt{a})} \sharp(t, \rho) \times \mathsf{c}
\end{aligned}
$$

$$
\begin{aligned}
&\overset{(3b)}{=} \left(\sum_{\mathtt{a}\in \bigcup_{\kappa\in\mathfrak{R}(\mathtt{v})} atm_1(\kappa)\setminus\{\mathtt{v}\}} \sum_{(t,\mathtt{c})\in\mathcal{I}(\mathtt{a})} \sum_{\kappa \in \mathfrak{R}(\mathtt{v}) \text{ s.t. } \mathtt{a}\in atm_1(\kappa)} \left(\sum_{(i,j)\in\alpha(\kappa,\rho)} \sharp(t, \rho_{[i+1,j]}) \right) \times \mathsf{c} \right) \\
&\quad + \sum_{\kappa \in \mathfrak{R}(\mathtt{v})} \left(\sum_{(i,j)\in\alpha(\kappa,\rho)} \sigma_i(in(\kappa)) + c(\kappa) \right) + \sum_{\mathtt{a}\in atm_2(\kappa)} \sum_{(t,\mathtt{c})\in\mathcal{I}(\mathtt{a})} \sharp(t, \rho) \times \mathsf{c}
\end{aligned}
$$

$$
\begin{aligned}
&\overset{(3c)}{=} \left(\sum_{\mathtt{a}\in \bigcup_{\kappa\in\mathfrak{R}(\mathtt{v})} atm_1(\kappa)\setminus\{\mathtt{v}\}} \sum_{(t,\mathtt{c})\in\mathcal{I}(\mathtt{a})} \left(\sum_{\kappa \in \mathfrak{R}(\mathtt{v}) \text{ s.t. } \mathtt{a}\in atm_1(\kappa)} \sum_{(i,j)\in\alpha(\kappa,\rho)} \sharp(t, \rho_{[i+1,j]}) \right) \times \mathsf{c} \right) \\
&\quad + \sum_{\kappa \in \mathfrak{R}(\mathtt{v})} \left(\sum_{(i,j)\in\alpha(\kappa,\rho)} \sigma_i(in(\kappa)) + c(\kappa) \right) + \sum_{\mathtt{a}\in atm_2(\kappa)} \sum_{(t,\mathtt{c})\in\mathcal{I}(\mathtt{a})} \sharp(t, \rho) \times \mathsf{c}
\end{aligned}
$$

$$\overset{(3)}{\leq} \left(\sum_{\mathtt{a} \in \bigcup_{\kappa \in \mathfrak{R}(\mathtt{v})} atm_1(\kappa) \setminus \{\mathtt{v}\}} \sum_{(t,\mathtt{c}) \in \mathcal{I}(\mathtt{a})} \sharp(t,\rho) \times \mathtt{c} \right)$$

$$+ \sum_{\kappa \in \mathfrak{R}(\mathtt{v})} \left(\sum_{(i,j) \in \alpha(\kappa,\rho)} \sigma_i(in(\kappa)) + c(\kappa) \right) + \sum_{\mathtt{a} \in atm_2(\kappa)} \sum_{(t,\mathtt{c}) \in \mathcal{I}(\mathtt{a})} \sharp(t,\rho) \times \mathtt{c}$$

$$\overset{(4)}{\leq} \left(\sum_{\mathtt{a} \in \bigcup_{\kappa \in \mathfrak{R}(\mathtt{v})} atm_1(\kappa) \setminus \{\mathtt{v}\}} \sum_{(t,\mathtt{c}) \in \mathcal{I}(\mathtt{a})} \sharp(t,\rho) \times \mathtt{c} \right)$$

$$+ \sum_{\kappa \in \mathfrak{R}(\mathtt{v})} \left(\sum_{(i,j) \in \alpha(\kappa,\rho)} vb(in(\kappa)) + c(\kappa) \right) + \sum_{\mathtt{a} \in atm_2(\kappa)} \sum_{(t,\mathtt{c}) \in \mathcal{I}(\mathtt{a})} \sharp(t,\rho) \times \mathtt{c}$$

$$\overset{(5a)}{=} \left(\sum_{\mathtt{a} \in \bigcup_{\kappa \in \mathfrak{R}(\mathtt{v})} atm_1(\kappa) \setminus \{\mathtt{v}\}} \sum_{(t,\mathtt{c}) \in \mathcal{I}(\mathtt{a})} \sharp(t,\rho) \times \mathtt{c} \right)$$

$$+ \sum_{\kappa \in \mathfrak{R}(\mathtt{v})} |\alpha(\kappa,\rho)| \times (vb(in(\kappa)) + c(\kappa))$$

$$+ \sum_{\mathtt{a} \in atm_2(\kappa)} \sum_{(t,\mathtt{c}) \in \mathcal{I}(\mathtt{a})} \sharp(t,\rho) \times \mathtt{c}$$

$$\overset{(5)}{\leq} \left(\sum_{\mathtt{a} \in \bigcup_{\kappa \in \mathfrak{R}(\mathtt{v})} atm_1(\kappa) \setminus \{\mathtt{v}\}} \sum_{(t,\mathtt{c}) \in \mathcal{I}(\mathtt{a})} \sharp(t,\rho) \times \mathtt{c} \right)$$

$$+ \sum_{\kappa \in \mathfrak{R}(\mathtt{v})} (\min_{t \in trn(\kappa)} \sharp(t,\rho)) \times \max(vb(in(\kappa)) + c(\kappa), 0)$$

$$+ \sum_{\mathtt{a} \in atm_2(\kappa)} \sum_{(t,\mathtt{c}) \in \mathcal{I}(\mathtt{a})} \sharp(t,\rho) \times \mathtt{c}$$

(1a) Commutativity.
(1b) Distributivity.
(1) We have $atm(\kappa) = atm_1(\kappa) \cup atm_2(\kappa)$, $atm_1(\kappa) \cap atm_2(\kappa) = \emptyset$ and $\mathtt{v} \in atm_1(\kappa)$ (Definition 22).
(2) With Lemma A.6 we have that all intervals in $\alpha(\kappa,\rho)$ are pairwise disjoint. Therefore $\sum_{(i,j) \in \alpha(\kappa,\rho)} \sharp(t, \rho_{[i+1,j]}) \leq \sharp(t,\rho)$. Further note that $\mathtt{c} > 0$ for $(\_, \mathtt{c}) \in \mathcal{I}(\mathtt{a})$.
(3a) Commutativity.
(3b) Commutativity.
(3c) Distributivity.
(3) Let $\kappa_1, \kappa_2 \in \mathfrak{R}(\mathtt{v})$. Assume $\mathtt{a} \in atm_1(\kappa_1) \cap atm_1(\kappa_2)$ and $\mathtt{a} \neq \mathtt{v}$. By Definition 22 there is exactly one path in the reset graph from $\mathtt{a}$ to $\mathtt{v}$. Thus κ_1 and κ_2 have a common suffix: they share the single path from $\mathtt{a}$ to $\mathtt{v}$ in the reset graph. We therefore have by Lemma A.6 that all intervals in $\alpha(\kappa_1,\rho) \cup \alpha(\kappa_2,\rho)$ are

pairwise disjoint. Therefore $\sum\limits_{\kappa\in\mathfrak{R}(\mathtt{v}) \text{ s.t. } \mathtt{a}\in atm_1(\kappa)} \sum\limits_{(i,j)\in\alpha(\kappa,\rho)} \sharp(t,\rho_{[i+1,j]}) \leq \sharp(t,\rho)$. Further note that $\mathtt{c} > 0$ for $(\_,\mathtt{c}) \in \mathcal{I}(\mathtt{a})$.

(4) Let $\kappa \in \mathfrak{R}(\mathtt{v})$. By assumption $vb(in(\kappa))$ denotes a variable bound for $in(\kappa)$ on ρ.

(5a) With $\sum\limits_{(i,j)\in\alpha(\kappa,\rho)} vb(in(\kappa)) + c(\kappa) = |\alpha(\kappa,\rho)| \times (vb(in(\kappa)) + c(\kappa))$

(5) Let $\kappa \in \mathfrak{R}(\mathtt{v})$. Let $(i_1,j_1),(i_2,j_2) \in \alpha(\kappa,\rho)$. We have by Lemma A.6 that all intervals in $\alpha(\kappa,\rho)$ are pairwise disjoint. Further each transition $t \in trn(\kappa)$ appears at least once on each sub-run $\rho_{[i,j]}$ with $(i,j) \in \alpha(\kappa,\rho)$. Therefore: $|\alpha(\kappa,\rho)| \leq \min\limits_{t\in trn(\kappa)} \sharp(t,\rho)$.

C)

$$\begin{aligned}
\sharp(\tau,\rho) &\overset{(1)}{\leq} \left(\sum_{(t,\mathtt{c})\in\mathcal{I}(\mathtt{v})} \sharp(t,\rho)\times\mathtt{c}\right) + \left(\sum_{\mathtt{a}\in\bigcup\limits_{\kappa\in\mathfrak{R}(\mathtt{v})} atm_1(\kappa)\setminus\{\mathtt{v}\}} \sum_{(t,\mathtt{c})\in\mathcal{I}(\mathtt{a})} \sharp(t,\rho)\times\mathtt{c}\right) \\
&\quad + \sum_{\kappa\in\mathfrak{R}(\mathtt{v})} (\min_{t\in trn(\kappa)} \sharp(t,\rho)) \times \max(vb(in(\kappa)) + c(\kappa), 0) \\
&\qquad + \sum_{\mathtt{a}\in atm_2(\kappa)} \sum_{(t,\mathtt{c})\in\mathcal{I}(\mathtt{a})} \sharp(t,\rho)\times\mathtt{c} \\
&\overset{(2)}{=} \left(\sum_{\mathtt{a}\in\bigcup\limits_{\kappa\in\mathfrak{R}(\mathtt{v})} atm_1(\kappa)} \sum_{(t,\mathtt{c})\in\mathcal{I}(\mathtt{a})} \sharp(t,\rho)\times\mathtt{c}\right) \\
&\quad + \sum_{\kappa\in\mathfrak{R}(\mathtt{v})} (\min_{t\in trn(\kappa)} \sharp(t,\rho)) \times \max(vb(in(\kappa)) + c(\kappa), 0) \\
&\qquad + \sum_{\mathtt{a}\in atm_2(\kappa)} \sum_{(t,\mathtt{c})\in\mathcal{I}(\mathtt{a})} \sharp(t,\rho)\times\mathtt{c}
\end{aligned}$$

(1) With A) and B).

(2) We have $\mathcal{R}(\mathtt{v}) \neq \emptyset$ by well-definedness of $\Delta\mathcal{P}$ and therefore $\mathfrak{R}(\mathtt{v}) \neq \emptyset$. Further $\mathtt{v} \in atm_1(\kappa)$ for all $\kappa \in \mathfrak{R}(\mathtt{v})$. □

A.2.1 Proof of Theorem 2

We prove the more general claim formulated in Theorem A.2.

Theorem A.2 (Soundness of Bound Algorithm based on Reset Chains) *Let $\Delta\mathcal{P}(L,E,l_b,l_e)$ be a well-defined and fan-in free DCP over atoms $\mathcal{A}$ with a reset dag. Let Ξ be a set of runs of $\Delta\mathcal{P}$ that is closed under* normalization. *Let $\zeta : E \mapsto Expr(\mathcal{A})$ be a* local bound mapping *for all $\rho \in \Xi$. Let $T\mathcal{B}$ and $V\mathcal{B}$ be defined as in Definition 23. Let $\tau \in E$ and $\mathtt{a} \in \mathcal{A}$. Let $\rho \in \Xi$. Let σ_0 be the initial state of ρ. We have: (I) $[\![T\mathcal{B}(\tau)]\!](\sigma_0)$ is a* transition bound *for τ on ρ. (II) $[\![V\mathcal{B}(\mathtt{a})]\!](\sigma_0)$ is a* variable bound *for $\mathtt{a}$ on ρ.*

Proof Let $\rho = (\sigma_0,l_0) \xrightarrow{u_0} (\sigma_1,l_1) \xrightarrow{u_1} \cdots \in \Xi$.

If $[\![T\mathcal{B}(\tau)]\!] = \infty$ (I) holds trivially. If $[\![V\mathcal{B}(\mathtt{a})]\!] = \infty$ (II) holds trivially.

Assume $[\![T\mathcal{B}(\tau)]\!] \neq \infty$ and $[\![V\mathcal{B}(\mathsf{a})]\!] \neq \infty$. Then in particular the computation of $T\mathcal{B}(\tau)$ resp. $V\mathcal{B}(\mathsf{a})$ terminate. We proceed by induction over the call tree of $T\mathcal{B}(\tau)$ resp. $V\mathcal{B}(\mathsf{a})$.

Base Case: As in the proof of Theorem A.1 (Section A.1.1).

Step Case:
I) As in the proof of Theorem A.1 (Section A.1.1).
II)

$$\begin{aligned}
\sharp(\tau,\rho) \overset{(1)}{\leq} & \left(\sum_{b \in \bigcup_{\kappa \in \mathfrak{R}(\zeta(\tau))} atm_1(\kappa)} \sum_{(t,\mathsf{c}) \in \mathcal{I}(b)} \sharp(t,\rho) \times \mathsf{c} \right) \\
& + \sum_{\kappa \in \mathfrak{R}(\zeta(\tau))} (\min_{t \in trn(\kappa)} \sharp(t,\rho)) \times \max([\![V\mathcal{B}(in(\kappa))]\!](\sigma_0) + c(\kappa), 0) \\
& \qquad + \sum_{b \in atm_2(\kappa)} \sum_{(t,\mathsf{c}) \in \mathcal{I}(b)} \sharp(t,\rho) \times \mathsf{c}
\end{aligned}$$

$$\begin{aligned}
\overset{(2)}{\leq} & \left(\sum_{b \in \bigcup_{\kappa \in \mathfrak{R}(\zeta(\tau))} atm_1(\kappa)} \sum_{(t,\mathsf{c}) \in \mathcal{I}(b)} \sharp(t,\rho) \times \mathsf{c} \right) \\
& + \sum_{\kappa \in \mathfrak{R}(\zeta(\tau))} (\min_{t \in trn(\kappa)} [\![T\mathcal{B}(t)]\!](\sigma_0)) \times \max([\![V\mathcal{B}(in(\kappa))]\!](\sigma_0) + c(\kappa), 0) \\
& \qquad + \sum_{b \in atm_2(\kappa)} \sum_{(t,\mathsf{c}) \in \mathcal{I}(b)} \sharp(t,\rho) \times \mathsf{c}
\end{aligned}$$

$$\begin{aligned}
\overset{(3)}{\leq} & \left(\sum_{b \in \bigcup_{\kappa \in \mathfrak{R}(\zeta(\tau))} atm_1(\kappa)} \sum_{(t,\mathsf{c}) \in \mathcal{I}(b)} \sharp(t,\rho) \times \mathsf{c} \right) \\
& + \sum_{\kappa \in \mathfrak{R}(\zeta(\tau))} [\![T\mathcal{B}(trn(\kappa))]\!](\sigma_0) \times \max([\![V\mathcal{B}(in(\kappa))]\!](\sigma_0) + c(\kappa), 0) \\
& \qquad + \sum_{b \in atm_2(\kappa)} \sum_{(t,\mathsf{c}) \in \mathcal{I}(b)} \sharp(t,\rho) \times \mathsf{c}
\end{aligned}$$

$$\begin{aligned}
\overset{(4)}{\leq} & \left(\sum_{b \in \bigcup_{\kappa \in \mathfrak{R}(\zeta(\tau))} atm_1(\kappa)} \sum_{(t,\mathsf{c}) \in \mathcal{I}(b)} [\![T\mathcal{B}(t)]\!](\sigma_0) \times \mathsf{c} \right) \\
& + \sum_{\kappa \in \mathfrak{R}(\zeta(\tau))} [\![T\mathcal{B}(trn(\kappa))]\!](\sigma_0) \times \max([\![V\mathcal{B}(in(\kappa))]\!](\sigma_0) + c(\kappa), 0) \\
& \qquad + \sum_{b \in atm_2(\kappa)} \sum_{(t,\mathsf{c}) \in \mathcal{I}(b)} [\![T\mathcal{B}(t)]\!](\sigma_0) \times \mathsf{c}
\end{aligned}$$

$$\stackrel{(5)}{=} [\![\texttt{Incr}(\bigcup_{\kappa \in \mathfrak{R}(\zeta(\tau))} atm_1(\kappa))]\!](\sigma_0)$$
$$+ \sum_{\kappa \in \mathfrak{R}(\zeta(\tau))} [\![T\mathcal{B}(trn(\kappa))]\!](\sigma_0) \times \max([\![V\mathcal{B}(in(\kappa))]\!](\sigma_0) + c(\kappa), 0)$$
$$+ [\![\texttt{Incr}(atm_2(\kappa))]\!](\sigma_0)$$

$$\stackrel{(6)}{=} [\![T\mathcal{B}(\tau)]\!](\sigma_0)$$

(1) By Lemma A.7: Since Ξ is closed under *normalization* we have that $\zeta(\tau)$ is a *local bound* for τ on $\lfloor \rho \rfloor$. Further: Let $\kappa \in \mathfrak{R}(\zeta(\tau))$. We have that $V\mathcal{B}(in(\kappa))$ is called during the computation of $T\mathcal{B}(\tau)$ (Definition 23). Note that with $[\![T\mathcal{B}(\tau)]\!] \neq \infty$ also $[\![V\mathcal{B}(in(\kappa))]\!] \neq \infty$. By I.H. $[\![V\mathcal{B}(in(\kappa))]\!](\sigma_0)$ is a *variable bound* for $in(\kappa)$.

(2) Let $\kappa \in \mathfrak{R}(\zeta(\tau))$. Let $t \in trn(\kappa)$. We have that $T\mathcal{B}(t)$ is called during the computation of $T\mathcal{B}(\tau)$. Thus for $t \in trn(\kappa)$ with $[\![T\mathcal{B}(t)]\!] \neq \infty$ we have that $[\![T\mathcal{B}(t)]\!](\sigma_0)$ is a *transition bound* for t on ρ by I.H.. Note that with $[\![T\mathcal{B}(\tau)]\!] \neq \infty$ there is a $t \in trn(\kappa)$ s.t. $[\![T\mathcal{B}(t)]\!] \neq \infty$. Thus $\min_{t \in trn(\kappa)} \sharp(t, \rho) \leq \min_{t \in trn(\kappa)} [\![T\mathcal{B}(t)]\!](\sigma_0)$.

(3) With $T\mathcal{B}(trn(\kappa)) = \min_{t \in trn(\kappa)} T\mathcal{B}(t)$ (Definition 23) and Definition 15.

(4) Let $\kappa \in \mathfrak{R}(\zeta(\tau))$. Let $b \in atm(\kappa)$. Let $(t, \_) \in \mathcal{I}(b)$. We have that $T\mathcal{B}(t)$ is called when computing $T\mathcal{B}(\tau)$ (Definition 23). Note that with $[\![T\mathcal{B}(\tau)]\!] \neq \infty$ also $[\![T\mathcal{B}(t)]\!] \neq \infty$. By I.H. $\sharp(t, \rho) \leq [\![T\mathcal{B}(t)]\!](\sigma_0)$.

(5) Definition 23 and Definition 15.

(6) Definition 23 and Definition 15. □
